# Publisher Correction: Mineralogical controls on PFAS and anthropogenic anions in subsurface soils and aquifers

**DOI:** 10.1038/s41467-025-58754-x

**Published:** 2025-04-10

**Authors:** Marina G. Evich, James Ferreira, Oluwaseun Adeyemi, Paul A. Schroeder, Jason C. Williams, Brad Acrey, Diana Burdette, Malcolm Grieve, Michael P. Neill, Kevin Simmons, Brian C. Striggow, Samuel B. Cohen, Mike Cyterski, Donna A. Glinski, W. Matthew Henderson, Du Yung Kim, John W. Washington

**Affiliations:** 1 USEPA, Office of Research and Development, Center for Environmental Measurement and Modeling, Athens, GA USA; 2 USEPA, Region 4, Atlanta, GA USA; 3 Department of Geology, University of Georgia, Athens, GA USA; 4 South Carolina Department of Health and Environmental Control, Bureau of Land and Waste Management, Columbia, SC USA; 5 USEPA, Region 4, Laboratory Services and Applied Sciences Division, Athens, GA USA

**Keywords:** Geochemistry, Chemistry

Correction to: *Nature Communications* 10.1038/s41467-025-58040-w, published online 01 April 2025

In this article the title for Table 1 was inadvertently swapped and should have read ‘Summary of B horizon mineral(oid) content for soils in USDA database’. In addition, the following footnote for Table 1 was inadvertently removed: "(1) This USDA semi-quantitative scale of textural clay proportions is defined by x-ray peak height (counts/s) according to: 1=very small (<110); 2=small (110-360); 3=medium (360-1120); 4=large (1120-1800); 5=very large (>1800). Montmorillonite is a 2:1 smectite clay. Column and row titles are emboldened to guide readers’ eye". The original article has been corrected.

