# Supplementary material for: Mineralogical controls on PFAS and anthropogenic anions in subsurface soils and aquifers
Source: Nat Commun. 2025 Apr 1;16:3118. doi: 10.1038/s41467-025-58040-w (PMC11962083; doi:10.1038/s41467-025-58040-w)
Supplement: Supplementary file 1 — Supplementary Information [file 41467_2025_58040_MOESM1_ESM.pdf]

Supplementary Information for:

Mineralogical controls on PFAS and anthropogenic anions in subsurface soils and aquifers

March 6, 2025

Marina G. Evich[1], James Ferreira[2], Oluwaseun Adeyemi[3], Paul A. Schroeder[3], Jason C. Williams[4], Brad Acrey[5], Diana Burdette[5], Malcolm Grieve[5], Michael P. Neill[5], Kevin Simmons[5], Brian C. Striggow[5], Samuel B. Cohen[1], Mike Cyterski[1], Donna A. Glinski[1], W. Matthew Henderson[1], Yung Kim[1], John W. Washington[1,3]**

[1] *USEPA, Office of Research and Development, Center for Environmental Measurement and Modeling, 960 College Station Road, Athens, GA 30605*
[2] *USEPA, Region 4, Superfund and Emergency Management Division, 61 Forsyth Street SW, Atlanta, GA 30303*
[3] *University of Georgia, Department of Geology, 210 Field Street, Athens, GA 30602-2501*
[4] *South Carolina Department of Health and Environmental Control, Bureau of Land and Waste Management, 2600 Bull Street, Columbia, SC 29201*
[5] *USEPA, Region 4, Laboratory Services and Applied Sciences Division, 980 College Station Road, Athens, GA 30605*

Contents:

I. Supplementary Methods

- Site description
    - Supplementary Fig. 1: Site locations and sludge loads
- Soil coring and sampling
- Groundwater sampling
- Soil-characterization data
- X-ray diffraction
- Selective-extraction of amorphous Fe, Al and Mn hydrous-oxide mineraloids
- Soil and Water Analysis for PFAS
- Interrogation of USDA soil database for mineral content
    - Supplementary Table 1: Soil sample characteristic/analytes and corresponding NCSS database name
    - Supplementary Table 2: Studied soil orders
- Calculation of Fe and Al (oxy)hydroxide mineral(oid) electrostatic charge
    - Supplementary Equations 1-16: Expressing electrostatic surface charge
    - Supplementary Equations 17-18: Estimating ionic strength
    - Supplementary Table 3: Tabulation of Log P for HFO from Dzombak and Morel's Table 8.8
    - Supplementary Fig. 2: Quadratic regression of Log P for HFO as a function of pH, at ionic strengths 0.5-0.005 M
    - Supplementary Table 4: Tabulation of Log P for Gibbsite from MINEQL+ V5.0 (personal communication from Dave Dzombak)
    - Supplementary Fig. 3: Quadratic regression of Log P for gibbsite as a function of pH, at ionic strengths 0.5-0.005 M
    - Supplementary Table 5: Properties to model mineral(oid) surface charge
- Calculation of kaolinite electrostatic charge
    - Supplementary Table 6: Tabulation of Log P for Kaolinite approximated from Gibbsite by $\text{Log } P_{kaolinite} = \text{Log } P_{gibbsite} + 2.18$
    - Supplementary Fig. 4: Quadratic regression of Log P for kaolinite as a function of pH, at ionic strengths 0.5-0.005 M
- Calculation of vadose moisture content and air-water interface
    - Supplementary Equations 19-20: Estimating air-water interface
    - Supplementary Equation 21: Estimating fraction saturation from height above water table
    - Supplementary Equation 22: Estimating porosity from bulk density
- Transformations and filtering of variables for data analysis

II. Data Summary

- Supplementary Table 7: Geochemical soil parameters for Colluvial Field 1
- Supplementary Table 8: Geochemical soil parameters Residual Field 2
- Supplementary Table 9: Geochemical groundwater parameters
- Supplementary Table 10: pH-Dependent Electrostatic Surface Charge of Selected Mineral(oid)s in Field 1

- Supplementary Table 11: pH-Dependent Electrostatic Surface Charge of Selected Mineral(oid)s in Field 2
- Supplementary Table 12: Moisture status, Field 1
- Supplementary Table 13: Moisture status, Field 2
- Supplementary Table 14: Soil PFAS in Field 1
- Supplementary Table 15: Soil PFAS in Field 2
- Supplementary Table 16: Groundwater PFAS in Field 1
- Supplementary Table 17: Groundwater PFAS in Field 2
- Supplementary Table 18: Surface water PFAS downgradient of Fields 1 & 2

III. Extended Geochemical Summary

- Supplementary Fig. 5: X-ray major minerals in residuum Field 2
- Supplementary Table 19: Summary of USDA/NCSS mineralogy data by Soil Order in soil horizon A
- Supplementary Table 20: Summary of USDA/NCSS mineralogy data by Soil Order in soil horizon B
- Supplementary Table 21: Summary of USDA/NCSS mineralogy data by Soil Order in soil horizon C
- Supplementary Fig. 6: B-horizon x-ray diffraction (XRD) scale vs Soil Order
- Supplementary Fig. 7: Pourbaix (Eh-pH) diagram depicting stability fields for goethite and amorphous $Fe(OH)_3$
- Supplementary Fig. 8: Thermodynamic speciation diagram for aluminum
- Supplementary Fig. 9: Electrostatic charge distribution in the residuum soil column of Field 2
- Supplementary Equations 23-27: Calculating Fe^{2+} oxidation rate half-life from dissolved O_2 concentration
- Supplementary Fig. 10: Half-life of ferrous oxidation by O_2 at P_{O2} = 0.208 atm
- Supplementary Fig. 11: Heat map of nominal correlations amongst geochemical properties for Field 1
- Supplementary Fig. 12: Heat map of nominal correlations amongst geochemical properties for Field 2

IV. Extended PFAS Summary

- Supplementary Fig. 13: Concentration of PFAS (ng/kg dry soil) for all hand augured (SF) and soil boring (SB) surface soils collected in 2021, from 0-0.15 m (0-0.5 ft) bgs (A-F) and relative to surface soils collected in 2018 (G-I)
- Supplementary Fig. 14: Depth profile for PFCA concentrations in Field 1 with depth from surface to ~20 m for each analyte in ng/kg dry soil (orange) and the shallow groundwater in ng/L (blue)
- Supplementary Fig. 15: Depth profile for PFCA concentrations in Field 2 with depth from surface to ~20 m for each analyte in ng/kg dry soil (orange) and the shallow groundwater in ng/L (blue).
- Supplementary Fig. 16: Radar plots of normalized PFCA concentrations (inside from 0 to 1) with depth (outer from 0.1 to ~ 20, in meters) of Field 1 PFBA-PFNA (A) and PFDA-PFTeDA (B) and Field 2 PFBA-PFNA (C) and PFDA-PFTrDA (D)
- Supplementary Fig. 17: Heat map of Field 1 PFAS
- Supplementary Fig. 18: Heat map of Field 2 PFAS

- Supplementary Fig. 19: Sum of all PFAS with depth for only soil borings – field 1 sum is ~ 3-fold higher than field 2, with notable exceptions: PFOA and PFBS
- Supplementary Fig. 20: Short-chain PFAS mobility in subsurface is controlled by electrostatic sorption, dominantly on pH-dependent charged surfaces of Al minerals
- Supplementary Fig. 21: PFCA vertical distribution in colluvial Field 1, with air-water interface and TOC (clustering with long-chain PFCAs in surface soil) and aluminum-oxide charge (clustering with short-chain PFAS in subsurface soil)
- Supplementary Fig. 22: PFSA vertical distribution in colluvial Field 1, with air-water interface and TOC (clustering with long-chain PFSAs in near-surface soil) and aluminum-oxide charge (clustering with short-chain PFSAs in subsurface soil)
- Supplementary Fig. 23: PFCA vertical distribution in residual Field 2, with air-water interface and TOC (clustering with long-chain PFCAs in surface soil) and aluminum-oxide charge (clustering with short-chain PFAS in subsurface soil)
- Supplementary Fig. 24. Correlation table/heat map for Field 1+ Field 2 data combined, revealing robust trends of PFAS with geochemical parameters for surface through truncated depth at 10.36 m (34 ft) (All Depths), for surface to the water table above 12 ft (Unsaturated Media), and below the water table, from 5.49 – 10.36 m (18 – 34 ft) (Saturated Media)
- Supplementary Fig. 25. Correlation table/heat map for Field 1 PFAS with geochemical parameters for surface through truncated depth at 10.36 m (34 ft) (All Depths), for surface to the water table above 12 ft (Unsaturated Media), and below the water table, from 5.49 – 10.36 m (18 – 34 ft) (Saturated Media)
- Supplementary Fig. 26. Correlation table/heat map for Field 2 PFAS with geochemical parameters for surface through truncated depth at 8.53 m (28 ft) (All Depths), for surface to the water table above 14 ft (Unsaturated Media), and below the water table, from 4.27 – 8.53 m (14 - 28 ft) (Saturated Media)

V. Supplementary References

I. Methods

Site description:

For nearly 50 years, a textile mill, formerly known as Galey & Lord, operated in Darlington County, South Carolina, producing textiles that were treated with PFAS for stain resistance whilst generating waste sludge contaminated with PFAS. Between 1993 and 2013, over 45,500 dry tons of this waste sludge was applied to regional agricultural fields, including a total of 303 individual parcels, totaling more than 9,875 acres. While the median parcel received 2.7 tons/acre, 18% of parcels received over 10 tons/acre, and 6 of the parcels received over 20 tons/acre. Additionally, 78% of all applications occurred prior to 2002, with over half of all applied sludge applied in a 5-year window, from 1998-2002. In efforts to evaluate a broader scope of PFAS migration in the subsurface through the vadose zone and water-table aquifer, two proximate fields were selected for coring based on differences in PFAS loading rate, temporal application rates, and pedogenesis, Supplementary Fig. 1.

Both selected fields, Field 1 and Field 2, received over the median application rate for all impacted parcels: Field 1 was among the highest recipients of textile mill sludge, receiving over 1,760 dry tons of biosolids, for an application rate of 26 tons/acre, while Field 2 was less heavily applied, receiving over 285 dry tons of biosolids, at an application rate of 7 tons/acre (Supplementary Fig. 1b-c). Similarly, there are slight temporal differences in annual biosolid loadings. Field 1 received biosolids for 13 years, with just over half of total biosolids applied from 1998-2002 (median application year 2000) and > 37% of total biosolids from 2005-2012 (median application year 2009). Field 2 received almost all, > 94%, of applied biosolids from 1998-2002 (median application year 2000).

Situated in the Middle Atlantic Coastal Plain physiographic province, the exact site locations are left unidentified here to preserve the anonymity of the property owners. Geologically, the sites are mapped near the up-dip western limit of the lower Pliocene-aged (2.8 to 3.6 millions of years before present, MYBP) Duplin Formation, which is mainly composed of unconsolidated marine sediments [1]. The Duplin has sandy facies, with quartz dominating the sand textural fraction. Commonly kaolinite is a dominant authigenic clay mineral, with occurrences of gibbsite [1] also reflecting considerable weathering. Pedologically, Field 1 is mapped as the Emporia loamy sand USDA soil series, 6 to 10% slopes (fine-loamy, siliceous, subactive, thermic Typic Hapludult), very deep and well drained [2]. Field 2 is mapped as the Norfolk loamy sand USDA soil series 0 to 2% slopes (fine-loamy, kaolinitic, thermic Typic Kandiudult), very deep and well drained [2]. On site, Field 1 drilling and sampling was located at mid-slope on the axis of a local natural concave drainage depression in a feral pasture, with the soil expected to have been subject to local slumping with attendant limited colluvial vertical mixing, and Field 2 was

located on a nearly horizontal surface in a crop-rotated field (peanuts at time of sampling), with the soil thought to have developed as in-place residuum.

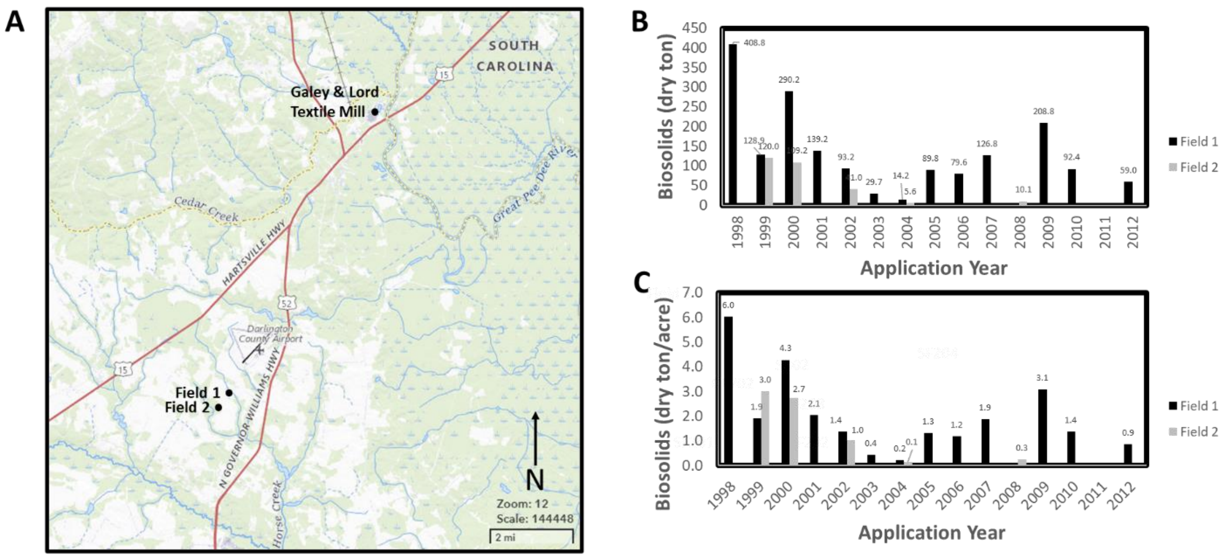

Supplementary Fig. 1. **Site locations and sludge loads.** (**a**) Locations relative to the source textile mill. Sludge loading on colluvial Field 1 and residual Field 2 in total biosolids loaded (**b**) and biosolids per acre (**c**) by application year. Map is open-source from the US Geological Survey, available at https://apps.nationalmap.gov/downloader/#/.

Soil coring and sampling:

Subsurface soil samples were collected with a Geoprobe 6620 tracked rig using direct-push technology (DPT) which employs a percussive hydraulic hammer to drive a sampler and stacked threaded drill rods to depth. In DPT soil sampling, only the sample material is retrieved to the surface and all other displaced soil material is compressed into the borehole walls. In this work, 1.5 m (5 ft.) Macrocore piston samplers were equipped with a tapered point held at the bottom of a hollow sample barrel. The tapered point pushes all soil material aside until the top of the sampling interval is reached where the point is released to move freely into the barrel so that the sample material can pass through the annular cutting shoe and a polyvinyl chloride (PVC) core-catcher, then enter the barrel where it is contained in a 4.5 cm (1.75 in.) diameter single-use PVC liner. Sample pushes were limited to 1.2 m (4 ft.) intervals to prevent over-packing of the 1.5 m sample barrels. Upon sample collection, the sampler is brought to the surface,

the PVC liner containing sample material is removed from the barrel for processing, and the process repeated for subsequent samples from greater depths.

At a sample processing station, the liners were lain horizontally and split open lengthwise to expose the intact core. After recording field texture and color descriptions, the sample material was transferred to glass pans, disaggregated and mixed with stainless-steel spoons. Pans and spoons were dedicated to single samples.

At each study site, cores were collected from the ground surface, through the vadose zone and the water-table aquifer to roughly 20 m below ground surface (bgs), where a clay aquiclude was expected (Field 1, cores were collected to 19.5 m or 64 ft bgs) or encountered (Field 2, cores were collected to 20.1 m or 66 ft bgs). Incremental samples were 15-cm (6-in) for depths 0 to 1.5 m (5 ft), 30-cm (1 ft) for 1.5 m to 3.0 m (5 ft to 10 ft), and 0.6-m (2 ft) over 3 m to the termination of drilling.

In addition to soil coring samples, to gauge spatial heterogeneity, four additional 0-15 cm samples were collected by hand auger from each study site, at less than roughly 20 m from drilling, so that five total surface-soil samples were collected from each study site, including the initial boring increment from the cores.

Groundwater and surface water sampling:

At each sample site, roughly 30 m from the soil coring, a temporary well was driven to moderate depth to establish static water level. After static water level was established, a screen interval was chosen to represent shallow groundwater, a well was driven, a Geoprobe SP15™ PVC screen was installed, and water was sampled as described below. After being informed by the soil coring, we selected an interval representing the deep aquifer, drove the deep well, and installed the screen. Before sampling, the wells were developed until discharge water approached visual clarity, the sample tubing was set to near the top of the water column while pumping to flush any remaining sediment from the water column prior to sampling. Samples were collected from the pump discharge after specific conductance and pH had stabilized and turbidity had dropped to below 10 nephelometric turbidity units (NTU). After water sampling was complete, all boreholes were decommissioned by reentry pressure grouting with a 30% solids bentonite slurry.

Water sampling was performed by pumping at <1 L/min, using a peristaltic pump equipped with polyethylene tubing leading to a short section of Silastic™ tubing at the pump head, until temperature, pH, specific conductance, dissolved oxygen and turbidity had stabilized. These values were recorded, and

water samples collected for analyses. Roughly 500 mL of surface water was collected in the vicinity of each field in high-density polypropylene (HDPE) vessels. Two samples were collected downgradient of Field 1 and two samples were collected downgradient of Field 2. A final sample was collected downstream of the Field 2 streams and below confluence with the stream fed by headwaters in Field 1.

Soil-characterization data:

Conventional soil-characterization data were generated on all soil samples at the University of Georgia, Agricultural and Environmental Services Laboratory (AESL), including soil pH, cation exchange capacity, base saturation, lime requirement, Mehlich 1 metals (Ca, Mg, Na, K, P, Fe, Mn, Cu, Ni, Cd, Pb, Zn, Cr, Mo), water-extractable anions (NO_3^-, PO_4^{-2}, Cl^-, F^-, SO_4^{-2}), total organic carbon, total nitrogen, and textural sand, silt, and clay. Methods for these analyses are reported in the AESL Handbook [3].

X-ray diffraction:

Bulk x-ray diffraction analyses (XRD) were performed on all samples and mineral concentrations quantitated [4] using the Rietveld method [5]. Summarizing, soil samples were dried overnight at 65 ºC, ground in a corundum mortar and McCrone Micronizing mill®, then compressed and analyzed on a Bruker D8 Advance X-ray Diffractometer. A cobalt radiation source was applied, with an iron filter to reduce K_β radiation interference. Alignment and calibration were confirmed with a NIST Reference Standard (SRM1976b). Raw data were processed with the Bruker Eva® program for mineral identification. Semi-quantitation of mineral abundance was carried out by the Reitveld method to fit calculated signal intensity to observed intensity [5] with mineral weight fraction as one of the minimization variables [6] using TOPAS® software.

In more detail, approximately 15 grams of each wet sample were taken for bulk mineralogy and dried in an oven at 65 °C overnight. A corundum mortar was used to manually grind about 7 grams of the dried sample. To further reduce the powder's particle size to an average of 5 to 10 µm, 10 ml of ethyl alcohol was added to the powder and processed in a McCrone Micronizing mill® for 10 minutes. Samples were backfilled against a square plate glass into a 2.5 x 2.5 cm aluminum holder after being dried to eliminate alcohol in a 65 °C oven overnight. To create a flat, self-supporting mount, the powder was compressed against a glass plate at 27 bar (400 psi) to retain the sample geometry tangential to the instrument-focusing circle and reduce sample transparency, and then analyzed on a Bruker D8 Advance

X-ray Diffractometer using a 250 cm goniometer radius, a 0.6 mm divergent slit, and Bragg-Brentano geometry. To reduce low-angle dispersion into the position-sensitive Lynx-Eye® detector, a knife-edge blade was put 2 mm over the sample's surface. To reduce K_{β} radiation, an iron filter was utilized in conjunction with a cobalt radiation source ($K_{\alpha 1}$ = 1.7890Å and $K_{\alpha 2}$ = 1.7928Å) that was operated at 35 kV and 40 mA. To verify alignment and calibration within 0.01° 2Θ tolerance of the certificate value for the brightest reflection peak position, an external NIST Reference Standard SRM1976b corundum (α-Al_2O_3) was used. Using a locked-coupled continuous scan mode with a step size of 0.01° 2Θ and a count rate of 0.2 seconds per step, the scan range was enhanced from 2 to 70° 2Θ. Raw data were processed with the Bruker Eva® program, which presents raw data and graphs, or diffractograms, with patterns and peak positions identified. The raw data was $K_{\alpha 2}$ stripped using an Eva® algorithm with the wavelengths above and a relative intensity ratio of 1.0:0.5. Peak locations were compared to information from the powder diffraction file (PDF+4) database of the International Centre for Diffraction Data (ICDD). Eva software was used with the 2022-PDF+4 database to identify the best-fit phases for mineral identification. A structure file containing the unit-cell lattice parameters, atomic species, and atomic positions was exported for each phase found. Semi-quantitative models of samples were analyzed using TOPAS® software. The Rietveld refining method is the basis of this program (Reitveld 1966). Using kinematic diffraction theory, this method, in essence, determines the theoretical diffraction pattern for each phase (Schroeder 2018). The disparity between observed XRD data and predicted patterns was then reduced via optimization. Additionally, the procedure determines total XRD intensity, associated with abundance. It is important to highlight that mixed-layer structures cannot be considered in the TOPAS® software calculations. As a result, despite additional optimization attempts in some circumstances, adequate R_{wp} values were not obtained. Despite this restriction, consistent procedures were employed to maintain model parameters with comparable ranges of values to enable inter-sample comparison of relative abundances.

For the fine-clay fraction, 10 grams of dried sample were added to a centrifuge tube containing 38 grams of sodium hexametaphosphate and 8 grams of sodium carbonate in 1 liter of deionized water. The mixture was stirred for about 1 minute using a Branson Sonifier Cell Disrupter 350. Samples were then sieved to eliminate the sand fraction (>63 µm, 230 mesh). Using conventional centrifugation methods, the silt fraction ≤63 µm, was separated from the 2 µm clay fraction [6]. The resulting slurry was deemed to be Na-saturated. For Mg-saturated samples, this process was repeated by exchanging in 0.1 M $MgCl_2$ solutions. The slurry was centrifuged to settle all the particles, and the solution renewal and centrifugation processes were repeated until full saturation was achieved. Deionized water was added to the slurry, followed by additional centrifugation to eliminate excess salt [7]. This fine fraction was mixed with 25 to 30 ml of deionized water, pipetted onto a glass petrographic slide (25 mm × 40 mm), and air-dried over-

night. The sample was kept in ethylene glycol (EG) overnight in a closed ethylene glycol atmosphere and heated in the oven for an hour at 110 °C, 350 °C, and 550 °C. During the drying process, orientated particles were produced to improve the phyllosilicate/clay minerals' basal reflections.

Selective-extraction of amorphous Fe, Al, and Mn hydrous-oxide mineraloids:

The soils were extracted with a 0.25 M hydroxylamine hydrochloride – 0.25 M hydrochloric acid solution at 50 °C as previously reported by Chao and Zhou [8], except the extraction was extended from the reported 30 min to one hour, a period in which extraction efficacy had plateaued and was insensitive to small timing variability. This procedure yields results in close agreement with the widely used 'oxalate in the dark' extraction [8]. The procedure is less aggressive than the extraction of Poulton and Canfield [9], who employed 1 M hydroxylamine hydrochloride – 25% acetic acid for 48 hr to extract amorphous hydrous oxides plus crystalline ferrihydrite and lepidocrocite. Extracts were analyzed by inductively coupled plasma mass spectrometry [10].

Soil and Water Analysis for PFAS:

Soil samples were extracted and analyzed using ASTM D7968: Standard Test Method for Determination of Polyfluorinated Compounds in Soil by Liquid Chromatography Tandem Mass Spectrometry (LC/MS/MS) [11]. Surface water was extracted by solid phase extraction using methanol and weak anion exchange (WAX) cartridges [12], then analyzed on an Agilent 1290 Infinity II UPLC system coupled to 6495 Triple Quad in negative electrospray-ionization mode. Chromatographic separation was achieved using a Waters Acquity BEH C18 column (1.7 µm, 2.1 x 100 mm). Groundwater was extracted and analyzed using ASTM D7979: Standard Test Method for Determination of Per- and Polyfluoroalkyl Substances in Water, Sludge, Influent, Effluent, and Wastewater by Liquid Chromatography Tandem Mass Spectrometry (LC/MS/MS) [13]. Targeted analyses were performed on two Waters Corporation (Milford, MA) Acquity UPLC systems coupled to Xevo TQ-S micro and Xevo TQ-XS MS/MS operated in negative electrospray-ionization mode. Chromatographic separation was achieved using a Waters Acquity CSH Phenyl-Hexyl column (1.7 µm, 2.1 x 100 mm). Nontargeted analyses were performed on selected soil sample extracts as previously described on a Waters Acquity UPLC system coupled to a Xevo quadrupole time-of-flight high resolution MS in negative electrospray-ionization mode [14]. Chromatographic separation for the nontargeted analysis was achieved using a Waters Acquity BEH C18 column (1.7 µm, 2.1 x 50 mm).

Interrogation of USDA soil database for mineral content:

Soil-mineralogy data were obtained via SQL query of the USDA Natural Resources Conservation Service (NRCS) National Cooperative Soil Survey (NCSS) Soil Characterization Database [15]. Soil data points lacking data characterizing soil horizon and/or soil taxa were excluded from analysis. Horizon sub-designations (e.g., B_g, B_t) of soil samples were consolidated into their general horizon designation, A, B, and C. Each soil sample within the curated dataset contained data describing its taxa and general soil horizon.

Using RStudio[16] with packages tidyverse[17] and readxl[18] in R (version 4.2.2), the dataset was subdivided to extract soil-sample data containing a given mineral of interest (e.g. quartz, Supplementary Table 1) within a given soil taxa of interest (e.g. alfisols, Supplementary Table 2). These data were further subdivided by soil horizon (A, B, or C) (Supplementary Table 1). This process yielded subsets of data that contained, for example, every A horizon alfisol with quartz concentration data. Further detail regarding soil sample mineral analysis methodology can be found in the Kellogg Soil Survey Laboratory Methods Manual [19]. The mean, median, standard deviation, and sample size were then determined for all combinations of soil taxa, horizon, and minerals of interest.

Supplementary Table 1**. Soil sample characteristic/analytes and corresponding NCSS database name.**

**Soil Sample Characteristic/Analyte**	**NCSS Database Name**
Soil Taxa	SSL_taxorder
Soil Horizon	hzn_desgn *(A, B, C)*
Quartz	QZ_Quartz_X_Ray
Vermiculite	VM_Vermiculite_Mica_X_Ray
Montmorillonite	MT_Montmorillonite_X_Ray
Kaolinite	KK_Kaolinite_X_Ray
Goethite	GE_Goethite_X_Ray
Hematite	HE_Hematite_X_Ray
Gibbsite	GI_Gibbsite_X_Ray
Hydrous Ferric Oxide (HFO)	fe_ammoniumoxalate_extractable
Hydrous Aluminum Oxide (HAO)	aluminum_ammonium_oxalate

Supplementary Table 2. **Studied soil orders.**

Entisols	Inceptisols	Mollisols	Alfisols
Spodosols	Ultisols	Oxisols	

Calculation of Fe and Al (oxy)hydroxide mineral(oid) electrostatic charge:

Ion sorption on Fe and Al (oxy)hydroxides generally is modeled as the product of two terms [20,21], an intrinsic sorption constant wherein an ion is chemically bound directly to the solid surface and a coulombic term representing nonspecific electrostatic sorption of ions in the diffuse layer as charge compensation for solid-surface charge. Characterization of intrinsic sorption constants requires experimental data for each solid-ion pair. For example, Chang et al. [22] report high-density occupation by PFAS of the Stern layer of fused silica beyond that determined solely by Boltzmann distribution. Regarding the coulombic term, the surface charge of Fe and Al (oxy)hydroxide mineral(oids) varies with pH and ionic strength (I), and comprises the electrostatic component of the composite electrochemical minimum potential. To calculate surface charge (σ) on these pH-dependent mineral(oid) surfaces, we employed the generalized two-layer model of Dzombak and Morel [20] and Karamalidis and Dzombak [21], drawing from the equations on Table 8.1, page 244 [20], according to the following:

Mass Law equations:

$$K_{a1s}^{app} = \frac{(-x^sOH^o)a_{H^+}}{(x^sOH_2^+)} = \frac{K_{a1}^{int}}{P} \qquad (x^sOH_2^+) = \frac{(-x^sOH^o)a_{H^+}P}{K_{a1}^{int}} \qquad (1,2)$$

$$K_{a2s}^{app} = \frac{(-x^sO^-)a_{H^+}}{(x^sOH^o)} = \frac{K_{a2}^{int}}{P} \qquad (x^sO^-) = \frac{K_{a2}^{int}(x^sOH^o)}{a_{H^+}P} \qquad (3,4)$$

$$K_{a1w}^{app} = \frac{(-x^wOH^o)a_{H^+}}{(x^wOH_2^+)} = \frac{K_{a1}^{int}}{P} \qquad (x^wOH_2^+) = \frac{(-x^wOH^o)a_{H^+}P}{K_{a1}^{int}} \qquad (5,6)$$

$$K_{a2w}^{app} = \frac{(-x^wO^-)a_{H^+}}{(x^wOH^o)} = \frac{K_{a2}^{int}}{P} \qquad (x^wO^-) = \frac{K_{a2}^{int}(x^wOH^o)}{a_{H^+}P} \qquad (7,8)$$

Mole Balance Equations:

$$TotH = (H^+) + (OH^-) + (-x^sOH_2^+) - (-x^sO^-) + (-x^wOH_2^+) - (-x^wOH_2^+) - (-x^wO^-) \qquad (9)$$

$$Tot(x^sOH) = (x^sOH_2^+) + (x^sOH^o) + (x^sO^-) \qquad (10)$$

Substituting Supplementary Equations 2 and 4 into Supplementary Equation 10:

$$Tot(x^{s}OH) = \frac{(-x^{s}OH^{o})a_{H^{+}P}}{K_{a1}^{int}} + (x^{s}OH^{o}) + \frac{K_{a2}^{int}(x^{s}OH^{o})}{a_{H^{+}}P}$$

$$Tot(x^{s}OH) = (-x^{s}OH^{o})\left[\frac{a_{H^{+}P}}{K_{a1}^{int}} + 1 + \frac{K_{a2}^{int}}{a_{H^{+}P}}\right] \quad (11)$$

Rearranging Supplementary Equation 11:

$$(-x^{s}OH^{o}) = \frac{Tot(x^{s}OH)}{\left[\frac{a_{H^{+}P}}{K_{a1}^{int}}+1+\frac{K_{a2}^{int}}{a_{H^{+}P}}\right]} \quad (12)$$

Analogously to Supplementary Equation 12 for weak sites:

$$(-x^{w}OH^{o}) = \frac{Tot(x^{w}OH)}{\left[\frac{a_{H^{+}P}}{K_{a1}^{int}}+1+\frac{K_{a2}^{int}}{a_{H^{+}P}}\right]} \quad (13)$$

Surface Charge Equations:

$$\sigma = \left[(-x^{s}OH_{2}^{+}) + (-x^{w}OH_{2}^{+}) - (-x^{s}O^{-}) - (-x^{w}O^{-})\right] \quad (14)$$

Substituting Supplementary Equations 2, 4, 6 and 8 into Supplementary Equation 14:

$$\sigma = \left[\left(\frac{(-x^{s}OH^{o})a_{H^{+}P}}{K_{a1}^{int}}\right) + \left(\frac{(-x^{w}OH^{o})a_{H^{+}P}}{K_{a1}^{int}}\right) - \left(\frac{K_{a2}^{int}(x^{s}OH^{o})}{a_{H^{+}P}}\right) - \left(\frac{K_{a2}^{int}(x^{w}OH^{o})}{a_{H^{+}P}}\right)\right]$$

$$\sigma = \left\{(-x^{s}OH^{o})\left[\left(\frac{a_{H^{+}P}}{K_{a1}^{int}}\right) - \left(\frac{K_{a2}^{int}}{a_{H^{+}P}}\right)\right] + (-x^{w}OH^{o})\left[\left(\frac{a_{H^{+}P}}{K_{a1}^{int}}\right) - \left(\frac{K_{a2}^{int}}{a_{H^{+}P}}\right)\right]\right\} \quad (15)$$

Substituting Supplementary Equations 12 and 13 into Supplementary Equation 15:

$$\sigma = \left\{\left[\frac{Tot(x^{s}OH)}{\left[\frac{a_{H^{+}P}}{K_{a1}^{int}}+1+\frac{K_{a2}^{int}}{a_{H^{+}P}}\right]}\right]\left[\left(\frac{a_{H^{+}P}}{K_{a1}^{int}}\right) - \left(\frac{K_{a2}^{int}}{a_{H^{+}P}}\right)\right] + \left[\frac{Tot(x^{w}OH)}{\left[\frac{a_{H^{+}P}}{K_{a1}^{int}}+1+\frac{K_{a2}^{int}}{a_{H^{+}P}}\right]}\right]\left[\left(\frac{a_{H^{+}P}}{K_{a1}^{int}}\right) - \left(\frac{K_{a2}^{int}}{a_{H^{+}P}}\right)\right]\right\}$$

$$\sigma = \left\{\left[\left(\frac{Tot(x^{s}OH)}{\left[\frac{a_{H^{+}P}}{K_{a1}^{int}}+1+\frac{K_{a2}^{int}}{a_{H^{+}P}}\right]}\right) + \left(\frac{Tot(x^{w}OH)}{\left[\frac{a_{H^{+}P}}{K_{a1}^{int}}+1+\frac{K_{a2}^{int}}{a_{H^{+}P}}\right]}\right)\right]\left[\left(\frac{a_{H^{+}P}}{K_{a1}^{int}}\right) - \left(\frac{K_{a2}^{int}}{a_{H^{+}P}}\right)\right]\right\} \quad (16)$$

Where Tot(x^sOH) is the total strong (complexation) protonation-deprotonation sites per mineral(oid) cation (Fe or Al) (mol/mol), Tot(x^wOH) is the total weak (electrostatic) protonation-deprotonation sites per mineral(oid) cation, a_{H+} is the bulk aqueous proton activity (i.e., 10^{-pH}), K_{a1}^{int} is the intrinsic equilibrium constant defining the chemical equilibrium component of potential energy for deprotonating a neutrally charged site (i.e., $xOH^0 \rightarrow xO^-$), K_{a2}^{int} is the intrinsic equilibrium constant defining the chemical equilibrium component of potential energy for deprotonating a positively charged site (i.e., $xOH_2^+ \rightarrow xO^0$), and P is the "coulombic correction factor".

Conceptually, P expresses the electrostatic equilibrium component of potential energy for specified pH and I in common form with chemical potential by the relation P=exp(-FΨ/RT) where F is Faraday's constant, Ψ is aqueous diffuse-layer potential (coulombs), R is the Ideal Gas constant, and T is absolute temperature. Dzombak and Morel have tabulated experimental values of log P for HFOs as a function of pH and I and we regressed these data against pH for selected values of I using a quadratic fit (Supplementary Table 3, Supplementary Fig. 2). Likewise, values of log P for gibbsite are tabulated in the mineral equilibrium model MINEQL+ V5.0 (Dave Dzombak, personal communication), which we summarized and have regressed for selected values of I using a quadratic fit (Supplementary Table 4, Supplementary Fig. 3). We approximated goethite log P with the quadratic regression of the HFO log P values.

For the bulk aqueous pH, we used measured values of soil pH, assuming the soil pH reflected equilibrium with long-term average soil-solution pH, likely buffered by a combination of H_2CO_3-HCO_3^- equilibrium generated by elevated subsurface P_{CO2} from oxidation of dissolved and colloidal natural organic matter percolating through the profile [23] with dissolution/precipitation of HAO and micro-crystalline gibbsite [24].

In the absence of a complete chemical profile for major-ion concentrations in bulk soil water, to approximate soil-solution ionic strength I, we used field-measured values of temperature and pH in shallow groundwater at the study sites. Specific conductance at 25 °C was calculated from measured specific conductance at field temperature (T) by [25]:

$$SC_{25} = SC_T[1 + 0.02(25 - T)] \tag{17}$$

Then I was approximated from SC_{25} according to [26]:

$$I\ (mM) = 0.016SC_{25} \tag{18}$$

Using the values in Supplementary Table 9, we conservatively used I=0.005 M in subsequent calculations.

For the amorphous and short-range ordered hydrous ferric oxides (HFOs), both strong and weak Tot(xOH) sites were included in the modeling [20] but for gibbsite, only weak sites were necessary [21], effectively setting the strong term in Supplementary Equation 16 to zero. For our efforts here, we use sorption parameters as tabulated in Supplementary Table 5, including a zero-point of charge (ZPC) for HFO of 8.11 [20], 9.18 for gibbsite [21] and 8.29 for goethite [27].

In the absence of sorption parameters for HAO, we note Nordstrom and Ball's report [24] that acidified natural waters commonly approximate Al^{3+}-H^{+} equilibrium in a narrow range bounded by the solubility of amorphous $Al(OH)_3$ and microcrystalline gibbsite, reflecting the similarity between these solids and the continuum in crystallinity. Based on this observation, we modeled HAO charge with the parameters Karamalidis and Dzombak [21] report for gibbsite, but corrected to the specific surface area reported for HAO by Gu and Karthikeyan [28]. All charges were normalized to dry bulk soil mass based upon analyzed mineral(oid) concentration.

Supplementary Table 3. **Tabulation of Log P for HFO from Dzombak and Morel's Table 8.8.**

pH	Ionic Strength				
	0.5	0.1	0.05	0.01	0.005
4.0	-1.85	-2.45	-2.68	-3.11	-3.27
4.2	-1.84	-2.41	-2.62	-3.01	-3.16
4.4	-1.81	-2.35	-2.54	-2.91	-3.05
4.6	-1.78	-2.28	-2.46	-2.80	-2.93
4.8	-1.74	-2.20	-2.37	-2.68	-2.80
5.0	-1.69	-2.12	-2.27	-2.56	-2.67
5.2	-1.63	-2.02	-2.16	-2.43	-2.53
5.4	-1.56	-1.92	-2.05	-2.29	-2.39
5.6	-1.48	-1.81	-1.93	-2.15	-2.24
5.8	-1.39	-1.69	-1.80	-2.01	-2.08
6.0	-1.29	-1.57	-1.67	-1.85	-1.92
6.2	-1.18	-1.44	-1.53	-1.70	-1.75
6.4	-1.07	-1.31	-1.39	-1.53	-1.58
6.6	-0.96	-1.17	-1.24	-1.36	-1.41
6.8	-0.84	-1.03	-1.09	-1.19	-1.23
7.0	-0.72	-0.88	-0.93	-1.02	-1.05
7.2	-0.60	-0.73	-0.77	-0.84	-0.86
7.4	-0.47	-0.57	-0.60	-0.65	-0.68
7.6	-0.34	-0.41	-0.44	-0.47	-0.49
7.8	-0.21	-0.25	-0.27	-0.29	-0.30
8.0	-0.07	-0.09	-0.09	-0.10	-0.11
8.2	0.06	0.07	0.08	0.09	0.08
8.4	0.19	0.24	0.25	0.28	0.27
8.6	0.33	0.40	0.42	0.46	0.46
8.8	0.46	0.56	0.59	0.65	0.65
9.0	0.58	0.71	0.76	0.83	0.84
9.2	0.71	0.87	0.92	1.01	1.02
9.4	0.83	1.01	1.08	1.18	1.21
9.6	0.95	1.16	1.23	1.36	1.39
9.8	1.06	1.30	1.38	1.52	1.56
10.0	1.17	1.43	1.52	1.69	1.73
10.2	1.28	1.56	1.66	1.85	1.90
10.4	1.38	1.68	1.79	2.00	2.06
10.6	1.47	1.80	1.92	2.15	2.22
10.8	1.55	1.91	2.04	2.29	2.37
11.0	1.62	2.01	2.15	2.42	2.51

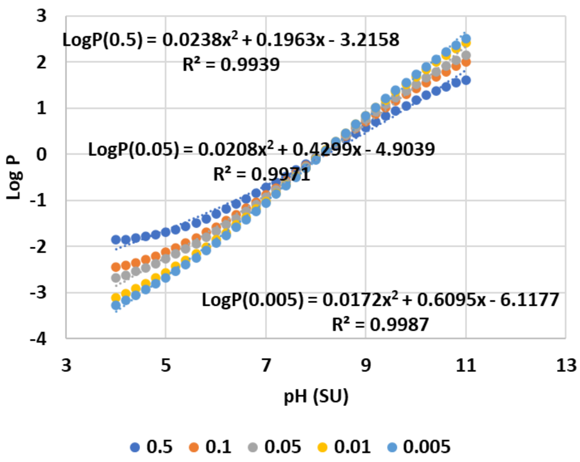

Supplementary Fig. 2. **Quadratic regression of Log P for HFO as a function of pH, at ionic strengths 0.5-0.005 M.**

Supplementary Table 4. **Tabulation of Log P for Gibbsite from MINEQL+ V5.0 (personal communication from Dave Dzombak).**

pH	Ionic Strength					
	**0.5**	**0.1**	**0.05**	**0.01**	**0.005**	**0.001**
**4.0**	-2.72	-3.12	-3.27	-3.58	-3.70	-3.96
**4.2**	-2.64	-3.02	-3.16	-3.46	-3.57	-3.83
**4.4**	-2.56	-2.92	-3.05	-3.34	-3.45	-3.70
**4.6**	-2.47	-2.81	-2.94	-3.21	-3.32	-3.57
**4.8**	-2.37	-2.69	-2.82	-3.09	-3.20	-3.44
**5.0**	-2.27	-2.58	-2.70	-2.96	-3.07	-3.31
**5.2**	-2.16	-2.46	-2.57	-2.83	-2.94	-3.18
**5.4**	-2.05	-2.34	-2.45	-2.70	-2.81	-3.05
**5.6**	-1.94	-2.21	-2.33	-2.58	-2.68	-2.91
**5.8**	-1.82	-2.09	-2.20	-2.45	-2.55	-2.77
**6.0**	-1.70	-1.96	-2.07	-2.31	-2.41	-2.64
**6.2**	-1.58	-1.84	-1.94	-2.18	-2.28	-2.50
**6.4**	-1.46	-1.71	-1.81	-2.05	-2.15	-2.35
**6.6**	-1.34	-1.58	-1.68	-1.91	-2.01	-2.21
**6.8**	-1.22	-1.45	-1.55	-1.78	-1.87	-2.06
**7.0**	-1.10	-1.33	-1.42	-1.64	-1.73	-1.90
**7.2**	-0.97	-1.20	-1.29	-1.50	-1.59	-1.75
**7.4**	-0.85	-1.07	-1.16	-1.36	-1.44	-1.58
**7.6**	-0.74	-0.94	-1.03	-1.22	-1.29	-1.42
**7.8**	-0.62	-0.82	-0.90	-1.07	-1.14	-1.25
**8.0**	-0.52	-0.69	-0.77	-0.92	-0.98	-1.07
**8.2**	-0.41	-0.57	-0.64	-0.77	-0.82	-0.90
**8.4**	-0.32	-0.45	-0.51	-0.62	-0.66	-0.72
**8.6**	-0.23	-0.33	-0.38	-0.46	-0.49	-0.53
**8.8**	-0.14	-0.22	-0.25	-0.30	-0.32	-0.35
**9.0**	-0.07	-0.10	-0.11	-0.14	-0.15	-0.16
**9.2**	0.01	0.01	0.02	0.02	0.02	0.02
**9.4**	0.08	0.13	0.15	0.18	0.19	0.21
**9.6**	0.16	0.24	0.28	0.34	0.36	0.39
**9.8**	0.25	0.36	0.41	0.50	0.53	0.58
**10.0**	0.34	0.48	0.54	0.66	0.70	0.76
**10.2**	0.44	0.60	0.67	0.81	0.86	0.94
**10.4**	0.54	0.72	0.80	0.96	1.02	1.12
**10.6**	0.65	0.85	0.93	1.11	1.18	1.29
**10.8**	0.77	0.98	1.06	1.26	1.33	1.46
**11.0**	0.88	1.10	1.20	1.40	1.48	1.63

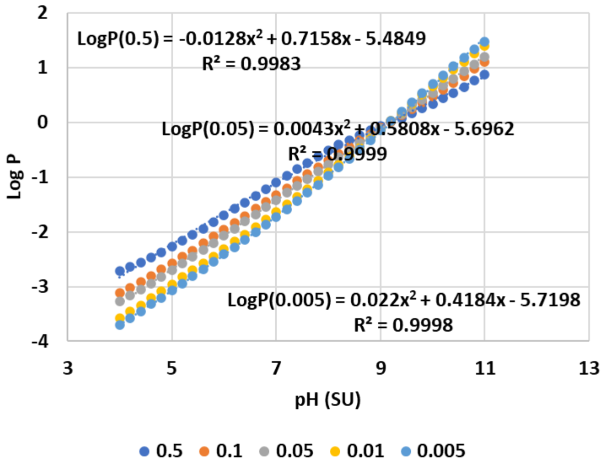

Supplementary Fig. 3. **Quadratic regression of Log P for gibbsite as a function of pH, at ionic strengths 0.5-0.005 M.**

Supplementary Table 5. **Properties to model mineral(oid) surface charge.**

Mineral(oid) Phase	Reference	Nominal Formula	$Log(K_{a1})$	$Log(K_{a2})$	Zero Point of Charge (1) (pH)	Density (g/cm^3)	Surface area (m^2/g)	Total surface site density ($sites/nm^2$)	Strong surface site density (mol/mol Fe or Al)	Weak surface site density (mol/mol Fe or Al)
**HFO**	**Dzombak and Morel, 1990**	$Fe(OH)_3$	-7.29	-8.93	8.11	3.50	600	2.31	0.005	0.200
**HAO**	**Karamalidis & Dzomdak, 2010 (2)**	$Al(OH)_3$	-7.17	-11.18	9.18	2.42		8	none	0.400 (3)
	**Gu and Karthikeyan, 2005**						386			
**Gibbsite**	**Karamalidis & Dzomdak, 2010**	$Al(OH)_3$	-7.17	-11.18	9.18	2.42	32	8	none	0.033 (3)
**Goethite**	**Mathur & Dzombak, 2006**	FeOOH	-6.93	-9.65	8.29	*3.50*	60	2	none	0.018 (3)
**Kaolinite Al facet**	**Kumar et al., 2017**	$Al_2Si_2O_5(OH)_4$			7.00			0.028	none	0.00025 (3)
	**Tombacz & Szekeres, 2006 (4)**						41.5			
	**Gibbsite $Log(K_a)$ + 2.18 (5)**		-4.99	-9.00						

(1) Zero point of charge (ZPC) defined as ZPC = -(log K_{a1}+log K_{a2})/2.
(2) HAO properties assumed equal to gibbsite, except weak surface site density is corrected to surface area of Gu and Karthikeyan (2005).
(3) Weak surface site density (WSSD; mol/mol) calculated from surface area (SA), formula molecular mass (FMM) and total surface site density (TSSD; sites/nm^2) according to WSSD = (SA*FMM*TSSD)/($6.02*10^5$).
(4) Reported kaolinite surface area is for Al facet only, taken as 1/2 value reported for full kaolinite surface area reported in Tombacz & Szerkeres [29].
(5) Based on Karamalidis & Dzombak [21] gibbsite ZPC reported as 9.18 & Kumar et al. [30] kaolinite ZPC as 7.00, 9.18 - 7.00 = 2.18, taken as Log K difference from gibbsite.

Table references: [20,21,27-30]

Calculation of kaolinite electrostatic charge:

Kaolinite is a phyllosilicate clay, with one sheet consisting of silica tetrahedra and a second sheet, consisting of aluminum octahedra, being isostructural with gibbsite. Foundational studies on the sorptive properties of kaolinite were based on bulk-phase characterization experiments, and the widely held conceptual model suggested a small permanent surface charge on the tetrahedral or both sheet faces arising from hetero-valent cation substitution along with pH-dependent charges dominantly on the broken-bond sheet edges [31]. In recent years, however, researchers have employed atomic-force microscopy (AFM) to characterize independently the Si-tetrahedral and the Al-octahedral planes of kaolinite [30,32]. These efforts have identified pH-dependent charge sites on both faces with a zero-point of charge (ZPC) falling roughly at ≤4.00 for the tetrahedral face [30,32] and 7.00 on the octahedral plane [30], making kaolinite a zwitterionic mineral in the pH range between these ZPCs. While the octahedral sheet of kaolinite is isostructural with gibbsite, this ZPC of 7.00 falls 2.18 pH units below that of gibbsite's ZPC of 9.18, likely due to charge neutralization by the negative charge imposed by kaolinite's adjacent

tetrahedral layer. Ahmad et al. [33] have studied sorption of PFAS on kaolinite at pH=7, the ZPC value identified for kaolinite by Kumar et al. [30].

Informed by the distribution of per- and polyfluoroalkyl substances (PFAS) at our study sites being correlated with kaolinite in our study, we modeled the pH-dependent charge of kaolinite's octahedral gibbsite layer using the kaolinite octahedral surface-site density of Kumar et al. [30] and by shifting the gibbsite sorption parameters of Karamalidis and Dzombak [21] the 2.18 pH units downward from gibbsite's ZPC=9.18 to kaolinite's ZPC=7.00 (Supplementary Table 6). This places our estimated kaolinite $\log(K_{a1})$=4.99 (Supplementary Table 5), quite close to Kumar's value of "pK_a near 5" [30], bolstering confidence regarding veracity of these approximations. With these ZPC and $\log(K_{a1})$ values assigned, $\log(K_{a2})$ is defined by the relationship $ZPC = (\log(K_{a1})+\log(K_{a2}))/2$. Kumar et al. [30] report a surface Al-facet site density more than 100-fold lower than that of isostructural gibbsite (Supplementary Table 5), a curious discrepancy. To our knowledge, this is the only Al-facet site-density value for kaolinite in the literature. To the extent this kaolinite site density understates actual site density, our absolute calculated values of kaolinite charge might be low as well. Nevertheless, the relative kaolinite charge values among samples should not be affected by this uncertainty.

Supplementary Table 6. **Tabulation of Log P for Kaolinite approximated from Gibbsite by Log pH($P_{kaolinite}$) = Log pH($P_{gibbsite}$) - 2.18.**

pH	Ionic Strength					
	**0.5**	**0.1**	**0.05**	**0.01**	**0.005**	**0.001**
**1.82**	-2.72	-3.12	-3.27	-3.58	-3.70	-3.96
**2.02**	-2.64	-3.02	-3.16	-3.46	-3.57	-3.83
**2.22**	-2.56	-2.92	-3.05	-3.34	-3.45	-3.70
**2.42**	-2.47	-2.81	-2.94	-3.21	-3.32	-3.57
**2.62**	-2.37	-2.69	-2.82	-3.09	-3.20	-3.44
**2.82**	-2.27	-2.58	-2.70	-2.96	-3.07	-3.31
**3.02**	-2.16	-2.46	-2.57	-2.83	-2.94	-3.18
**3.22**	-2.05	-2.34	-2.45	-2.70	-2.81	-3.05
**3.42**	-1.94	-2.21	-2.33	-2.58	-2.68	-2.91
**3.62**	-1.82	-2.09	-2.20	-2.45	-2.55	-2.77
**3.82**	-1.70	-1.96	-2.07	-2.31	-2.41	-2.64
**4.02**	-1.58	-1.84	-1.94	-2.18	-2.28	-2.50
**4.22**	-1.46	-1.71	-1.81	-2.05	-2.15	-2.35
**4.42**	-1.34	-1.58	-1.68	-1.91	-2.01	-2.21
**4.62**	-1.22	-1.45	-1.55	-1.78	-1.87	-2.06
**4.82**	-1.10	-1.33	-1.42	-1.64	-1.73	-1.90
**5.02**	-0.97	-1.20	-1.29	-1.50	-1.59	-1.75
**5.22**	-0.85	-1.07	-1.16	-1.36	-1.44	-1.58
**5.42**	-0.74	-0.94	-1.03	-1.22	-1.29	-1.42
**5.62**	-0.62	-0.82	-0.90	-1.07	-1.14	-1.25
**5.82**	-0.52	-0.69	-0.77	-0.92	-0.98	-1.07
**6.02**	-0.41	-0.57	-0.64	-0.77	-0.82	-0.90
**6.22**	-0.32	-0.45	-0.51	-0.62	-0.66	-0.72
**6.42**	-0.23	-0.33	-0.38	-0.46	-0.49	-0.53
**6.62**	-0.14	-0.22	-0.25	-0.30	-0.32	-0.35
**6.82**	-0.07	-0.10	-0.11	-0.14	-0.15	-0.16
**7.02**	0.01	0.01	0.02	0.02	0.02	0.02
**7.22**	0.08	0.13	0.15	0.18	0.19	0.21
**7.42**	0.16	0.24	0.28	0.34	0.36	0.39
**7.62**	0.25	0.36	0.41	0.50	0.53	0.58
**7.82**	0.34	0.48	0.54	0.66	0.70	0.76
**8.02**	0.44	0.60	0.67	0.81	0.86	0.94
**8.22**	0.54	0.72	0.80	0.96	1.02	1.12
**8.42**	0.65	0.85	0.93	1.11	1.18	1.29
**8.62**	0.77	0.98	1.06	1.26	1.33	1.46
**8.82**	0.88	1.10	1.20	1.40	1.48	1.63

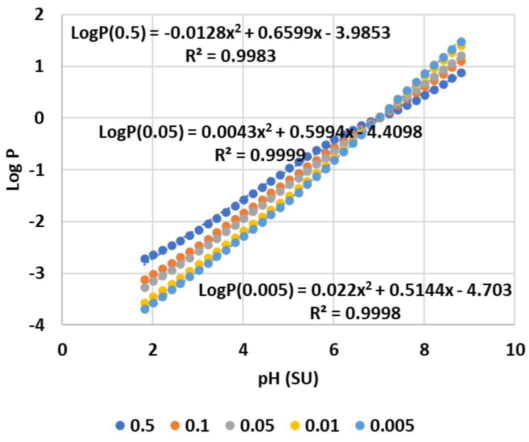

Supplementary Fig. 4. **Quadratic regression of Log P for kaolinite as a function of pH, at ionic strengths 0.5-0.005 M.**

Calculation of vadose moisture content and air-water interface:

Over the course of a number of papers, Brusseau and colleagues have developed models documenting how PFAS sorb at the air-water interface in the vadose zone [34-36]. In Brusseau [35], specific area of the air-water interface (A_{aw}; $cm^2/cm^3 = cm^{-1}$) was estimated by two independent methods.

Brusseau Equation 4 offers an estimate of A_{aw} (A_{awB}) using the Brunauer-Emmett-Teller specific surface area BET_v (nitrogen sorption specific surface area per cm^3 soil volume) and fractional saturation (F_s):

$$A_{awB} = [0.83(1 - F_S)^2 + 0.16(1 - F_S)][761(\log BET_v) - 2025] \tag{19}$$

We estimated the BET_v of our samples using our USDA soil texture (Supplementary Tables 7-8) and the mean mass-basis BET (BET_m) value reported for each soil texture reported in Arthur et al. [37] excepting 'sandy clay', which we estimated as the mean of values reported in Arthur et al. for the surrounding soil textures of 'clay' and 'sandy clay loam'. After expressing Arthur BET_m as cm^2/g, we estimated BET_v by multiplying BET_m by soil bulk densities (ρ_b; g/cm^3) reported for the Emporia loamy sand (Field 1) and Norfolk loamy sand (Field 2) as reported for each USDA soil series by depth interval [2].

Brusseau Equation 5 offers an estimate of A_{awd} using the median particle diameter (d_{50}) and fractional saturation (F_s):

$$A_{awd} = [-2.85(F_S) + 3.6)]\left[3.9(1 - F_S)({d_{50}}^{-1.2})\right] \tag{20}$$

We estimated the d_{50} of our samples using our USDA soil texture (Supplementary Tables 7-8) and the d_{50} value reported for each soil texture reported in Dosskey et al. [38].

Regarding F_s, field sampling took place in the late Carolina summer, during the week of September 13, 2021. Weather records at nearby Florence Regional Airport indicate that precipitation totaled 1 cm in the 20 days preceding initiation of sampling. Based on these records, we assumed that there was negligible water actively percolating through the sandy vadose profile and therefore the vertical distribution of water capillary pressure (P_c; cm_{H2O}) could be reasonably estimated by Laplacian capillary rise from the free-standing water table located at 3.66 m (366 cm) for Field 1 and 4.57 m (457 cm) for Field 2 below ground level. With this estimate of P_c and soil-textural data for each sample, to approximate F_s in the Brusseau A_{aw} equations we employed the Boltzmann Distribution equation and geometric-mean fitting parameters for every USDA textural class as reported in Bumb et al. (Bumb et al. 1992):

$$Ln\ F_s = \frac{(-P_c - P_{c1})}{\beta} \quad (21)$$

where the fitting parameters P_{c1} roughly equals to the capillary pressure at the Fs-Pc curve inflection point where maximum F_s is approached asymptotically and β varies as a function of pore-size distribution (Bumb et al. 1992).

Porosity (Po) was estimated from bulk density (ρ_b) reported for each soil series by depth interval (USDA 2023) and assigning a particle density (ρ_p) for quartz, 2.65 g/cm^3, as representative mineral density, by:

$$Po = \left(1 - {}^{\rho_b}/_{\rho_p}\right) \quad (22)$$

According to the USDA (USDA 2023), the "representative" bulk densities of Emporia loamy sand are 1.60 g/cm^3 (0-4 in), 1.64 g/cm^3 (4-21 in), 1.58 g/cm^3 (21-45 in) and 1.5 g/cm^3 (45-60 in). We assumed lesser eluvial accumulation at 60 in and this same 1.5 g/cm^3 for deeper intervals. According to the USDA (USDA 2023), the "representative" bulk densities of Norfolk loamy sand 1.68 g/cm^3 (0-7 in), 1.68 g/cm^3 (7-15 in), 1.60 g/cm3 (15-72 in). We assumed lesser eluvial accumulation at 72 in and this same 1.60 g/cm^3 for deeper intervals.

Transformations and filtering of variables for data analysis:

To scrutinize the data for relationships we first assessed variance for each variable by calculating coefficients of variation (CVs). When physically related variables (e.g., HAO concentration and HAO electrostatic charge) had CVs less than unity or had only one of the closely related variables slightly exceed unity, we did not transform these data. When the CV of a variable grossly exceeded unity, we log-transformed this variable and its physically related variable. With this screening, we log-transformed the PFAS data, the soil organic fractions, TOC and Total Nitrogen, and the extractable Mehlich metals, and left other variables untransformed. Independent variables that had ≥ 25% non-detect values were eliminated from all analyses, and any remaining non-detects were replaced with a randomized fraction times the lowest detected value for that variable.

Beyond assessing normality of data, the PFAS data varied between fields due to differences in sludge loading rate and timing of sludge applications (Supplementary Fig. 1). Considering our objectives included to assess possible relations of PFAS to geochemical parameters, this source of variation between fields presented the potential to obfuscate the relationships. To diminish this source of variation, each PFAS logged concentration was divided by the mean of each field's five surface soil logged

concentrations. With the combined transformations of log PFAS concentration followed by dividing by mean surface soil value, we found the ratios of the mean values, $\bar{x}_{Field\ 1}/\bar{x}_{Field\ 2}$, for C4 through C8 (PFBA – PFOA) fell at nearly unity (mean ratio for C4-C8 = 0.96, range = 0.92-0.97) indicating these transformations minimized variation in the data due to differences in sludge application history between the two sampled fields. Finally, a sampling artifact of the coring process using was observed at depths corresponding to the sampling core length, and the presumed contaminated interval was removed from subsequent correlations analysis: 4.0-4.5 ft, 8.0-9.0 ft, 4.0-4.5 ft, 8.0-9.0 ft, 12.0-14.0 ft, and 20.0-22.0 ft.

Then we calculated Pearson correlation coefficients among PFAS and geochemical data, and generated a clustering heat map, color coded by nominal significance levels of correlation, wherein quantitative significance of any single value remains uncertain, but larger clustering patterns in relative significance can guide data assessment. Pearson correlation coefficients were calculated at various truncated depths to describe correlations within soil zones, e.g., unsaturated and saturated media, and can be found in corresponding figure legends.

II. Data summary:

Supplementary Table 7. **Geochemical soil parameters for Colluvial Field 1 for hand-augured surface soils (SF) and cored soil bored (SB) samples.**

			X-ray (%)								Amorphous, mg/kg				%		%			
Sample ID- Depth Increment (ft)	Mid-depth (m)	Solids (%)	Gibbsite	Goethite	Illite	Kaolinite	Quartz	Vermiculite	Manganite	Rutile	27Al	55Mn	56Fe	pH	N	TOC	Sand	Silt	Clay	Soil Type
SF101-0.0-0.5	0.08	92	0.00	0.00	0.05	2.08	95.96	0.00	0.00	1.92	396.90	109.85	229.97	7.14	0.14	2.15	82.3	11.5	6.2	Loamy Sand
SF102-0.0-0.5	0.08	89	0.00	0.00	1.10	2.36	96.53	0.00	0.00	0.00	420.59	123.95	240.77	6.67	0.11	1.14	84.3	11.5	4.2	Loamy Sand
SF103-0.0-0.5	0.08	95	0.00	0.00	0.36	1.68	97.95	0.00	0.00	0.00	359.79	148.66	210.56	7.62	0.14	1.67	88.3	5.4	6.3	Sand
SF104-0.0-0.5	0.08	93	0.00	0.00	0.15	8.66	91.19	0.00	0.00	0.00	455.47	147.54	279.95	6.62	0.16	1.70	82.4	11.3	6.3	Loamy Sand
SB01-0.0-0.5	0.08	93	4.87	1.26	2.68	12.38	78.51	0.31	0.00	0.00	473.88	66.53	288.71	6.95	0.06	0.57	78.6	8.3	13.1	Sandy Loam
SB01-0.5-1.0	0.23	89	12.01	10.30	4.77	34.56	36.65	1.70	0.00	0.00	601.61	11.50	188.94	7.23	0.05	0.28	60.6	7.3	32.1	Sandy Clay Loam
SB01-1.0-1.5	0.38	89	14.08	10.94	5.46	43.11	25.51	0.90	0.00	0.00	594.42	0.82	157.50	7.50	0.03	0.20	58.6	7.3	34.1	Sandy Clay Loam
SB01-1.5-2.0	0.53	88	11.80	10.06	4.37	47.20	25.71	0.88	0.00	0.00	455.71	0.84	99.87	7.16	0.03	0.08	59.6	7.3	33.1	Sandy Clay Loam
SB01-2.0-2.5	0.69	86	9.24	14.76	5.05	43.05	27.33	0.58	0.00	0.00	416.66	2.43	89.91	4.84	0.03	0.10	61.6	6.2	32.2	Sandy Clay Loam
SB01-2.5-3.0	0.84	85	12.57	9.76	9.08	47.49	20.32	0.78	0.00	0.00	581.90	0.67	123.42	4.78	0.01	0.10	61.3	6.3	32.4	Sandy Clay Loam
SB01-3.0-3.5	0.99	86	12.17	16.62	5.46	42.55	23.02	0.17	0.00	0.00	467.66	0.71	87.24	4.74	0.02	0.05	61.6	7.1	31.2	Sandy Clay Loam
SB01-3.5-4.0	1.14	86	11.01	19.30	8.23	43.68	16.35	0.18	0.00	1.24	481.61	8.93	138.09	4.69	0.03	0.06	61.6	6.1	32.2	Sandy Clay Loam
SB01-4.0-4.5	1.30	87	12.39	4.47	9.40	49.88	23.58	0.29	0.00	0.00	460.82	9.26	149.54	4.75	0.03	0.13	63.1	7.8	29.1	Sandy Clay Loam
SB01-4.5-5.0	1.45	84	15.27	1.22	11.73	54.89	16.89	0.00	0.00	0.00	475.80	1.19	26.71	4.59	0.01	0.04	57.2	7.7	35.1	Sandy Clay
SB01-5.0-6.0	1.68	83	15.04	15.86	10.31	47.50	11.29	0.00	0.00	0.00	450.71	0.53	49.79	4.64	0.02	0.07	54.3	7.4	38.4	Sandy Clay
SB01-6.0-7.0	1.98	82	12.76	3.92	12.15	57.96	13.20	0.00	0.00	0.00	565.48	0.55	55.32	4.53	0.04	0.05	56.1	9.4	34.6	Sandy Clay Loam
SB01-7.0-8.0	2.29	82	9.05	0.00	13.48	58.16	19.31	0.00	0.00	0.00	494.41	0.45	45.34	4.45	0.02	0.05	60.1	7.4	32.6	Sandy Clay Loam
SB01-8.0-9.0	2.59	80	15.17	2.35	10.21	55.15	17.12	0.00	0.00	0.00	389.71	0.28	56.32	4.58	0.02	0.05	62.1	7.3	30.6	Sandy Clay Loam
SB01-9.0-10.0	2.90	80	13.23	1.62	12.22	52.76	20.17	0.01	0.00	0.00	397.65	0.35	50.94	4.44	0.02	0.05	66.1	7.3	26.6	Sandy Clay Loam
SB01-10.0-12.0	3.35	82	16.72	0.20	12.44	49.34	21.30	0.00	0.00	0.00	368.64	0.42	137.46	4.50	0.02	0.07	61.6	9.3	29.1	Sandy Clay Loam
SB01-12.0-16.0	4.27	85	8.47	0.00	11.89	37.30	42.34	0.00	0.00	0.00	115.46	0.65	67.50	4.73	0.02	0.04	83.3	4.4	12.4	Loamy Sand
SB01-16.0-18.0	5.18	75	4.87	1.34	15.59	40.53	35.36	0.00	0.00	2.30	97.07	0.49	13.61	4.58	0.02	0.04	89.6	2.2	8.2	Sand
SB01-18.0-20.0	5.79	76	2.60	0.05	6.74	12.69	77.92	0.02	0.00	0.00	168.16	0.80	37.61	4.64	0.03	0.04	82.6	5.2	12.2	Loamy Sand
SB01-20.0-22.0	6.40	74	5.94	1.21	13.82	14.86	64.18	0.00	0.00	0.17	131.47	0.97	89.86	4.67	0.02	0.04	90.6	2.1	7.2	Sand
SB01-22.0-24.0	7.01	77	0.80	0.51	6.07	12.49	79.08	0.00	0.00	1.05	145.90	0.77	37.07	4.67	0.01	0.03	90.6	2.1	7.2	Sand
SB01-24.0-26.0	7.62	79	5.13	1.23	9.24	27.11	57.29	0.00	0.00	0.00	107.65	1.12	66.00	4.56	0.02	0.04	86.6	4.1	9.2	Loamy Sand
SB01-26.0-28.0	8.23	77	3.16	1.13	4.97	20.12	70.63	0.00	0.00	0.00	195.87	0.74	27.81	4.65	0.01	0.03	85.6	4.1	10.2	Loamy Sand
SB01-28.0-30.0	8.84	81	2.89	6.24	7.41	23.47	57.11	0.00	2.88	0.00	89.17	0.59	20.49	4.64	0.02	0.03	92.6	1.1	6.2	Sand
SB01-30.0-32.0	9.45	81	3.95	3.12	8.79	12.41	71.76	0.00	0.00	0.00	41.81	0.72	23.48	4.77	0.02	0.04	95.7	0.1	4.2	Sand
SB01-32.0-34.0	10.06	73	0.22	0.32	5.11	5.92	87.21	0.11	0.00	1.58	69.24	0.95	63.93	4.73	0.02	0.04	95.7	0.1	4.2	Sand
SB01-34.0-36.0	10.67	81	0.00	0.01	0.64	18.45	80.90	0.00	0.00	0.00	58.70	1.04	53.12	4.81	0.01	0.02	95.7	0.1	4.2	Sand
SB01-36.0-38.0	11.28	75	0.00	0.60	5.31	6.12	87.97	0.00	0.00	0.00	73.35	1.25	65.70	4.79	0.02	0.02	96.1	0.0	3.9	Sand
SB01-38.0-40.0	11.89	80	0.03	0.01	0.10	0.50	99.36	0.00	0.00	0.00	40.79	0.94	7.57	4.88	0.02	0.02	98.1	0.0	1.9	Sand
SB01-40.0-42.0	12.50	76	0.12	0.04	1.91	0.79	97.14	0.00	0.00	0.00	32.00	0.90	8.02	4.80	0.02	0.02	97.2	0.0	2.8	Sand
SB01-42.0-44.0	13.11	76	2.34	1.11	1.33	3.05	92.18	0.00	0.00	0.00	30.85	0.91	12.46	4.83	0.02	0.02	97.2	0.0	2.8	Sand
SB01-44.0-46.0	13.72	80	0.11	1.20	2.70	4.65	91.34	0.00	0.00	0.00	28.91	1.05	9.56	4.87	0.06	0.02	98.2	0.0	1.8	Sand
SB01-46.0-48.0	14.33	82	0.00	1.45	0.95	0.63	95.38	0.00	0.00	1.58	48.08	0.33	25.87	4.96	0.01	0.02	98.4	0.0	1.6	Sand
SB01-48.0-50.0	14.94	78	3.89	0.47	2.19	1.91	88.38	1.65	0.00	0.00	33.68	0.35	22.36	4.88	0.02	0.03	98.2	0.0	1.8	Sand
SB01-50.0-52.0	15.54	81	10.89	0.00	8.10	5.02	73.40	0.00	1.09	1.49	33.68	0.27	18.28	4.82	0.01	0.03	98.1	0.0	1.9	Sand
SB01-52.0-56.0	16.46	79	4.38	0.00	4.09	10.65	80.88	0.00	0.00	0.00	31.45	0.37	27.92	4.86	0.01	0.03	98.1	0.0	1.9	Sand
SB01-56.0-58.0	17.37	73	0.00	0.04	3.10	2.81	94.05	0.00	0.00	0.00	46.16	0.29	40.27	4.77	0.01	0.02	97.1	0.0	2.9	Sand
SB01-58.0-60.0	17.98	77	0.00	0.00	4.24	3.86	91.90	0.00	0.00	0.00	53.42	0.28	28.37	4.77	0.02	0.02	97.1	0.0	2.9	Sand
SB01-60.0-64.0	18.90	81	0.39	12.38	1.95	4.53	93.51	4.40	0.00	0.00	31.17	0.28	18.83	4.85	0.02	0.07	98.0	0.0	2.0	Sand

Supplementary Table 7. **Geochemical soil parameters for Colluvial Field 1 (continued) for hand-augured surface soils (SF) and cored soil bored (SB) samples.**

Sample ID	Mid-depth	Mehlich 1 (mg/kg)														Soluble Anions (mg/kg)					ppm $CaCO_3$/pH		CEC	Base Saturation
Depth Increment (ft)	(m)	Ca	Cd	Cr	Cu	Fe	K	Mg	Mn	Mo	Na	Ni	P	Pb	Zn	Cl	F	NO_3	PO_4	SO_4	LBC	LBC_{eq}	meq/ 100 g	(% of CEC)
SF101-0.0-0.5	0.08	1990.07	0.27	0.20	0.43	2.25	20.92	81.78	21.28	<0.04	12.41	0.44	43.97	0.17	30.24	<2.55	<0.85	7.14	<12.77	<10.6	331.00	959.90	10.74	100.00
SF102-0.0-0.5	0.08	1521.37	0.06	<0.04	0.35	3.03	14.34	43.22	16.92	<0.04	12.23	0.34	47.07	0.24	9.99	2.59	<0.84	11.29	<12.61	<10.5	275.00	797.50	8.58	93.87
SF103-0.0-0.5	0.08	2734.53	0.06	0.09	<0.19	3.55	23.06	137.16	25.92	<0.04	22.57	0.64	72.77	<0.11	13.06	<2.40	<0.80	4.52	<11.98	<10.0	250.00	725.00	14.97	100.00
SF104-0.0-0.5	0.08	1587.67	0.08	<0.04	0.37	3.76	25.02	62.65	19.60	<0.04	12.53	0.41	40.11	0.23	22.43	<2.65	<0.89	8.77	<13.27	<11.1	388.00	1125.20	9.43	90.94
SB01-0.0-0.5	0.08	820.02	<0.04	0.04	0.31	3.12	11.15	35.14	9.72	<0.04	7.11	0.17	8.56	0.28	6.77	0.27	0.95	0.39	<0.35	0.5	214.00	597.32	4.51	98.68
SB01-0.5-1.0	0.23	721.01	<0.04	<0.04	<0.22	2.94	8.01	24.60	1.43	<0.04	11.69	<0.04	0.36	0.58	0.36	0.22	1.11	0.08	<0.35	1.0	356.00	1032.40	3.88	100.00
SB01-1.0-1.5	0.38	656.20	<0.05	<0.05	<0.24	2.77	6.77	30.31	<0.24	<0.05	16.46	<0.05	0.32	0.48	<0.24	0.17	1.20	<0.07	<0.35	1.9	482.00	1397.80	3.62	100.00
SB01-1.5-2.0	0.53	472.87	<0.05	<0.05	<0.23	2.53	8.40	70.17	<0.23	<0.05	30.47	<0.05	0.25	0.45	<0.23	0.15	<0.90	<0.07	<0.35	3.9	376.00	1090.40	3.10	100.00
SB01-2.0-2.5	0.69	216.09	<0.04	<0.04	<0.22	1.46	7.27	60.98	<0.22	<0.04	54.26	<0.04	0.23	0.54	<0.22	0.17	<0.91	<0.07	<0.35	6.8	1003.00	2908.70	14.41	12.79
SB01-2.5-3.0	0.84	114.59	<0.04	<0.04	<0.22	1.81	7.59	38.15	<0.22	<0.04	106.69	<0.04	0.17	0.62	<0.22	0.20	<0.92	<0.07	<0.35	7.4	836.00	2424.40	12.14	11.32
SB01-3.0-3.5	0.99	49.75	<0.04	<0.04	<0.22	2.18	7.97	20.57	<0.22	<0.04	111.87	<0.04	0.13	0.64	<0.22	0.21	<0.93	<0.07	<0.35	6.5	992.00	2876.80	13.93	6.65
SB01-3.5-4.0	1.14	35.00	<0.04	<0.04	<0.22	1.84	8.27	15.79	<0.22	<0.04	95.39	<0.04	0.16	0.61	<0.22	0.32	<0.92	<0.07	<0.35	4.5	1188.00	3445.20	16.66	4.46
SB01-4.0-4.5	1.30	169.75	<0.04	0.05	0.31	5.13	10.26	20.61	1.93	<0.04	43.19	0.07	0.62	0.57	0.79	0.88	<0.90	<0.07	<0.35	1.5	780.00	2262.00	11.41	10.82
SB01-4.5-5.0	1.45	28.64	<0.05	<0.05	<0.24	1.55	11.63	13.71	<0.24	<0.05	29.86	<0.05	0.22	0.63	<0.24	2.31	<0.97	<0.07	<0.35	<0.3	877.00	2543.30	12.68	3.29
SB01-5.0-6.0	1.68	21.27	<0.05	<0.05	<0.25	2.18	14.50	11.99	<0.25	<0.05	27.13	<0.05	<0.10	0.65	<0.25	2.62	<1.01	<0.07	<0.35	<0.3	734.00	2128.60	10.41	3.47
SB01-6.0-7.0	1.98	18.49	<0.04	<0.04	<0.22	2.03	15.16	10.55	<0.22	<0.04	25.60	<0.04	0.11	0.63	<0.22	2.93	<0.98	<0.07	<0.35	<0.3	1111.00	3221.90	16.25	2.03
SB01-7.0-8.0	2.29	18.84	<0.05	<0.05	<0.25	1.84	15.69	8.99	<0.25	<0.05	24.43	<0.05	0.22	0.64	<0.25	26.96	<0.97	<1.65	<14.60	<12.2	746.00	2163.40	11.35	2.78
SB01-8.0-9.0	2.59	17.79	<0.05	<0.05	<0.23	2.19	13.22	6.99	<0.23	<0.05	19.97	<0.05	0.24	0.65	<0.23	22.35	<0.95	<1.61	<14.18	<11.8	716.00	2076.40	10.32	2.60
SB01-9.0-10.0	2.90	15.60	<0.05	<0.05	<0.23	2.10	13.14	6.17	<0.23	<0.05	18.12	<0.05	0.25	0.55	<0.23	21.82	<0.96	<1.63	<14.42	<12.0	687.00	1992.30	10.44	2.32
SB01-10.0-12.0	3.35	48.57	<0.04	0.05	<0.22	3.51	13.36	12.35	<0.22	<0.04	18.19	<0.04	0.26	0.68	<0.22	1.92	<0.88	<0.07	<0.35	<0.3	909.00	2636.10	13.64	3.37
SB01-12.0-16.0	4.27	16.89	<0.04	<0.04	<0.20	2.31	6.07	6.34	<0.20	<0.04	8.10	<0.04	0.16	0.24	0.35	0.99	<0.77	0.08	<0.35	<0.3	193.00	520.23	2.55	7.38
SB01-16.0-18.0	5.18	14.47	<0.04	<0.04	0.24	1.16	5.46	6.88	<0.19	<0.04	8.76	<0.04	0.15	0.20	<0.19	1.29	<0.76	0.16	<0.35	<0.3	276.00	800.40	4.06	4.48
SB01-18.0-20.0	5.79	17.62	<0.04	<0.04	<0.20	0.50	7.39	8.92	<0.20	<0.04	10.60	<0.04	0.14	0.34	<0.20	1.50	<0.81	0.25	<0.35	<0.3	366.00	1061.40	5.24	4.34
SB01-20.0-22.0	6.40	18.53	<0.04	0.08	0.19	17.74	5.21	7.59	0.47	<0.04	10.53	0.06	0.17	0.24	0.52	1.48	<0.78	<0.07	<0.35	<0.3	188.00	501.88	2.55	8.42
SB01-22.0-24.0	7.01	12.19	<0.04	<0.04	<0.18	1.84	5.38	6.29	<0.18	<0.04	7.76	<0.04	0.16	0.34	0.22	1.32	<0.78	0.21	<0.35	<0.3	253.00	733.70	3.58	4.49
SB01-24.0-26.0	7.62	14.52	<0.03	<0.03	<0.17	0.59	6.90	7.93	<0.17	<0.03	7.82	<0.03	0.19	0.25	<0.17	1.21	<0.77	0.31	<0.35	<0.3	381.00	1104.90	5.58	3.41
SB01-26.0-28.0	8.23	15.98	<0.04	<0.04	<0.20	0.59	7.23	10.30	<0.20	<0.04	9.02	<0.04	0.19	0.32	<0.20	1.37	<0.79	0.35	<0.35	<0.3	383.00	1110.70	5.44	4.11
SB01-28.0-30.0	8.84	13.05	<0.04	<0.04	<0.18	0.85	3.91	5.99	<0.18	<0.04	6.67	<0.04	0.19	0.19	<0.18	0.89	<0.76	0.23	<0.35	<0.3	242.00	700.11	3.46	4.46
SB01-30.0-32.0	9.45	12.17	<0.03	<0.03	<0.17	0.73	2.70	4.01	<0.17	<0.03	6.12	<0.03	0.14	0.13	0.36	0.77	<0.72	0.21	<0.35	<0.3	49.00	0.00	0.13	129.23
SB01-32.0-34.0	10.06	15.80	<0.04	0.07	0.30	7.66	3.84	7.70	0.23	<0.04	8.31	0.06	0.20	0.15	0.24	0.87	<0.78	0.35	<0.35	<0.3	55.00	13.65	0.25	75.32
SB01-34.0-36.0	10.67	15.14	<0.04	<0.04	<0.19	0.63	3.18	7.19	<0.19	<0.04	7.49	<0.04	0.19	0.15	<0.19	0.69	<0.78	0.25	<0.35	<0.3	48.00	0.00	0.18	129.92
SB01-36.0-38.0	11.28	13.82	<0.04	<0.04	<0.19	0.47	<1.86	6.84	<0.19	<0.04	6.22	<0.04	0.21	0.12	<0.19	0.61	<0.78	0.32	<0.35	<0.3	48.00	0.00	0.16	134.23
SB01-38.0-40.0	11.89	12.99	<0.04	<0.04	<0.19	<0.38	1.89	5.64	<0.19	<0.04	5.32	<0.04	0.16	<0.11	<0.19	0.63	<0.79	0.33	<0.35	<0.3	42.00	0.00	0.14	203.25
SB01-40.0-42.0	12.50	14.66	<0.04	<0.04	<0.20	1.42	2.85	7.84	<0.20	<0.04	7.37	<0.04	0.25	0.13	0.21	0.61	<0.77	0.37	<0.35	<0.3	47.00	0.00	0.18	138.86
SB01-42.0-44.0	13.11	12.99	<0.04	<0.04	<0.20	0.42	1.95	7.03	<0.20	<0.04	5.81	<0.04	0.22	0.74	<0.20	0.59	<0.74	0.36	<0.35	<0.3	45.00	0.00	0.15	165.09
SB01-44.0-46.0	13.72	13.44	<0.04	<0.04	<0.19	1.09	<1.92	5.95	<0.19	<0.04	6.32	<0.04	0.22	0.15	<0.19	0.48	<0.70	0.31	<0.35	<0.3	40.00	0.00	0.15	220.21
SB01-46.0-48.0	14.33	11.88	<0.04	<0.04	<0.19	0.74	<1.94	5.13	<0.19	<0.04	5.16	<0.04	0.18	0.16	<0.19	0.41	<0.73	0.18	<0.35	<0.3	42.00	0.00	0.13	209.41
SB01-48.0-50.0	14.94	12.17	<0.04	<0.04	<0.18	0.68	<1.85	5.31	<0.18	<0.04	6.34	<0.04	0.24	0.13	<0.18	0.43	<0.72	0.30	<0.35	<0.3	37.00	0.00	0.14	264.61
SB01-50.0-52.0	15.54	11.99	<0.03	<0.03	<0.17	0.82	2.19	5.25	<0.17	<0.03	6.00	<0.03	0.24	0.12	<0.17	0.51	<0.70	0.35	<0.35	<0.3	37.00	0.00	0.14	268.75
SB01-52.0-56.0	16.46	24.02	<0.04	<0.04	<0.18	1.76	4.04	7.90	0.49	<0.04	7.79	<0.04	1.08	<0.11	0.26	0.51	<0.72	0.31	<0.35	<0.3	44.00	0.00	0.23	149.70
SB01-56.0-58.0	17.37	14.10	<0.04	<0.04	<0.21	0.55	2.61	6.73	<0.21	<0.04	6.22	<0.04	0.25	0.14	<0.21	0.66	<0.77	0.45	<0.35	<0.3	51.00	0.00	0.16	102.88
SB01-58.0-60.0	17.98	11.39	<0.04	<0.04	<0.18	0.44	2.33	5.65	<0.18	<0.04	5.34	<0.04	0.24	<0.11	<0.18	0.61	<0.73	0.41	<0.35	<0.3	48.00	0.00	0.13	140.32
SB01-60.0-64.0	18.90	12.41	<0.04	<0.04	<0.18	1.10	2.03	5.22	<0.18	<0.04	5.66	<0.04	0.28	<0.11	<0.18	0.55	<0.73	0.34	<0.35	<0.3	39.00	0.00	0.14	243.24

Supplementary Table 8. **Geochemical soil parameters Residual Field 2 for hand-augured surface soils (SF) and cored soil bored (SB) samples.**

			X-ray (%)								Amorphous, mg/kg				%		%			
**Sample ID- Depth Increment (ft)**	**Mid-depth (m)**	**Solids (%)**	**Gibbsite**	**Goethite**	**Illite**	**Kaolinite**	**Quartz**	**Vermiculite**	**Manganite**	**Rutile**	**27Al**	**55Mn**	**56Fe**	**pH**	**N**	**TOC**	**Sand**	**Silt**	**Clay**	**Soil Type**
**SF201-0.0-0.5**	0.08	93	0.00	0.00	0.07	1.22	97.21	0.00	0.00	1.50	357.39	30.64	226.78	5.08	0.08	0.63	76.6	15.2	8.3	Sandy Loam
**SF202-0.0-0.5**	0.08	92	3.97	0.01	1.70	3.07	91.25	0.00	0.00	0.00	325.87	44.35	160.53	5.85	0.11	0.60	72.6	21.1	6.3	Sandy Loam
**SF203-0.0-0.5**	0.08	92	0.00	0.00	0.36	1.54	98.10	0.00	0.00	0.00	340.69	42.59	182.59	5.86	0.08	0.80	74.6	19.2	6.2	Sandy Loam
**SF204-0.0-0.5**	0.08	91	2.51	0.00	0.54	10.32	84.37	0.00	0.00	2.27	398.97	69.59	238.78	5.70	0.12	1.30	74.6	19.2	6.2	Sandy Loam
**SB02-0.0-0.5**	0.08	93	0.04	0.05	0.01	2.59	94.32	0.00	1.24	1.85	344.20	27.23	186.39	5.67	0.06	0.53	74.6	19.3	6.1	Sandy Loam
**SB02-0.5-1.0**	0.23	94	3.06	0.04	2.61	4.27	90.02	0.00	0.00	0.00	372.27	29.03	139.34	6.15	0.03	0.31	74.1	17.3	8.6	Sandy Loam
**SB02-1.0-1.5**	0.38	93	4.25	0.08	2.06	9.90	83.46	0.25	0.00	0.03	725.96	32.50	238.05	5.75	0.05	0.40	66.0	18.1	15.9	Sandy Loam
**SB02-1.5-2.0**	0.53	89	8.51	0.16	3.92	22.34	63.67	1.41	0.00	0.00	731.38	1.76	148.01	5.04	0.03	0.17	55.9	16.3	27.8	Sandy Clay Loam
**SB02-2.0-2.5**	0.69	88	11.93	0.73	5.74	26.90	52.39	2.31	0.00	0.00	860.22	0.42	188.24	4.90	0.04	0.19	51.9	16.4	31.7	Sandy Clay Loam
**SB02-2.5-3.0**	0.84	87	9.94	0.25	4.59	27.17	56.26	1.78	0.00	0.00	852.24	0.52	209.79	4.76	0.02	0.10	52.0	16.3	31.7	Sandy Clay Loam
**SB02-3.0-3.5**	0.99	88	9.60	0.78	5.25	27.74	54.88	1.76	0.00	0.00	702.02	0.56	154.20	4.70	0.03	0.09	52.2	16.0	31.8	Sandy Clay Loam
**SB02-3.5-4.0**	1.14	87	10.35	1.27	3.47	33.23	49.98	1.69	0.00	0.00	761.52	0.39	155.60	4.76	0.02	0.09	50.6	15.3	34.1	Sandy Clay Loam
**SB02-4.0-4.5**	1.30	86	12.08	5.09	5.53	35.63	40.08	1.58	0.00	0.00	829.48	2.73	268.54	4.70	0.03	0.13	46.1	13.9	40.0	Sandy Clay
**SB02-4.5-5.0**	1.45	85	10.13	6.35	5.41	35.31	41.54	1.27	0.00	0.00	705.32	0.37	145.05	4.58	0.03	0.10	44.1	13.9	42.0	Clay
**SB02-5.0-6.0**	1.68	87	12.10	1.46	4.79	30.36	50.33	0.96	0.00	0.00	702.86	0.52	88.68	4.70	0.03	0.08	54.2	13.7	32.2	Sandy Clay Loam
**SB02-6.0-7.0**	1.98	88	6.34	0.01	3.18	28.68	60.93	0.86	0.00	0.00	389.32	0.37	43.31	4.62	0.03	0.04	66.0	13.9	20.1	Sandy Clay Loam
**SB02-7.0-8.0**	2.29	87	9.90	0.85	7.06	29.41	51.81	0.96	0.00	0.00	526.97	0.87	53.26	4.55	0.03	0.05	54.0	13.9	32.1	Sandy Clay Loam
**SB02-8.0-9.0**	2.59	85	11.11	0.16	5.78	37.19	44.79	0.97	0.00	0.00	553.34	0.50	50.50	4.41	0.04	0.05	60.0	9.8	30.1	Sandy Clay Loam
**SB02-9.0-10.0**	2.90	85	5.55	2.26	6.86	30.22	54.92	0.20	0.00	0.00	431.53	0.37	53.01	4.52	0.05	0.14	64.2	7.7	28.1	Sandy Clay Loam
**SB02-10.0-12.0**	3.35	79	8.73	0.01	11.87	34.12	45.01	0.27	0.00	0.00	582.68	0.49	82.87	4.42	0.04	0.06	51.9	12.3	35.8	Sandy Clay
**SB02-12.0-14.0**	3.96	78	2.45	0.00	10.18	30.87	56.31	0.18	0.00	0.00	445.60	2.83	257.75	4.58	0.03	0.06	61.7	12.5	25.8	Sandy Clay Loam
**SB02-14.0-16.0**	4.57	77	3.19	0.02	9.42	21.81	63.66	0.00	0.00	1.93	332.55	0.37	46.87	4.53	0.03	0.04	59.7	16.5	23.8	Sandy Clay Loam
**SB02-16.0-18.0**	5.18	71	12.04	0.00	8.94	33.59	45.43	0.00	0.00	0.00	460.22	0.77	81.97	4.50	0.02	0.05	49.7	20.5	29.8	Sandy Clay Loam
**SB02-18.0-20.0**	5.79	74	4.60	0.00	8.21	24.92	58.52	0.00	0.00	0.00	377.84	0.36	34.57	4.44	0.02	0.08	53.9	17.2	28.9	Sandy Clay Loam
**SB02-20.0-22.0**	6.40	71	7.69	0.00	8.44	20.17	61.67	0.03	0.00	2.02	278.03	0.36	30.66	4.56	0.02	0.04	70.0	10.1	19.9	Sandy Loam
**SB02-22.0-24.0**	7.01	73	0.89	0.00	11.58	21.71	62.20	0.01	0.00	3.61	265.42	0.48	30.58	4.48	0.02	0.03	73.0	9.1	17.9	Sandy Loam
**SB02-24.0-26.0**	7.62	72	4.63	0.00	8.00	31.21	52.24	0.00	0.00	3.92	277.59	0.70	37.25	4.54	0.03	0.05	68.1	12.1	19.8	Sandy Loam
**SB02-26.0-28.0**	8.23	71	5.97	1.56	9.91	25.59	52.02	0.00	0.00	4.95	221.63	1.73	58.69	4.56	0.03	0.04	68.2	10.1	21.7	Sandy Clay Loam
**SB02-28.0-30.0**	8.84	74	5.68	4.40	8.60	25.90	50.22	0.00	0.00	5.21	365.12	10.09	131.99	4.68	0.03	0.04	60.2	12.0	27.8	Sandy Clay Loam
**SB02-30.0-32.0**	9.45	76	12.43	0.00	10.97	26.38	46.10	0.00	0.00	4.12	263.12	356.21	223.23	4.47	0.02	0.04	72.4	7.6	19.9	Sandy Loam
**SB02-32.0-34.0**	10.06	78	11.69	0.00	10.69	26.30	49.32	0.00	0.00	2.00	205.36	127.14	74.27	4.59	0.02	0.03	76.2	7.8	16.0	Sandy Loam
**SB02-34.0-36.0**	10.67	73	9.91	0.00	11.72	26.72	49.69	0.00	0.00	1.96	163.48	0.87	37.90	4.43	0.02	0.03	76.2	7.8	16.0	Sandy Loam
**SB02-36.0-38.0**	11.28	79	4.00	0.00	7.33	14.96	70.71	0.00	0.00	3.01	80.74	0.98	55.82	4.74	0.02	0.04	93.2	1.8	5.0	Sand
**SB02-38.0-40.0**	11.89	75	13.37	0.00	10.19	38.89	36.28	0.00	0.00	1.28	407.80	2.15	143.53	4.39	0.02	0.04	55.0	12.9	32.0	Sandy Clay Loam
**SB02-40.0-42.0**	12.50	77	1.27	0.00	4.59	16.12	76.65	0.03	0.00	1.36	118.76	1.08	37.70	4.71	0.01	0.07	92.0	1.9	6.0	Sand
**SB02-42.0-44.0**	13.11	77	6.24	0.00	8.06	32.21	53.49	0.00	0.00	0.00	202.52	0.60	33.97	4.49	0.02	0.05	85.1	3.9	11.0	Loamy Sand
**SB02-44.0-48.0**	14.02	84	0.01	10.26	1.75	6.68	81.30	0.00	0.00	0.00	32.27	0.66	81.28	5.13	0.03	0.03	97.1	0.9	2.0	Sand
**SB02-48.0-50.0**	14.94	83	0.00	3.42	1.71	1.70	93.17	0.00	0.00	0.00	13.21	1.34	42.43	5.08	0.02	0.02	98.1	0.0	1.9	Sand
**SB02-50.0-52.0**	15.54	85	0.84	24.95	2.63	12.28	59.30	0.00	0.00	0.00	33.96	1.88	46.99	4.92	0.02	0.05	96.1	0.0	3.9	Sand
**SB02-52.0-54.0**	16.15	79	7.30	0.00	13.23	31.98	45.39	0.07	0.00	2.02	83.98	0.93	40.67	4.67	0.03	0.04	81.0	9.9	9.0	Loamy Sand
**SB02-54.0-56.0**	16.76	79	10.48	0.00	10.96	33.70	40.89	0.03	0.00	3.98	48.74	0.73	32.06	4.85	0.04	0.05	88.3	3.7	8.1	Loamy Sand
**SB02-56.0-58.0**	17.37	78	0.00	6.43	9.33	12.57	65.13	0.00	0.00	6.54	25.76	2.86	30.99	4.88	0.03	0.02	94.0	0.9	5.0	Sand
**SB02-58.0-60.0**	17.98	81	0.00	4.97	10.67	13.19	62.20	0.00	0.00	8.97	34.35	1.49	45.52	5.00	0.03	0.02	94.0	1.0	5.0	Sand
**SB02-60.0-62.0**	18.59	83	2.14	0.01	10.32	6.36	71.33	0.01	0.00	6.51	39.50	2.08	39.43	4.94	0.03	0.02	95.0	1.0	4.0	Sand
**SB02-62.0-64.0**	19.20	79	0.00	0.00	12.96	10.67	70.47	0.03	0.00	2.91	47.41	1.87	32.98	4.85	0.05	0.03	86.0	8.0	6.0	Loamy Sand
**SB02-64.0-66.0**	19.81	77	0.00	0.00	16.42	11.61	71.97	0.03	0.00	0.00	250.69	3.04	118.26	4.16	0.04	0.44	31.9	42.0	26.1	Loam

Supplementary Table 8. **Geochemical soil parameters for Residual Field 2 (continued) for hand-augured surface soils (SF) and cored soil bored (SB) samples.**

Sample ID Depth Increment (ft)	Mid-depth (m)	Mehlich 1 (mg/kg)														Soluble Anions (mg/kg)					ppm $CaCO_3$/pH		CEC meq/ 100 g	Base Saturation (% of CEC)
		Ca	Cd	Cr	Cu	Fe	K	Mg	Mn	Mo	Na	Ni	P	Pb	Zn	Cl	F	NO_3	PO_4	SO_4	LBC	LBC_{eq}		
**SF201-0.0-0.5**	0.08	556.07	<0.04	<0.04	1.22	14.90	23.36	6.95	8.17	<0.04	6.72	0.11	49.57	0.94	4.53	<2.34	<0.78	5.53	<11.70	509.8	287.00	832.30	6.12	47.81
**SF202-0.0-0.5**	0.08	568.38	<0.04	<0.04	1.12	5.31	27.37	13.41	7.31	<0.04	8.37	0.15	46.98	0.91	5.63	<2.59	<0.86	8.57	<12.93	18.0	236.00	678.08	4.62	66.24
**SF203-0.0-0.5**	0.08	572.11	<0.04	0.05	1.09	8.08	26.18	12.90	7.25	<0.04	7.54	0.21	61.91	0.90	5.10	<2.45	<0.82	5.89	<12.24	27.2	207.00	571.63	4.37	70.18
**SF204-0.0-0.5**	0.08	596.08	<0.04	0.05	1.32	9.07	73.99	18.46	11.33	<0.04	7.00	0.16	60.33	0.94	6.48	<2.42	<0.81	9.12	<12.10	159.0	258.00	748.20	5.30	63.29
**SB02-0.0-0.5**	0.08	383.23	<0.04	0.06	1.10	11.19	32.08	11.41	5.43	<0.04	6.97	0.12	45.62	1.21	3.58	<2.46	0.97	6.13	<12.29	19.0	191.00	512.89	3.49	60.89
**SB02-0.5-1.0**	0.23	322.26	<0.04	0.04	0.28	5.51	53.07	13.70	2.46	<0.04	6.48	<0.04	6.63	1.01	0.41	<2.47	1.62	2.53	<12.37	71.6	160.00	399.09	2.57	73.58
**SB02-1.0-1.5**	0.38	412.17	<0.04	<0.04	<0.21	4.21	68.42	18.83	1.64	<0.04	6.15	<0.04	1.27	0.64	<0.21	<2.90	<0.97	2.98	<14.49	57.9	216.00	604.66	3.93	61.55
**SB02-1.5-2.0**	0.53	292.55	<0.04	<0.04	<0.22	4.92	93.12	32.98	<0.22	<0.04	5.60	<0.04	0.35	0.83	<0.22	<2.75	<0.92	2.33	<13.76	64.8	282.00	817.80	5.21	38.43
**SB02-2.0-2.5**	0.69	266.40	<0.05	<0.05	<0.24	4.18	98.61	60.21	<0.24	<0.05	6.71	<0.05	0.37	0.69	<0.24	<2.76	<0.92	2.31	<13.79	87.7	425.00	1232.50	7.29	29.01
**SB02-2.5-3.0**	0.84	255.97	<0.04	<0.04	<0.22	3.34	91.14	71.81	<0.22	<0.04	6.13	<0.04	0.39	0.83	<0.22	3.42	<0.91	2.12	<13.70	101.9	488.00	1415.20	8.48	25.22
**SB02-3.0-3.5**	0.99	235.83	<0.04	<0.04	<0.21	2.91	71.08	75.03	<0.21	<0.04	7.71	<0.04	0.35	0.91	<0.21	5.87	<0.92	3.16	<13.82	92.6	519.00	1505.10	8.94	22.59
**SB02-3.5-4.0**	1.14	197.46	<0.04	<0.04	<0.22	2.65	51.53	66.30	<0.22	<0.04	7.74	<0.04	0.24	1.62	<0.22	14.77	<0.90	5.96	<13.45	39.8	533.00	1545.70	8.63	19.76
**SB02-4.0-4.5**	1.30	162.73	<0.04	<0.04	<0.21	4.67	28.68	59.96	0.63	<0.04	8.12	<0.04	0.44	0.88	<0.21	22.98	<0.87	7.65	<13.10	<10.9	643.00	1864.70	10.00	14.22
**SB02-4.5-5.0**	1.45	134.01	<0.04	<0.04	<0.22	3.10	21.17	63.54	<0.22	<0.04	8.26	<0.04	0.15	1.08	<0.22	29.17	<0.90	10.29	<13.42	<11.2	925.00	2682.50	14.27	9.04
**SB02-5.0-6.0**	1.68	83.75	<0.04	<0.04	<0.21	2.28	11.57	49.45	<0.21	<0.04	7.10	<0.04	0.24	0.81	0.21	24.41	<0.89	8.60	<13.39	<11.2	835.00	2421.50	12.03	7.41
**SB02-6.0-7.0**	1.98	39.08	<0.04	<0.04	<0.21	1.63	5.66	23.79	<0.21	<0.04	7.26	<0.04	0.19	1.02	<0.21	20.58	<0.82	6.53	<12.35	<10.3	692.00	2006.80	9.99	4.40
**SB02-7.0-8.0**	2.29	43.36	<0.04	<0.04	<0.21	1.65	9.20	38.90	<0.21	<0.04	9.87	<0.04	0.10	0.75	<0.21	27.84	<0.87	7.74	<13.04	<10.9	951.00	2757.90	14.12	4.30
**SB02-8.0-9.0**	2.59	29.70	<0.04	<0.04	<0.21	2.27	8.10	30.71	<0.21	<0.04	8.46	<0.04	0.15	0.65	<0.21	29.41	<0.90	6.84	<13.54	<11.3	1037.00	3007.30	16.04	2.88
**SB02-9.0-10.0**	2.90	26.94	<0.04	<0.04	<0.22	2.96	8.28	30.58	<0.22	<0.04	9.51	<0.04	0.22	0.72	<0.22	31.73	<0.91	6.63	<13.57	<11.3	975.00	2827.50	14.48	3.12
**SB02-10.0-12.0**	3.35	27.80	<0.04	0.05	<0.23	3.40	13.48	33.93	<0.23	<0.04	13.11	<0.04	0.21	0.81	<0.23	39.27	<1.00	8.34	<15.08	<12.6	911.00	2641.90	14.15	3.63
**SB02-12.0-14.0**	3.96	49.62	<0.05	0.19	0.62	21.52	16.52	27.46	1.80	<0.05	12.65	0.19	0.59	0.65	0.31	33.82	<1.01	5.68	<15.19	<12.7	650.00	1885.00	9.70	5.92
**SB02-14.0-16.0**	4.57	33.75	<0.05	<0.05	<0.24	1.64	16.16	23.97	<0.24	<0.05	11.28	<0.05	0.29	0.63	<0.24	26.19	<1.04	8.41	<15.67	<13.1	839.00	2433.10	12.48	3.68
**SB02-16.0-18.0**	5.18	40.22	<0.04	0.08	0.34	6.39	22.67	29.93	0.30	<0.04	11.16	<0.04	0.37	0.78	<0.22	20.82	<0.98	15.27	<14.78	<12.3	659.00	1911.10	10.11	5.51
**SB02-18.0-20.0**	5.79	38.56	<0.05	<0.05	0.29	2.46	22.47	25.91	<0.23	<0.05	11.04	<0.05	0.34	0.68	<0.23	18.13	<0.94	13.91	<14.08	<11.7	795.00	2305.50	12.32	4.17
**SB02-20.0-22.0**	6.40	16.85	<0.04	0.05	0.22	1.72	17.02	19.90	<0.22	<0.04	10.37	<0.04	0.26	0.65	<0.22	11.06	<0.95	5.20	<14.32	<11.9	557.00	1615.30	8.22	4.12
**SB02-22.0-24.0**	7.01	14.88	<0.04	<0.04	<0.22	0.88	16.39	15.59	<0.22	<0.04	11.98	<0.04	0.28	0.50	<0.22	10.72	<0.93	3.57	<13.89	<11.6	580.00	1682.00	8.78	3.40
**SB02-24.0-26.0**	7.62	19.72	<0.04	0.05	0.24	1.45	19.89	20.44	<0.21	<0.04	12.68	<0.04	0.31	0.46	<0.21	9.09	<0.90	2.83	<13.45	<11.2	569.00	1650.10	8.49	4.42
**SB02-26.0-28.0**	8.23	29.38	<0.04	0.15	0.27	1.63	21.82	24.25	0.36	<0.04	11.40	<0.04	0.30	0.44	<0.21	6.34	<0.90	2.17	<13.48	<11.2	649.00	1882.10	9.64	4.71
**SB02-28.0-30.0**	8.84	66.03	<0.05	0.23	0.47	2.18	29.90	42.94	1.50	<0.05	12.53	0.06	0.27	0.23	0.30	4.38	<1.02	<1.73	<15.27	<12.7	634.00	1838.60	9.35	8.76
**SB02-30.0-32.0**	9.45	51.59	<0.04	0.05	<0.20	1.54	25.23	43.44	4.07	<0.04	7.88	0.06	0.12	0.33	0.38	3.18	<0.89	<1.50	<13.27	<11.1	526.00	1525.40	8.44	8.52
**SB02-32.0-34.0**	10.06	28.48	<0.04	<0.04	<0.21	1.30	15.24	22.94	5.11	<0.04	6.43	<0.04	0.14	0.55	<0.21	3.14	<0.87	<1.48	<13.10	<10.9	419.00	1215.10	6.26	6.40
**SB02-34.0-36.0**	10.67	20.69	<0.04	<0.04	<0.20	1.09	14.68	18.54	0.40	<0.04	6.05	<0.04	0.46	1.44	<0.20	4.20	<0.86	<1.47	<12.96	<10.8	471.00	1365.90	7.34	4.38
**SB02-36.0-38.0**	11.28	13.10	<0.04	0.04	<0.18	3.98	4.33	7.30	0.53	<0.04	4.54	<0.04	0.49	0.42	<0.18	<2.23	<0.74	<1.27	<11.17	<9.3	73.00	79.73	0.52	30.37
**SB02-38.0-40.0**	11.89	31.73	<0.04	<0.04	0.22	1.95	23.15	30.96	0.78	<0.04	7.89	<0.04	1.37	0.15	<0.22	4.88	<0.92	<1.56	<13.73	<11.4	724.00	2099.60	11.47	4.45
**SB02-40.0-42.0**	12.50	15.06	<0.04	<0.04	<0.19	1.34	5.22	7.27	0.25	<0.04	5.79	<0.04	0.63	0.29	<0.19	3.47	<0.76	<1.29	<11.41	<9.5	214.00	597.32	2.91	5.99
**SB02-42.0-44.0**	13.11	14.83	<0.04	<0.04	<0.18	0.93	8.46	9.37	0.30	<0.04	5.78	<0.04	0.77	<0.11	<0.18	3.82	<0.78	1.78	<11.74	<9.8	414.00	1200.60	6.23	3.20
**SB02-44.0-48.0**	14.02	11.39	<0.03	0.05	<0.17	3.81	2.34	3.22	0.28	<0.03	4.63	<0.03	0.54	<0.10	<0.17	2.23	<0.68	1.57	<10.19	<8.5	41.00	0.00	0.11	228.49
**SB02-48.0-50.0**	14.94	10.56	<0.03	<0.03	<0.17	1.64	<1.69	2.80	0.50	<0.03	4.65	<0.03	0.25	<0.10	<0.17	2.18	<0.70	1.46	<10.44	<8.7	34.00	0.00	0.10	347.30
**SB02-50.0-52.0**	15.54	11.10	<0.03	<0.03	<0.17	1.97	2.05	3.16	0.80	<0.03	4.55	<0.03	0.22	0.11	<0.17	<2.12	<0.71	<1.20	<10.58	<8.8	40.00	0.00	0.11	261.12
**SB02-52.0-54.0**	16.15	14.78	<0.04	0.05	<0.18	4.54	3.83	5.76	0.47	<0.04	5.61	0.04	0.29	0.24	<0.18	2.85	<0.76	1.50	<11.34	<9.5	82.00	112.76	0.68	22.90
**SB02-54.0-56.0**	16.76	38.32	<0.04	<0.04	<0.18	1.43	3.32	6.19	0.57	<0.04	5.68	<0.04	1.14	0.16	0.27	<2.18	<0.73	<1.24	<10.91	<9.1	52.00	2.64	0.29	96.06
**SB02-56.0-58.0**	17.37	13.76	<0.04	<0.04	<0.19	0.99	2.49	3.81	0.33	<0.04	5.89	<0.04	0.21	<0.11	<0.19	2.41	<0.74	<1.26	<11.15	<9.3	43.00	0.00	0.13	197.28
**SB02-58.0-60.0**	17.98	11.63	<0.03	<0.03	<0.17	0.88	1.98	3.61	0.24	<0.03	5.30	<0.03	0.19	0.17	<0.17	2.48	<0.74	1.32	<11.09	<9.2	41.00	0.00	0.12	229.72
**SB02-60.0-62.0**	18.59	12.80	<0.03	<0.03	<0.17	1.27	2.25	3.49	0.82	<0.03	5.47	<0.03	0.22	0.13	<0.17	<2.20	<0.73	<1.25	<11.01	<9.2	41.00	0.00	0.12	226.78
**SB02-62.0-64.0**	19.20	15.16	<0.04	<0.04	<0.18	1.37	3.55	4.72	1.15	<0.04	5.44	<0.04	0.26	0.22	<0.18	<2.09	<0.70	<1.18	<10.45	<8.7	56.00	17.32	0.22	66.51
**SB02-64.0-66.0**	19.81	35.67	<0.05	0.08	1.47	13.95	15.15	17.76	1.86	<0.05	9.83	0.38	0.36	1.15	0.39	<3.06	<1.02	<1.73	<15.31	61.1	371.00	1075.90	6.52	6.26

Supplementary Table 9. **Geochemical groundwater parameters.**

Parameter	Site 1		Site 2	
	Shallow	Deep	Shallow	Deep
Field Dissolved Oxygen (mg/L)	4.4	6.7	6.1	2.3
Field Temperature (°C)	23.0	21.3	21.0	21.7
Field Turbidity (NTU)	1.0	8.1	7.3	6.8
Field Conductivity (µS/cm)	74.7	89.2	238.3	37.4
Lab Conductivity (µS/cm)	62.4	80.2	217.8	30.4
Field pH (SU)	4.50	4.70	4.20	5.14
Lab pH (SU)	5.20	5.41	4.29	6.03
Ca (ppm)	0.81	1.73	9.80	0.97
Mg (ppm)	0.92	3.00	8.75	0.42
Na (ppm)	8.46	6.33	10.44	3.19
K (ppm)	<0.50	<0.50	2.47	0.74
Si (ppm)	3.01	3.45	4.02	4.19
Al (ppm)	0.22	0.30	2.46	<0.10
Fe (ppm)	0.22	0.92	0.39	1.14
Mn (ppm)	<0.05	0.09	<0.05	<0.05
Cu (ppm)	<0.05	<0.05	<0.05	<0.05
Mo (ppm)	<0.010	<0.010	<0.010	<0.010
B (ppm)	<0.01	<0.01	0.02	<0.01
P (ppm)	<0.02	<0.02	<0.02	<0.02
Zn (ppm)	<0.05	<0.05	<0.05	<0.05
Cr (ppb)	<10	<10	<10	<10
Ni (ppb)	<10	<10	<10	<10
Cl (ppm)	11.17	8.86	19.09	4.04
NO_3 (ppm)	2.77	5.90	17.67	1.17
F (ppm)	<0.20	<0.20	<0.20	<0.20
PO_4 (ppm)	<3.00	<3.00	<3.00	<3.00
SO_4 (ppm)	<2.50	<2.50	<2.50	<2.50
CO_2 (ppm)	1.55	6.85	<205	4.15
Hardness (ppm as $CaCO_3$)	5.79	16.66	60.50	4.17
Alkalinity (ppm as $CaCO_3$)	0.12	0.89	<2.00	2.24

Supplementary Table 10. **pH-Dependent Electrostatic Surface Charge of Selected Mineral(oid)s in Field 1.**

Sample ID	Mid-depth	Electrostatic Surface Charge (1) (meq/kg soil)				
Depth Increment (ft)	(m)	Extracted HFO	Extracted HAO	X-Ray Goethite	X-Ray Gibbsite	X-Ray Kaolinite
RPFT-SF101-0.0-0.5	0.076	3.66E-02	1.29E-01	0.00E+00	0.00E+00	-9.67E-05
RPFT-SF102-0.0-0.5	0.076	7.27E-02	1.96E-01	0.00E+00	0.00E+00	6.50E-05
RPFT-SF103-0.0-0.5	0.076	6.37E-03	7.71E-02	0.00E+00	0.00E+00	-1.93E-04
RPFT-SF104-0.0-0.5	0.076	8.91E-02	2.21E-01	0.00E+00	0.00E+00	3.11E-04
RPFT-SB01-0.0-0.5	0.076	6.21E-02	1.79E-01	1.44E-01	5.24E-01	-2.17E-04
RPFT-SB01-0.5-1.0	0.229	2.53E-02	1.81E-01	9.97E-01	1.03E+00	-2.07E-03
RPFT-SB01-1.0-1.5	0.381	9.61E-03	1.42E-01	8.91E-01	9.61E-01	-4.24E-03
RPFT-SB01-1.5-2.0	0.533	1.53E-02	1.45E-01	1.02E+00	1.07E+00	-2.33E-03
RPFT-SB01-2.0-2.5	0.686	9.42E-02	7.61E-01	4.96E+00	4.82E+00	2.01E-02
RPFT-SB01-2.5-3.0	0.838	1.33E-01	1.11E+00	3.37E+00	6.86E+00	2.32E-02
RPFT-SB01-3.0-3.5	0.991	9.58E-02	9.21E-01	5.86E+00	6.84E+00	2.15E-02
RPFT-SB01-3.5-4.0	1.143	1.55E-01	9.85E-01	6.96E+00	6.43E+00	2.29E-02
RPFT-SB01-4.0-4.5	1.295	1.63E-01	9.01E-01	1.57E+00	6.91E+00	2.50E-02
RPFT-SB01-4.5-5.0	1.448	3.14E-02	1.05E+00	4.62E-01	9.61E+00	3.11E-02
RPFT-SB01-5.0-6.0	1.676	5.73E-02	9.57E-01	5.86E+00	9.11E+00	2.59E-02
RPFT-SB01-6.0-7.0	1.981	6.69E-02	1.30E+00	1.53E+00	8.40E+00	3.43E-02
RPFT-SB01-7.0-8.0	2.286	5.68E-02	1.21E+00	0.00E+00	6.32E+00	3.66E-02
RPFT-SB01-8.0-9.0	2.591	6.66E-02	8.66E-01	8.93E-01	9.62E+00	3.15E-02
RPFT-SB01-9.0-10.0	2.896	6.41E-02	9.81E-01	6.58E-01	9.31E+00	3.35E-02
RPFT-SB01-10.0-12.0	3.353	1.68E-01	8.69E-01	7.89E-02	1.13E+01	2.99E-02
RPFT-SB01-12.0-16.0	4.267	7.45E-02	2.29E-01	0.00E+00	4.80E+00	1.90E-02
RPFT-SB01-16.0-18.0	5.182	1.61E-02	2.16E-01	5.09E-01	3.09E+00	2.31E-02
RPFT-SB01-18.0-20.0	5.791	4.33E-02	3.57E-01	1.85E-02	1.58E+00	6.92E-03
RPFT-SB01-20.0-22.0	6.401	1.02E-01	2.73E-01	4.41E-01	3.52E+00	7.92E-03
RPFT-SB01-22.0-24.0	7.010	4.21E-02	3.03E-01	1.86E-01	4.74E-01	6.65E-03
RPFT-SB01-24.0-26.0	7.620	7.87E-02	2.43E-01	4.72E-01	3.30E+00	1.57E-02
RPFT-SB01-26.0-28.0	8.230	3.18E-02	4.13E-01	4.16E-01	1.90E+00	1.09E-02
RPFT-SB01-28.0-30.0	8.839	2.36E-02	1.89E-01	2.31E+00	1.75E+00	1.28E-02
RPFT-SB01-30.0-32.0	9.449	2.54E-02	8.05E-02	1.08E+00	2.17E+00	6.12E-03
RPFT-SB01-32.0-34.0	10.058	7.05E-02	1.37E-01	1.13E-01	1.25E-01	3.01E-03
RPFT-SB01-34.0-36.0	10.668	5.64E-02	1.10E-01	3.41E-03	0.00E+00	8.82E-03
RPFT-SB01-36.0-38.0	11.278	7.05E-02	1.39E-01	2.06E-01	0.00E+00	2.97E-03
RPFT-SB01-38.0-40.0	11.887	7.78E-03	7.23E-02	3.30E-03	1.52E-02	2.26E-04
RPFT-SB01-40.0-42.0	12.497	8.57E-03	6.03E-02	1.37E-02	6.45E-02	3.81E-04
RPFT-SB01-42.0-44.0	13.106	1.31E-02	5.68E-02	3.75E-01	1.23E+00	1.43E-03
RPFT-SB01-44.0-46.0	13.716	9.87E-03	5.16E-02	3.98E-01	5.61E-02	2.12E-03
RPFT-SB01-46.0-48.0	14.326	2.56E-02	8.03E-02	4.60E-01	0.00E+00	2.67E-04
RPFT-SB01-48.0-50.0	14.935	2.30E-02	5.97E-02	1.55E-01	1.97E+00	8.64E-04
RPFT-SB01-50.0-52.0	15.545	1.93E-02	6.25E-02	0.00E+00	5.76E+00	2.38E-03
RPFT-SB01-52.0-56.0	16.459	2.90E-02	5.66E-02	0.00E+00	2.25E+00	4.89E-03
RPFT-SB01-56.0-58.0	17.374	4.36E-02	8.89E-02	1.39E-02	0.00E+00	1.39E-03
RPFT-SB01-58.0-60.0	17.983	3.07E-02	1.03E-01	0.00E+00	0.00E+00	1.90E-03
RPFT-SB01-60.0-64.0	18.898	1.96E-02	5.65E-02	4.14E+00	2.02E-01	2.10E-03

Supplementary Table 11. **pH-Dependent Electrostatic Surface Charge of Selected Mineral(oid)s in Field 2.**

Sample ID	Mid-depth	Electrostatic Surface Charge (1) (meq/kg soil)				
Depth Increment (ft)	(m)	Extracted HFO	Extracted HAO	X-Ray Goethite	X-Ray Gibbsite	X-Ray Kaolinite
RPFT-SF201-0.0-0.5	0.076	2.11E-01	5.45E-01	0.00E+00	0.00E+00	4.69E-04
RPFT-SF202-0.0-0.5	0.076	9.55E-02	2.80E-01	2.06E-03	9.73E-01	5.61E-04
RPFT-SF203-0.0-0.5	0.076	1.08E-01	2.90E-01	0.00E+00	0.00E+00	2.78E-04
RPFT-SF204-0.0-0.5	0.076	1.57E-01	3.83E-01	0.00E+00	6.87E-01	2.23E-03
RPFT-SB02-0.0-0.5	0.076	1.24E-01	3.38E-01	1.12E-02	1.12E-02	5.79E-04
RPFT-SB02-0.5-1.0	0.229	6.70E-02	2.56E-01	7.06E-03	6.00E-01	5.16E-04
RPFT-SB02-1.0-1.5	0.381	1.51E-01	6.71E-01	1.73E-02	1.12E+00	2.03E-03
RPFT-SB02-1.5-2.0	0.533	1.40E-01	1.15E+00	4.89E-02	3.82E+00	8.88E-03
RPFT-SB02-2.0-2.5	0.686	1.92E-01	1.50E+00	2.38E-01	5.95E+00	1.20E-02
RPFT-SB02-2.5-3.0	0.838	2.28E-01	1.65E+00	8.73E-02	5.50E+00	1.35E-02
RPFT-SB02-3.0-3.5	0.991	1.73E-01	1.43E+00	2.80E-01	5.56E+00	1.44E-02
RPFT-SB02-3.5-4.0	1.143	1.69E-01	1.48E+00	4.43E-01	5.73E+00	1.65E-02
RPFT-SB02-4.0-4.5	1.295	3.00E-01	1.68E+00	1.83E+00	7.00E+00	1.85E-02
RPFT-SB02-4.5-5.0	1.448	1.71E-01	1.57E+00	2.41E+00	6.42E+00	2.01E-02
RPFT-SB02-5.0-6.0	1.676	9.92E-02	1.43E+00	5.24E-01	7.01E+00	1.58E-02
RPFT-SB02-6.0-7.0	1.981	5.03E-02	8.39E-01	3.73E-03	3.90E+00	1.59E-02
RPFT-SB02-7.0-8.0	2.286	6.38E-02	1.20E+00	3.28E-01	6.42E+00	1.72E-02
RPFT-SB02-8.0-9.0	2.591	6.43E-02	1.40E+00	6.59E-02	8.00E+00	2.41E-02
RPFT-SB02-9.0-10.0	2.896	6.44E-02	1.00E+00	8.84E-01	3.68E+00	1.80E-02
RPFT-SB02-10.0-12.0	3.353	1.05E-01	1.46E+00	4.10E-03	6.24E+00	2.20E-02
RPFT-SB02-12.0-14.0	3.962	3.05E-01	9.90E-01	0.00E+00	1.55E+00	1.76E-02
RPFT-SB02-14.0-16.0	4.572	5.67E-02	7.67E-01	7.78E-03	2.10E+00	1.29E-02
RPFT-SB02-16.0-18.0	5.182	1.00E-01	1.09E+00	0.00E+00	8.10E+00	2.04E-02
RPFT-SB02-18.0-20.0	5.791	4.35E-02	9.32E-01	0.00E+00	3.24E+00	1.58E-02
RPFT-SB02-20.0-22.0	6.401	3.66E-02	6.27E-01	0.00E+00	4.95E+00	1.17E-02
RPFT-SB02-22.0-24.0	7.010	3.78E-02	6.35E-01	0.00E+00	6.08E-01	1.34E-02
RPFT-SB02-24.0-26.0	7.620	4.48E-02	6.35E-01	0.00E+00	3.02E+00	1.84E-02
RPFT-SB02-26.0-28.0	8.230	7.00E-02	5.00E-01	5.99E-01	3.84E+00	1.48E-02
RPFT-SB02-28.0-30.0	8.839	1.49E-01	7.52E-01	1.60E+00	3.34E+00	1.37E-02
RPFT-SB02-30.0-32.0	9.449	2.77E-01	6.35E-01	0.00E+00	8.56E+00	1.64E-02
RPFT-SB02-32.0-34.0	10.058	8.74E-02	4.53E-01	0.00E+00	7.36E+00	1.49E-02
RPFT-SB02-34.0-36.0	10.668	4.79E-02	4.06E-01	0.00E+00	7.03E+00	1.71E-02
RPFT-SB02-36.0-38.0	11.278	6.13E-02	1.59E-01	0.00E+00	2.25E+00	7.55E-03
RPFT-SB02-38.0-40.0	11.887	1.84E-01	1.04E+00	0.00E+00	9.77E+00	2.56E-02
RPFT-SB02-40.0-42.0	12.497	4.20E-02	2.39E-01	0.00E+00	7.30E-01	8.33E-03
RPFT-SB02-42.0-44.0	13.106	4.18E-02	4.81E-01	0.00E+00	4.23E+00	1.97E-02
RPFT-SB02-44.0-48.0	14.021	7.37E-02	4.74E-02	3.00E+00	4.19E-03	2.46E-03
RPFT-SB02-48.0-50.0	14.935	3.95E-02	2.01E-02	1.02E+00	0.00E+00	6.54E-04
RPFT-SB02-50.0-52.0	15.545	4.73E-02	5.84E-02	8.07E+00	4.12E-01	5.38E-03
RPFT-SB02-52.0-54.0	16.154	4.61E-02	1.74E-01	0.00E+00	4.33E+00	1.70E-02
RPFT-SB02-54.0-56.0	16.764	3.34E-02	8.84E-02	0.00E+00	5.42E+00	1.56E-02
RPFT-SB02-56.0-58.0	17.374	3.18E-02	4.57E-02	2.12E+00	0.00E+00	5.69E-03
RPFT-SB02-58.0-60.0	17.983	4.41E-02	5.56E-02	1.55E+00	0.00E+00	5.42E-03
RPFT-SB02-60.0-62.0	18.593	3.93E-02	6.69E-02	3.20E-03	1.03E+00	2.74E-03
RPFT-SB02-62.0-64.0	19.202	3.44E-02	8.60E-02	0.00E+00	0.00E+00	4.94E-03
RPFT-SB02-64.0-66.0	19.812	1.67E-01	7.61E-01	0.00E+00	0.00E+00	9.07E-03

Supplementary Table 12. **Moisture status, Field 1.**

Sample ID	Mid-depth	Hydrostatic Pressure (1)		Moisture Content		Air-Water Interface	
Depth Increment (ft)	(m)	(cm H_2O)	(milli-atm)	Calc. Pore Saturation (2) (vol %)	Lab Meas. Solids (mass %)	Surf. Area Est. (1) (cm^2/cm^3)	Med. Diam. (2) (cm^2/cm^3)
RPFT-SF101-0.0-0.5	0.076	-358	-347	23.1	92	991	1545
RPFT-SF102-0.0-0.5	0.076	-358	-347	23.1	89	991	1545
RPFT-SF103-0.0-0.5	0.076	-358	-347	17.4	95	884	1329
RPFT-SF104-0.0-0.5	0.076	-358	-347	23.1	93	991	1545
RPFT-SB01-0.0-0.5	0.076	-358	-347	41.8	93	748	1407
RPFT-SB01-0.5-1.0	0.229	-343	-332	54.0	89	579	1041
RPFT-SB01-1.0-1.5	0.381	-328	-317	54.1	89	577	1037
RPFT-SB01-1.5-2.0	0.533	-312	-302	54.2	88	571	1032
RPFT-SB01-2.0-2.5	0.686	-297	-288	54.4	86	567	1026
RPFT-SB01-2.5-3.0	0.838	-282	-273	54.6	85	563	1018
RPFT-SB01-3.0-3.5	0.991	-267	-258	54.9	86	557	1008
RPFT-SB01-3.5-4.0	1.143	-252	-243	55.2	86	551	997
RPFT-SB01-4.0-4.5	1.295	-236	-229	55.6	87	538	982
RPFT-SB01-4.5-5.0	1.448	-221	-214	75.6	84	211	569
RPFT-SB01-5.0-6.0	1.676	-198	-192	76.9	83	194	526
RPFT-SB01-6.0-7.0	1.981	-168	-162	58.9	82	472	866
RPFT-SB01-7.0-8.0	2.286	-137	-133	61.8	82	418	771
RPFT-SB01-8.0-9.0	2.591	-107	-103	66.2	80	342	636
RPFT-SB01-9.0-10.0	2.896	-76	-74	72.9	80	239	453
RPFT-SB01-10.0-12.0	3.353	-31	-30	90.2	82	54	111
RPFT-SB01-12.0-16.0	4.267	61	59	82.6	85	84	148
RPFT-SB01-16.0-18.0	5.182	152	147	89.9	75	31	54
RPFT-SB01-18.0-20.0	5.791	213	206	82.6	76	84	148
RPFT-SB01-20.0-22.0	6.401	274	265	89.9	74	31	54
RPFT-SB01-22.0-24.0	7.010	335	324	89.9	77	31	54
RPFT-SB01-24.0-26.0	7.620	396	383	82.6	79	84	148
RPFT-SB01-26.0-28.0	8.230	457	442	82.6	77	84	148
RPFT-SB01-28.0-30.0	8.839	518	501	89.9	81	31	54
RPFT-SB01-30.0-32.0	9.449	579	560	89.9	81	31	54
RPFT-SB01-32.0-34.0	10.058	640	619	89.9	73	31	54
RPFT-SB01-34.0-36.0	10.668	701	678	89.9	81	31	54
RPFT-SB01-36.0-38.0	11.278	762	737	89.9	75	31	54
RPFT-SB01-38.0-40.0	11.887	823	796	89.9	80	31	54
RPFT-SB01-40.0-42.0	12.497	884	855	89.9	76	31	54
RPFT-SB01-42.0-44.0	13.106	945	914	89.9	76	31	54
RPFT-SB01-44.0-46.0	13.716	1006	973	89.9	80	31	54
RPFT-SB01-46.0-48.0	14.326	1067	1032	89.9	82	31	54
RPFT-SB01-48.0-50.0	14.935	1128	1091	89.9	78	31	54
RPFT-SB01-50.0-52.0	15.545	1189	1150	89.9	81	31	54
RPFT-SB01-52.0-56.0	16.459	1280	1239	89.9	79	31	54
RPFT-SB01-56.0-58.0	17.374	1372	1327	89.9	73	31	54
RPFT-SB01-58.0-60.0	17.983	1433	1386	89.9	77	31	54
RPFT-SB01-60.0-64.0	18.898	1524	1475	89.9	81	31	54

1) Hydrostatic pressure calculated as height above water table, assumes equilibrium capillary distribution
2) Calculated with Boltzman Distribution Equation of Bumb et al. [39], see SI text
3) Calculated with BET-based Equation 4 of Brusseau, see SI text
4) Calculated with d50-based Equation 5 of Brusseau, see SI text

Supplementary Table 13. **Moisture status, Field 2.**

Sample ID	Mid-depth	Hydrostatic Pressure		Moisture Content		Air-Water Interface	
Depth Increment (ft)	(m)	(cm H_2O)	(milli-atm)	Calc. Pore Saturation (vol %)	Lab Meas. Solids (mass %)	Surf. Area Est (1) (cm^2/cm^3)	Med. Diam. (2) (cm^2/cm^3)
RPFT-SF201-0.0-0.5	0.076	-450	-435	41.5	93	761	1419
RPFT-SF202-0.0-0.5	0.076	-450	-435	41.5	92	761	1419
RPFT-SF203-0.0-0.5	0.076	-450	-435	41.5	92	761	1419
RPFT-SF204-0.0-0.5	0.076	-450	-435	41.5	91	761	1419
RPFT-SB02-0.0-0.5	0.076	-450	-435	41.5	93	761	1419
RPFT-SB02-0.5-1.0	0.229	-434	-420	41.5	94	760	1418
RPFT-SB02-1.0-1.5	0.381	-419	-406	41.6	93	754	1416
RPFT-SB02-1.5-2.0	0.533	-404	-391	53.7	89	583	1051
RPFT-SB02-2.0-2.5	0.686	-389	-376	53.7	88	582	1049
RPFT-SB02-2.5-3.0	0.838	-373	-361	53.8	87	581	1047
RPFT-SB02-3.0-3.5	0.991	-358	-347	53.9	88	579	1044
RPFT-SB02-3.5-4.0	1.143	-343	-332	54.0	87	577	1041
RPFT-SB02-4.0-4.5	1.295	-328	-317	71.4	86	274	723
RPFT-SB02-4.5-5.0	1.448	-312	-302	75.8	85	217	1990
RPFT-SB02-5.0-6.0	1.676	-290	-280	54.5	87	566	1022
RPFT-SB02-6.0-7.0	1.981	-259	-251	55.0	88	555	1003
RPFT-SB02-7.0-8.0	2.286	-229	-221	55.8	87	538	974
RPFT-SB02-8.0-9.0	2.591	-198	-192	57.0	85	513	930
RPFT-SB02-9.0-10.0	2.896	-168	-162	58.9	85	476	866
RPFT-SB02-10.0-12.0	3.353	-122	-118	82.4	79	130	357
RPFT-SB02-12.0-14.0	3.962	-61	-59	77.5	78	181	344
RPFT-SB02-14.0-16.0	4.572	0	0	92.1	77	41	85
RPFT-SB02-16.0-18.0	5.182	61	59	92.1	71	41	85
RPFT-SB02-18.0-20.0	5.791	122	118	92.1	74	41	85
RPFT-SB02-20.0-22.0	6.401	183	177	87.1	71	69	145
RPFT-SB02-22.0-24.0	7.010	244	236	87.1	73	69	145
RPFT-SB02-24.0-26.0	7.620	305	295	87.1	72	69	145
RPFT-SB02-26.0-28.0	8.230	366	354	92.1	71	41	85
RPFT-SB02-28.0-30.0	8.839	427	413	92.1	74	41	85
RPFT-SB02-30.0-32.0	9.449	488	472	87.1	76	69	145
RPFT-SB02-32.0-34.0	10.058	549	531	87.1	78	69	145
RPFT-SB02-34.0-36.0	10.668	610	590	87.1	73	69	145
RPFT-SB02-36.0-38.0	11.278	671	649	89.9	79	31	54
RPFT-SB02-38.0-40.0	11.887	732	708	92.1	75	41	85
RPFT-SB02-40.0-42.0	12.497	792	767	89.9	77	31	54
RPFT-SB02-42.0-44.0	13.106	853	826	82.6	77	86	148
RPFT-SB02-44.0-48.0	14.021	945	914	89.9	84	31	54
RPFT-SB02-48.0-50.0	14.935	1036	1003	89.9	83	31	54
RPFT-SB02-50.0-52.0	15.545	1097	1062	89.9	85	31	54
RPFT-SB02-52.0-54.0	16.154	1158	1121	82.6	79	86	148
RPFT-SB02-54.0-56.0	16.764	1219	1180	82.6	79	86	148
RPFT-SB02-56.0-58.0	17.374	1280	1239	89.9	78	31	54
RPFT-SB02-58.0-60.0	17.983	1341	1298	89.9	81	31	54
RPFT-SB02-60.0-62.0	18.593	1402	1357	89.9	83	31	54
RPFT-SB02-62.0-64.0	19.202	1463	1416	82.6	79	86	148
RPFT-SB02-64.0-66.0	19.812	1524	1475	81.3	77	126	828

1) Hydrostatic pressure calculated as height above water table, assumes equilibrium capillary distribution
2) Calculated with Boltzman Distribution Equation of Bumb et al. [39], see SI text
3) Calculated with BET-based Equation 4 of Brusseau, see SI text
4) Calculated with d50-based Equation 5 of Brusseau, see SI text

Supplementary Table 14. **Soil PFAS in Field 1.** No analytes were detected at or above the reporting limit in equipment rinse blanks/field blanks. Note, blank cells represent non-detects.

Sample ID	Depth	PFBA	PFPeA	PFHxA	PFHpA	PFOA	PFNA	PFDA	PFUdA	PFDoA	PFTrDA	PFTeDA	PFBS	PFPeS	PFHxS	PFHpS	PFOS	PFNS	PFDS	4:2FTS	6:2FTS	8:2FTS	N-EtFOSAA	N-MeFOSAA	FOSA	HFPO-DA
Depth Increment (ft)	(m)	(ng/kg)	(ng/kg)	(ng/kg)	(ng/kg)	(ng/kg)	(ng/kg)	(ng/kg)	(ng/kg)	(ng/kg)	(ng/kg)	(ng/kg)	(ng/kg)	(ng/kg)	(ng/kg)	(ng/kg)	(ng/kg)	(ng/kg)	(ng/kg)	(ng/kg)	(ng/kg)	(ng/kg)	(ng/kg)	(ng/kg)	(ng/kg)	(ng/kg)
**RPFT-SF101-0.0-0.5**	0.08	1300	2600	2700	2000	9500	3700	43000	69000	77000	43000	18000	3700	61	270	180	42000	120	400			1200	2200	2400	2200	
**RPFT-SF102-0.0-0.5**	0.08	1300	2900	2900	2200	7400	5200	62000	56000	58000	38000	20000	3200		120	88	25000	56	150			820	390	790	1500	
**RPFT-SF103-0.0-0.5**	0.08	230	450	660	680	2500	1400	13000	20000	42000	22000	12000	880		66	47	6900		150			660	490	1000	430	
**RPFT-SF104-0.0-0.5**	0.08	1100	2300	3000	2400	8800	3100	33000	58000	78000	45000	19000	5500	74	310	140	25000	77	370			810	1000	1800	3600	
**RPFT-SB01-0.0-0.5**	0.08	840	2300	2500	2200	7100	3800	73000	55000	47000	24000	6900	3200		180	100	33000	87	160			940	1100	990	1500	
**RPFT-SB01-0.5-1.0**	0.23	1300	2500	2400	2000	7900	11000	82000	7600	2400	1200	480	3300		150	170	81000	49			150	200	47		250	
**RPFT-SB01-1.0-1.5**	0.38	1400	3000	2700	2300	8400	9900	51000	1800	760	450	250	4200		180	170	81000								53	
**RPFT-SB01-1.5-2.0**	0.53	830	1900	2000	1800	6400	5900	5100	120	210	160		3700		160	110	50000									
**RPFT-SB01-2.0-2.5**	0.69	620	1600	2000	1900	5900	5300	1300		58	43		3100		140	100	31000									
**RPFT-SB01-2.5-3.0**	0.84	480	1200	1600	1300	4200	3400	1200					2800		100	75	16000									
**RPFT-SB01-3.0-3.5**	0.99	290	800	1100	890	3000	1900	340					2100		73	51	8300									
**RPFT-SB01-3.5-4.0**	1.14	810	2100	2400	1800	5800	3600	610					4800		150	110	16000									
**RPFT-SB01-4.5-5.0**	1.45	2300	5000	6000	4100	10000	2700	190	100	120	80		13000	97	330	180	5900									
**RPFT-SB01-5.0-6.0**	1.68	2600	5800	6800	5200	11000	2500	170			54		14000	110	440	180	6800									
**RPFT-SB01-6.0-7.0**	1.98	2500	5700	6900	5000	11000	2500	170					14000	120	450	180	7200									
**RPFT-SB01-7.0-8.0**	2.29	2200	5100	6500	5100	12000	2000						15000	150	590	210	5900									
**RPFT-SB01-9.0-10.0**	2.90	1600	3600	4800	3300	8000	1600						10000	110	430	120	5400									
**RPFT-SB01-10.0-12.0**	3.35	860	2200	3300	2300	4300	370	140					7400	130	460	55	1200									
**RPFT-SB01-18.0-20.0**	5.79	790	1800	3100	2000	5100	700						7300	73	250	59	1900									
**RPFT-SB01-22.0-24.0**	7.01	640	1500	2700	1700	4600	550						6600	63	220	53	1600									
**RPFT-SB01-24.0-26.0**	7.62	640	1500	2600	1800	4100	400						6800	74	250	56	1300									
**RPFT-SB01-26.0-28.0**	8.23	600	1500	2600	1800	4400	520						6500	77	270	67	1700									
**RPFT-SB01-28.0-30.0**	8.84	480	1200	2100	1400	3300	340						4800	63	200	44	990									
**RPFT-SB01-30.0-32.0**	9.45	320	980	1900	1200	3000	390						4000		160	35	1000									
**RPFT-SB01-32.0-34.0**	10.06	150	490	930	590	1500	180						2200		130		630									
**RPFT-SB01-34.0-36.0**	10.67		120	230	160	600	64						450		68		320									
**RPFT-SB01-36.0-38.0**	11.28		130	220	130	300							390				81									
**RPFT-SB01-38.0-40.0**	11.89		63	110	66	150							180													
**RPFT-SB01-40.0-42.0**	12.50		55	96	57	130							180													
**RPFT-SB01-42.0-44.0**	13.11																									
**RPFT-SB01-44.0-46.0**	13.72			44																						
**RPFT-SB01-46.0-48.0**	14.33			46																						
**RPFT-SB01-48.0-50.0**	14.94			60		45																				
**RPFT-SB01-50.0-52.0**	15.54			51																						
**RPFT-SB01-52.0-56.0**	16.46			74		83																				
**RPFT-SB01-56.0-58.0**	17.37		45	81		61																				
**RPFT-SB01-58.0-60.0**	17.98			59		43																				
**RPFT-SB01-60.0-64.0**	18.90			65		54							65													

Supplementary Table 15. **Soil PFAS in Field 2.** No analytes were detected at or above the reporting limit in equipment rinse blanks/field blanks. Note, blank cells represent non-detects.

Sample ID	Depth	PFBA	PFPeA	PFHxA	PFHpA	PFOA	PFNA	PFDA	PFUdA	PFDoA	PFTrDA	PFTeDA	PFBS	PFPeS	PFHxS	PFHpS	PFOS	PFNS	PFDS	4:2FTS	6:2FTS	8:2FTS	N-EtFOSAA	N-MeFOSAA	FOSA	HFPO-DA
Depth Increment (ft)	(m)	(ng/kg)	(ng/kg)	(ng/kg)	(ng/kg)	(ng/kg)	(ng/kg)	(ng/kg)	(ng/kg)	(ng/kg)	(ng/kg)	(ng/kg)	(ng/kg)	(ng/kg)	(ng/kg)	(ng/kg)	(ng/kg)	(ng/kg)	(ng/kg)	(ng/kg)	(ng/kg)	(ng/kg)	(ng/kg)	(ng/kg)	(ng/kg)	(ng/kg)
RPFT-SF201-0.0-0.5	0.08	120	270	320	460	2700	1300	7500	9800	11000	6600	4200			52		3500					240		330	180	
RPFT-SF202-0.0-0.5	0.08	240	370	400	420	1500	710	4500	9600	13000	8300	5900					2800					110		300	290	
RPFT-SF203-0.0-0.5	0.08	260	400	470	630	2700	1200	9400	12000	17000	11000	9000					3700					170		290	290	
RPFT-SF204-0.0-0.5	0.08	340	660	920	1100	4100	1400	7900	11000	15000	9000	6700			79		3300					220		320	340	
RPFT-SB02-0.0-0.5	0.08	430	610	570	620	2600	2100	18000	17000	15000	9600	4600					5000					130		240	240	
RPFT-SB02-0.5-1.0	0.23	340	530	580	620	3200	3000	25000	5700	2600	1700	1100					9200									
RPFT-SB02-1.0-1.5	0.38	900	1100	1300	1700	11000	9100	31000	1100	180	110				73		27000									
RPFT-SB02-1.5-2.0	0.53	640	930	1100	1400	13000	16000	1100	100	57					76	100	13000									
RPFT-SB02-2.0-2.5	0.69	630	1000	1100	1300	13000	4700	93							67	160	1900									
RPFT-SB02-2.5-3.0	0.84	540	980	1200	1300	16000	600								72	140	260									
RPFT-SB02-3.0-3.5	0.99	380	640	740	980	16000	200								74	86	120									
RPFT-SB02-3.5-4.0	1.14	410	590	720	1000	14000									70											
RPFT-SB02-4.5-5.0	1.45	490	770	1100	1100	7300																				
RPFT-SB02-5.0-6.0	1.68	240	430	680	940	7300				75	52				61											
RPFT-SB02-6.0-7.0	1.98	310	530	880	1300	9600									83											
RPFT-SB02-7.0-8.0	2.29	430	770	1300	1600	7400									96											
RPFT-SB02-9.0-10.0	2.90	530	910	1400	1600	4800		72	65	81	62				120											
RPFT-SB02-10.0-12.0	3.35	620	1300	2100	1800	1800									130											
RPFT-SB02-14.0-16.0	4.57	360	720	1100	890	1400																				
RPFT-SB02-18.0-20.0	5.79	180	420	690	560	1200							97		66											
RPFT-SB02-22.0-24.0	7.01		170	270	200	310							580													
RPFT-SB02-24.0-26.0	7.62		150	260	180	280							660													
RPFT-SB02-26.0-28.0	8.23		84	140	100	170							420													
RPFT-SB02-28.0-30.0	8.84																									
RPFT-SB02-30.0-32.0	9.45																									
RPFT-SB02-32.0-34.0	10.06																									
RPFT-SB02-34.0-36.0	10.67																									
RPFT-SB02-36.0-38.0	11.28																									
RPFT-SB02-38.0-40.0	11.89																									
RPFT-SB02-40.0-42.0	12.50																									
RPFT-SB02-42.0-44.0	13.11																									
RPFT-SB02-44.0-48.0	14.02																									
RPFT-SB02-48.0-50.0	14.94																									
RPFT-SB02-50.0-52.0	15.54																									
RPFT-SB02-52.0-54.0	16.15																									
RPFT-SB02-54.0-56.0	16.76																									
RPFT-SB02-56.0-58.0	17.37																									
RPFT-SB02-58.0-60.0	17.98																									
RPFT-SB02-60.0-62.0	18.59																									
RPFT-SB02-62.0-64.0	19.20																									
RPFT-SB02-64.0-66.0	19.81																									

Supplementary Table 16. **Groundwater PFAS in Field 1.** No analytes were detected at or above the reporting limit in the groundwater field blanks. Note, blank cells represent non-detects.

Sample ID	Depth (Sh/Dp)	PFBA (ng/L)	PFPeA (ng/L)	PFHxA (ng/L)	PFHpA (ng/L)	PFOA (ng/L)	PFNA (ng/L)	PFDA (ng/L)	PFUdA (ng/L)	PFDoA (ng/L)	PFTrDA (ng/L)	PFTeDA (ng/L)	PFBS (ng/L)	PFPeS (ng/L)	PFHxS (ng/L)	PFHpS (ng/L)	PFOS (ng/L)	PFNS (ng/L)	PFDS (ng/L)	4:2FTS (ng/L)	6:2FTS (ng/L)	8:2FTS (ng/L)	N-EtFOSAA (ng/L)	N-MeFOSAA (ng/L)	FOSA (ng/L)	HFPO-DA (ng/L)
**GW F1 Sh**	Shallow	960	2200	3900	2800	6300	630	65					12000	220	650	150	3400									
**GW F1 Dp**	Deep	16	43	92	53	70							68	3.9	9.8											
**GW F1 Dp Dup**	Deep	16	41	90	55	62							68	4.2	12											

Supplementary Table 17. **Groundwater PFAS in Field 2.** No analytes were detected at or above the reporting limit in the groundwater field blanks. Note, blank cells represent non-detects.

Sample ID	Depth (Sh/Dp)	PFBA (ng/L)	PFPeA (ng/L)	PFHxA (ng/L)	PFHpA (ng/L)	PFOA (ng/L)	PFNA (ng/L)	PFDA (ng/L)	PFUdA (ng/L)	PFDoA (ng/L)	PFTrDA (ng/L)	PFTeDA (ng/L)	PFBS (ng/L)	PFPeS (ng/L)	PFHxS (ng/L)	PFHpS (ng/L)	PFOS (ng/L)	PFNS (ng/L)	PFDS (ng/L)	4:2FTS (ng/L)	6:2FTS (ng/L)	8:2FTS (ng/L)	N-EtFOSAA (ng/L)	N-MeFOSAA (ng/L)	FOSA (ng/L)	HFPO-DA (ng/L)
**GW F2 Sh**	Shallow	270	560	1100	920	2200	72	0	0	0	0	0	71	30	99	14										
**GW F2 Dp**	Deep																									
**GW F2 Dp Dup**	Deep																									

Supplementary Table 18. **Surface water PFAS downgradient of Fields 1 & 2.** Anonymity of properties maintained in sample-location identification. Data limitations can be found in the EPA ScienceHub. Note, blank cells represent non-detects.

Sample ID	Field # (Note)	PFBA (ng/L)	PFPeA (ng/L)	PFHxA (ng/L)	PFHpA (ng/L)	PFOA (ng/L)	PFNA (ng/L)	PFDA (ng/L)	PFUdA (ng/L)	PFDoA (ng/L)	PFTrDA (ng/L)	PFTeDA (ng/L)	PFBS (ng/L)	PFPeS (ng/L)	PFHxS (ng/L)	PFHpS (ng/L)	PFOS (ng/L)	PFNS (ng/L)	PFDS (ng/L)	PFDoS (ng/L)	4:2FTS (ng/L)	6:2FTS (ng/L)	8:2FTS (ng/L)	N-EtFOSAA (ng/L)	N-MeFOSAA (ng/L)	FOSAA (ng/L)	N-MeFOSA (ng/L)	N-EtFOSE (ng/L)	N-EtFOSA (ng/L)
**SP01**	F1(1)	305	651	900	773	1700	397	110			7	4	2104	23	66	17	665							2	2				
**SP02**	F1(2)	319	705	1039	817	1751	251	27	2		8	3	2475	27	71	14	213												
**SP03**	F2(3)	19	38	112	35	71	5	5	2				148	2	4		5												
**SP04**	F2(4)	65	162	285	160	288					7	3	241	13	29	3	11							2					
**SP05**	F1 & F2(5)	26	67	120	52	90	7	5	2				100	3	7		5												

(1) Intermittent stream headwater surrounded by Field 1, ~98 m downgradient of Field 1 soil boring. Pooled and not flowing at the surface at time of sampling.
(2) Intermittent stream headwater surrounded by Field 1, ~180 m downgradient of SP01. Pooled and not flowing at the surface at time of sampling.
(3) Regional stream, ~430 m downgradient of Field 2 soil boring.
(4) Regional stream, ~1400 m flowing downstream of SP03.
(5) Regional stream, ~1200 m flowing downstream of SP04 and below confluence with stream fed by headwaters in Field 1.

III. Extended geochemical summary of soil weathering to produce authigenic minerals governing PFAS fate in the subsurface

In *incipient* weathering regimes primary rock minerals predominate initially, commonly including feldspars, micas, olivines, amphiboles, pyroxenes and still other accessory minerals [40,41]. Meteoric water bearing carbonic acid from reaction with atmospheric carbon dioxide and dissolved organic acids from natural organic matter, percolates into tortuous restricted-flow pore space of subsurface soils where microbial respiration of the dissolved organic matter consumes oxygen and increases carbonic acid still more to foster a complex variable-redox and acid-base system [23] that attacks these primary minerals fostering primary-mineral dissolution followed by precipitation of a narrowly limited variety of authigenic (formed in place) minerals that dominate the active subsurface flow regime, the precise assemblage of which is dictated by thermodynamic, kinetic and temporal constraints [42].

Incipient weathering produces secondary mineral phases including 2:1 (two silica tetrahedra sheets sandwiching an alumina octahedral sheet) layer-silicate clays such as illite, vermiculite and smectites (e.g., beidellite, montmorillonite), with commonly lesser amounts of 1:1 layer-silicate kaolinitic clays, and (oxy)hydroxide minerals of aluminum and ferric iron [40,41] (Figure 2a). As the primary minerals become depleted, during *intermediate* weathering, the secondary mineral phases predominate and are transitioning from weathering products to reactants, with the 2:1 phyllosilicates undergoing dissolution to produce tertiary authigenic minerals, dominantly 1:1 kaolinitic clays and commonly lesser amounts of (oxy)hydroxide minerals of aluminum and ferric iron [40-42] (Figure 2a). During the *advanced* weathering stage, 1:1 kaolinitic clays predominate and are transitioning from weathering products to reactants to produce terminal oxides of aluminum and ferric iron [40,43] (Figure 2, Supplementary Fig. 7). Finally during the *extreme* weathering phase, the chemically stable oxide minerals of aluminum, iron and silica predominate [43] (Figure 2b).

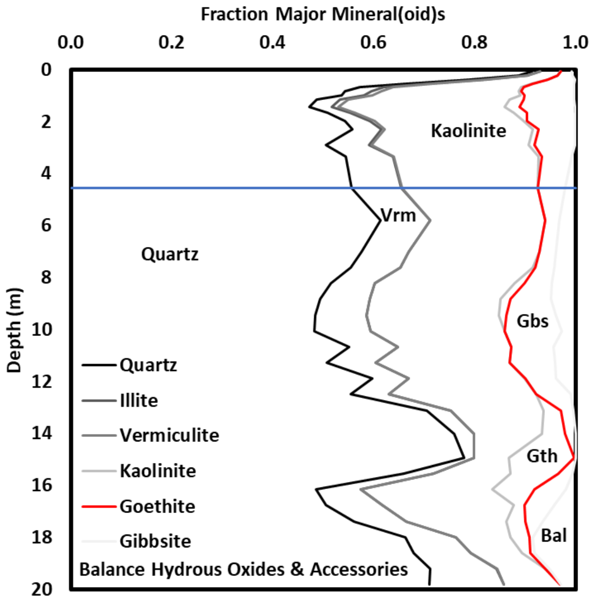

Supplementary Fig. 5. **Mineralogic profile using data from the current study, dominated by resistate quartz and authigenic kaolinite and gibbsite, are found in both the residual (depicted here, Field 2), and colluvial (Figure 2b, Field 1) profiles.** In both Figure 2b and Supplementary Fig. 5, the depicted curves are 3-point smoothed.

In stable soil profiles, these weathering regimes commonly vary with depth, incipient at the bedrock interface, perhaps grading to intermediate in the B horizons, and perhaps advanced in the upper-most A and E horizons (Figure 1). The weathering phases described here of the most well developed portions of the soil profile, the solum, are roughly reflected in several of the mapped USDA soil orders [44], with extreme weathering profiles commonly observed in the oxisols of Hawaii and Puerto Rico, advanced weathering profiles commonly being observed in the ultisols of Georgia and South Carolina for example, intermediate weathering profiles commonly being observed in the alfisols of Pennsylvania and Ohio, and incipient weathering commonly observed in entisols and inceptisols of Maine and Alaska.

The United States Department of Agriculture (USDA), National Cooperative Soil Survey (NCSS) has assembled perhaps the largest database of soil mineralogy globally, including geochemical and mineralogical data for more than 20,000 US soil pedons [15]. Soil mineralogy in this database is characterized by semi-quantitative X-ray diffraction of the clay-sized (<0.002 mm diameter) portion of the soils. We extracted these soil mineralogical data from this database, summarizing by Soil Order and soil horizon, so that each mineral-Soil Order-horizon commonly summarizes hundreds to thousands of samples (Table 1, Supplementary Tables 19-21). These tabulated summaries document the weathering patterns described above. For example, the 2:1 clays (vermiculite and montmorillonite) generally

decrease with increasing pedogenetic weathering, entisols → inceptisols → alfisols → ultisols → oxisols, and the 1:1 clay kaolinite and oxide minerals (goethite, hematite, gibbsite) of subsurface horizons generally increase with increasing pedogenesis. Assessing degree of weathering with increasing depth, in the intermediately weathered alfisols, the incipiently generated 2:1 clays (vermiculite, montmorillonite) are most intensely detected in the C horizon (Supplementary Table 21), followed by the B horizon (Supplementary Table 20), and least in the A horizon (Supplementary Table 19).

Supplementary Table 19. **Summary of USDA/NCSS mineralogy data by Soil Order in soil horizon A.**

A Horizon	Statistic	Increasing Pedogenesis						
Mineral(oid)	*(count)*	Entisol	Inceptisol	Mollisol (2)	Alfisol	Spodosol (2)	Ultisol	Oxisol
**Semi-quantitative X-ray proportion of clay-size fraction (1)**								
**Quartz**	$\bar{X} \pm \sigma$ *(n)*	1.2 ± 0.4 *(174)*	1.1 ± 0.3 *(214)*	1.1 ± 0.3 *(892)*	1.2 ± 0.4 *(621)*	1.0 ± 0.0 *(3)*	1.2 ± 0.4 *(174)*	2.3 ± 1.5 *(3)*
	Median	1.0	1.0	1.0	1.0	1.0	1.0	2.0
**Vermiculite**	$\bar{X} \pm \sigma$ *(n)*	1.3 ± 0.5 *(9)*	1.7 ± 0.9 *(38)*	1.7 ± 0.8 *(45)*	1.6 ± 0.7 *(83)*	1.5 ± 0.7 *(2)*	1.2 ± 0.4 *(21)*	N/A
	Median	1.0	2.0	2.0	2.0	1.5	1.0	N/A
**Montmorillonite**	$\bar{X} \pm \sigma$ *(n)*	2.7 ± 1.1 *(253)*	2.3 ± 1.0 *(199)*	2.5 ± 0.9 *(1300)*	2.2 ± 1.0 *(515)*	3.3 ± 1.0 *(4)*	1.8 ± 1.0 *(33)*	N/A
	Median	3.0	2.0	3.0	2.0	3.5	1.0	N/A
**Kaolinite**	$\bar{X} \pm \sigma$ *(n)*	2.1 ± 0.9 *(386)*	2.0 ± 0.9 *(543)*	1.9 ± 0.6 *(1547)*	2.3 ± 0.7 *(984)*	1.5 ± 0.8 *(11)*	2.9 ± 0.9 *(348)*	3.1 ± 1.1 *(59)*
	Median	2.0	2.0	2.0	2.0	1.0	3.0	3.0
**Goethite**	$\bar{X} \pm \sigma$ *(n)*	1.0 ± 0.0 *(7)*	1.3 ± 0.5 *(56)*	1.3 ± 0.5 *(44)*	1.2 ± 0.4 *(96)*	2.0 ± 0.0 *(2)*	1.3 ± 0.5 *(104)*	1.5 ± 0.5 *(60)*
	Median	1.0	1.0	1.0	1.0	2.0	1.0	2.0
**Hematite**	$\bar{X} \pm \sigma$ *(n)*	1.2 ± 0.4 *(14)*	1.3 ± 0.5 *(49)*	1.1 ± 0.3 *(33)*	1.3 ± 0.5 *(42)*	2.0 ± 0 *(2)*	1.4 ± 0.6 *(47)*	1.7 ± 0.6 *(42)*
	Median	1.0	1.0	1.0	1.0	2.0	1.0	2.0
**Gibbsite**	$\bar{X} \pm \sigma$ *(n)*	1.7 ± 1.6 *(3)*	1.4 ± 0.7 *(77)*	1.1 ± 0.4 *(8)*	1.3 ± 0.6 *(22)*	N/A	1.6 ± 0.6 *(108)*	2.0 ± 0.9 *(56)*
	Median	1.0	1.0	1.0	1.0	N/A	1.0	2.0
**Quantitative percent (geometric mean & standard deviation) of dry soil mass**								
**HFO**	$\bar{X}_g$ *(n)*	0.22 *(220)*	0.44 *(753)*	0.27 *(893)*	0.30 *(482)*	0.41 *(17)*	0.30 *(263)*	0.43 *(77)*
	σ_g range	0.08 - 0.60	0.17 - 1.15	0.11 - 0.71	0.12 - 0.76	0.11 - 1.50	0.11 - 0.82	0.19 - 0.97
	Median	0.25	0.48	0.30	0.34	0.75	0.31	0.44
**HAO**	$\bar{X}_g$ *(n)*	0.14 *(225)*	0.34 *(762)*	0.16 *(891)*	0.15 *(485)*	0.21 *(18)*	0.24 *(261)*	0.37 *(77)*
	σ_g range	0.05 - 0.37	0.13 - 0.90	0.07 - 0.36	0.06 - 0.38	0.05 - 0.89	0.10 - 0.59	0.21 - 0.66
	Median	0.25	0.48	0.30	0.34	0.75	0.31	0.44
**Quantitative percent (mean & standard deviation) of dry soil mass**								
**TOC**	$\bar{X} \pm \sigma$ *(n)*	2.7 ± 4.4 *(247)*	4.6 ± 4.3 *(640)*	2.6 ± 2.6 *(1015)*	2.1 ± 2.2 *(527)*	6.0 ± 2.5 *(4)*	3.8 ± 3.7 *(278)*	6.4 ± 3.9 *(26)*
	Median	1.4	3.4	1.9	1.4	4.7	2.9	5.5

(1) Semi-quantitative scale of proportion of clay-size fraction based upon X-ray peak height (cnts/s): 1=very small (<110); 2=small (110-360); 3=medium (360-1120); 4=large 1120-1800); 5=very large (>1800).

(2) Mollisol and Spodosol occur as a result of particular climatic and environmental conditions, not solely progressive weathering.

Supplementary Table 20. **Summary of USDA/NCSS mineralogy data by Soil Order in soil horizon B.**

B Horizon Mineral(oid)	Statistic *(count)*	Increasing Pedogenesis						
		Entisol	Inceptisol	Mollisol (2)	Alfisol	Spodosol (2)	Ultisol	Oxisol
**Semi-quantitative X-ray proportion of clay-size fraction (1)**								
**Quartz**	$\bar{X} \pm \sigma$ *(n)*	1.2 ± 0.5 *(131)*	1.1 ± 0.4 *(612)*	1.1 ± 0.3 *(2043)*	1.2 ± 0.4 *(2085)*	1.1 ± 0.3 *(60)*	1.2 ± 0.6 *(494)*	2.1 ± 1.3 *(22)*
	Median	1.0	1.0	1.0	1.0	1.0	1.0	4.0
**Vermiculite**	$\bar{X} \pm \sigma$ *(n)*	1.9 ± 0.8 *(9)*	1.9 ± 0.9 *(160)*	1.9 ± 0.7 *(117)*	2.0 ± 0.8 *(354)*	1.7 ± 0.5 *(9)*	1.7 ± 0.7 *(102)*	1.7 ± 0.6 *(3)*
	Median	2.0	2.0	2.0	2.0	2.0	1.0	2.0
**Montmorillonite**	$\bar{X} \pm \sigma$ *(n)*	2.6 ± 1.1 *(256)*	2.6 ± 1.1 *(572)*	2.9 ± 0.9 *(3092)*	2.8 ± 1.0 *(2749)*	1.8 ± 0.8 *(57)*	2.0 ± 1.0 *(228)*	1.1 ± 0.4 *(7)*
	Median	3.0	3.0	3.0	3.0	2.0	2.0	1.0
**Kaolinite**	$\bar{X} \pm \sigma$ *(n)*	2.2 ± 1.0 *(320)*	2.3 ± 1.0 *(1445)*	2.1 ± 0.7 *(3434)*	2.7 ± 0.8 *(3894)*	1.8 ± 0.8 *(157)*	3.3 ± 1.0 *(1503)*	3.3 ± 1.0 *(264)*
	Median	2.0	2.0	2.0	3.0	2.0	3.0	3.0
**Goethite**	$\bar{X} \pm \sigma$ *(n)*	1.0 ± 0.2 *(24)*	1.3 ± 0.5 *(266)*	1.3 ± 0.5 *(178)*	1.4 ± 0.5 *(893)*	1.5 ± 0.7 *(15)*	1.4 ± 0.5 *(956)*	1.7 ± 0.6 *(282)*
	Median	1.0	1.0	1.0	1.0	2.0	1.0	2.0
**Hematite**	$\bar{X} \pm \sigma$ *(n)*	1.2 ± 0.4 *(6)*	1.2 ± 0.4 *(110)*	1.1 ± 0.3 *(134)*	1.1 ± 0.3 *(422)*	1.1 ± 0.3 *(18)*	1.3 ± 0.5 *(475)*	1.7 ± 0.6 *(146)*
	Median	1.0	1.0	1.0	1.0	1.0	1.0	2.0
**Gibbsite**	$\bar{X} \pm \sigma$ *(n)*	1.7 ± 0.9 *(15)*	1.8 ± 0.7 *(283)*	1.4 ± 0.6 *(27)*	1.5 ± 0.7 *(135)*	1.6 ± 0.5 *(13)*	2.0 ± 0.8 *(571)*	1.9 ± 0.8 *(187)*
	Median	2.0	2.0	1.0	1.0	2.0	2.0	2.0
**Quantitative percent (geometric mean & standard deviation) of dry soil mass**								
**HFO**	$\bar{X}_g$ *(n)*	0.13 *(184)*	0.37 *(1758)*	0.20 *(1551)*	0.24 *(1664)*	0.48 *(253)*	0.19 *(1171)*	0.25 *(248)*
	σ_g range	0.05 - 0.37	0.13 - 1.08	0.06 - 0.64	0.09 - 0.67	0.14 - 1.64	0.06 - 0.62	0.09 - 0.72
	Median	0.14	0.40	0.22	0.27	0.64	0.21	0.26
**HAO**	$\bar{X}_g$ *(n)*	0.12 *(189)*	0.24 *(1769)*	0.13 *(1564)*	0.13 *(1664)*	0.58 *(271)*	0.18 *(1174)*	0.29 *(250)*
	σ_g range	0.04 - 0.34	0.08 - 0.74	0.06 - 0.29	0.06 - 0.26	0.16 - 2.18	0.08 - 0.38	0.16 - 0.51
	Median	0.14	0.40	0.22	0.27	0.64	0.21	0.26
**Quantitative percent (mean & standard deviation) of dry soil mass**								
**TOC**	$\bar{X} \pm \sigma$ *(n)*	0.58 ± 0.72 *(268)*	0.91 ± 1.0 *(1666)*	0.68 ± 0.68 *(2412)*	0.33 ± 0.30 *(2379)*	2.8 ± 2.6 *(142)*	0.40 ± 0.47 *(1424)*	1.5 ± 0.9 *(69)*
	Median	0.3	0.6	0.5	0.3	2.1	0.3	1.5

(1) Semi-quantitative scale of proportion of clay-size fraction based upon X-ray peak height (cnts/s): 1=very small (<110); 2=small (110-360); 3=medium (360-1120); 4=large 1120-1800); 5=very large (>1800).

(2) Mollisol and Spodosol occur as a result of particular climatic and environmental conditions, not solely progressive weathering.

Supplementary Table 21. **Summary of USDA/NCSS mineralogy data by Soil Order in soil horizon C.**

C Horizon Mineral(oid)	Statistic *(count)*	Increasing Pedogenesis						
		**Entisol**	**Inceptisol**	**Mollisol (2)**	**Alfisol**	**Spodosol (2)**	**Ultisol**	**Oxisol**
**Semi-quantitative X-ray proportion of clay-size fraction (1)**								
**Quartz**	$\bar{X} \pm \sigma$ *(n)*	1.1 ± 0.4 *(296)*	1.1 ± 0.4 *(153)*	1.1 ± 0.3 *(415)*	1.2 ± 0.4 *(288)*	1.0 ± 0.0 *(1)*	1.2 ± 0.6 *(52)*	1.0 ± 0.0 *(1)*
	Median	1.0	1.0	1.0	1.0	1.0	1.0	1.0
**Vermiculite**	$\bar{X} \pm \sigma$ *(n)*	1.7 ± 0.6 *(32)*	1.7 ± 0.8 *(46)*	1.9 ± 0.7 *(21)*	2.1 ± 0.7 *(42)*	1.3 ± 0.6 *(3)*	1.4 ± 0.5 *(22)*	NA
	Median	1.5	2.0	2.0	2.0	1.5	1.0	NA
**Montmorillonite**	$\bar{X} \pm \sigma$ *(n)*	2.9 ± 1.1 *(418)*	2.4 ± 1.1 *(192)*	3.1 ± 1.0 *(688)*	3.0 ± 1.1 *(404)*	2.4 ± 1.1 *(13)*	2.7 ± 1.2 *(63)*	2.5 ± 0.7 *(2)*
	Median	3.0	2.0	3.0	3.0	2.0	3.0	2.5
**Kaolinite**	$\bar{X} \pm \sigma$ *(n)*	2.2 ± 1.0 *(564)*	2.4 ± 1.0 *(488)*	2.1 ± 0.8 *(725)*	2.4 ± 0.9 *(576)*	1.9 ± 0.8 *(45)*	3.1 ± 1.1 *(207)*	3.3 ± 1.3 *(24)*
	Median	2.0	2.0	2.0	2.0	2.0	3.0	3.0
**Goethite**	$\bar{X} \pm \sigma$ *(n)*	1.2 ± 0.4 *(32)*	1.3 ± 0.5 *(66)*	1.1 ± 0.3 *(42)*	1.5 ± 0.5 *(141)*	2.0 ± 0.0 *(2)*	1.4 ± 0.5 *(121)*	1.4 ± 0.7 *(23)*
	Median	1.0	1.0	1.0	1.0	2.0	1.0	1.0
**Hematite**	$\bar{X} \pm \sigma$ *(n)*	1.3 ± 0.5 *(18)*	1.3 ± 0.5 *(38)*	1.0 ± 0.2 *(26)*	1.1 ± 0.4 *(64)*	1.0 ± 0.0 *(5)*	1.3 ± 0.5 *(35)*	2.0 ± 0.6 *(6)*
	Median	1.0	1.0	1.0	1.0	1.0	1.0	2.0
**Gibbsite**	$\bar{X} \pm \sigma$ *(n)*	1.5 ± 0.5 *(10)*	1.8 ± 0.9 *(69)*	1.5 ± 0.6 *(4)*	1.5 ± 0.5 *(13)*	1.5 ± 0.7 *(2)*	1.8 ± 0.8 *(71)*	1.5 ± 0.6 *(16)*
	Median	1.5	1.0	1.5	1.0	1.5	2.0	1.0
**Quantitative percent (geometric mean & standard deviation) of dry soil mass**								
**HFO**	$\bar{X}_g$ *(n)*	0.18 *(281)*	0.20 *(526)*	0.14 *(332)*	0.13 *(227)*	0.18 *(70)*	0.11 *(145)*	0.35 *(26)*
	σ_g range	0.05 - 0.63	0.06 - 0.70	0.04 - 0.44	0.04 - 0.39	0.07 - 0.45	0.03 - 0.34	0.07 - 1.69
	Median	0.23	0.19	0.16	0.13	0.15	0.13	0.23
**HAO**	$\bar{X}_g$ *(n)*	0.07 *(285)*	0.12 *(528)*	0.07 *(334)*	0.06 *(237)*	0.16 *(75)*	0.09 *(152)*	0.28 *(28)*
	σ_g range	0.03 - 0.19	0.04 - 0.38	0.03 - 0.18	0.02 - 0.15	0.05 - 0.53	0.04 - 0.23	0.13 - 0.60
	Median	0.23	0.19	0.16	0.13	0.15	0.13	0.23
**Quantitative percent (mean & standard deviation) of dry soil mass**								
**TOC**	$\bar{X} \pm \sigma$ *(n)*	0.89 ± 2.0 *(521)*	0.53 ± 0.73 *(499)*	0.37 ± 0.69 *(394)*	0.27 ± 0.64 *(255)*	0.55 ± 0.31 *(42)*	0.14 ± 0.18 *(133)*	0.2 ± NA *(2)*
	Median	0.3	0.3	0.2	0.1	0.6	0.1	0.2

(1) Semi-quantitative scale of proportion of clay-size fraction based upon X-ray peak height (cnts/s): 1=very small (<110); 2=small (110-360); 3=medium (360-1120); 4=large 1120-1800); 5=very large (>1800).

(2) Mollisol and Spodosol occur as a result of particular climatic and environmental conditions, not solely progressive weathering.

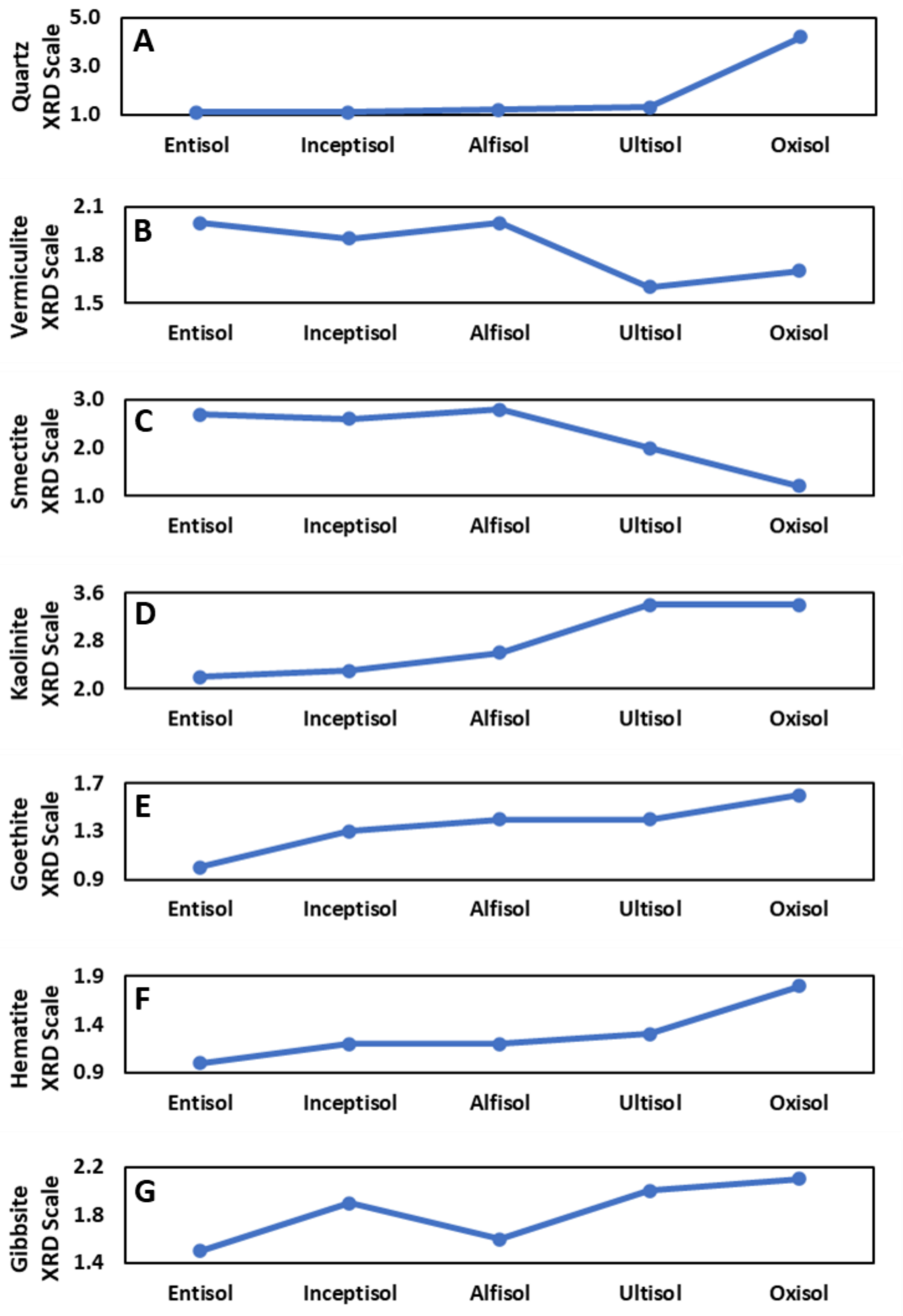

Supplementary Fig. 6. **USDA/NCSS B-horizon, x-ray diffraction (XRD) scale mean values (from Table 1 and Supplementary Table 20) vs Soil Order for the minerals quartz (a) through gibbsite (g).** The smectite mineral (**c**) is montmorillonite. Soil Orders are listed in order of increasing weathering. In general, with increasing weathering, the mineral XRD scales depict i) a downward trend in the 2:1 clays (vermiculite, smectite-montmorillonite) and ii) upward trends in the 1:1 clay (kaolinite) and the (hydr)oxide minerals (goethite, hematite, gibbsite).

Because of commonly high surface free energy for thermodynamically stable mineral phases, metastable amorphous phases (mineraloids) tend to nucleate as intermediates heterogeneously on existing surfaces as transient intermediates in the production of stable authigenic minerals [45]. With these amorphous phases being thermodynamically and kinetically favored, as each mineral assemblage weathers to form the next, a quasi-steady state of these transitional mineraloid phases persist and, hence, are nearly ubiquitously distributed in weathering environments [45,46]. While the amorphous interior of these intermediates imposes a higher lattice free energy than the repeated unit-cell interior of their more stable crystalline counterparts, the surface free energy (i.e., surface tension) of these amorphous phases is roughly an order of magnitude lower than common crystalline minerals and, because the free energy of nucleation is third-order dependent on surface tension [45], formation of these transient phases is favored in weathering settings. After these amorphous particles nucleate, their surfaces act as templates for precipitation of the crystalline mineral phases, diminishing the free-energy activation barrier to nucleation and subsequently the crystalline mineral nuclei slowly grow by cannibalizing the amorphous solids in a process known as Ostwald ripening [45].

Prominent among these amorphous phases, hydrous aluminum oxide (HAO with a formula roughly of $Al(OH)_3$) grading to short-range-ordered gibbsite and hydrous ferric oxide (HFO, roughly $Fe(OH)_3$) grading to short-range-ordered ferrihydrite, have surface areas on the order of ten-fold higher than kaolinitic clay-size particles, and they bear pH- and ionic-strength- dependent electrostatic surface charges that offer rich sorption complexes for charged compounds. HAO and HFO were characterized in the USDA/NCSS database [15] using a selective extraction with ammonium oxalate and we document their ubiquitous distribution in US soils in Supplementary Tables 19-21.

Amorphous HAO and HFO share with many of their crystalline oxide phases (e.g., goethite, hematite, ferrihydrite, gibbsite) a reversible lability, wherein small changes in oxidation state (for Fe) and pH (for Fe and Al) can cause dissolution and precipitation (preferentially precipitating first the amorphous phases as described above). Authigenic ferric oxides tend to equilibrate with low activities of Fe^{2+} so that increasing pH and oxidation state (as Eh for example) fall in the stability field for these solids, whilst lower pH and Eh favor dissolved Fe, Fe^{2+} at moderately low pH, and Fe^{3+} at pH ranges of acidic mine drainage (Supplementary Fig. 7). Whilst Al oxides are insensitive to oxidation state, their solubility is a sensitive function of pH (Supplementary Fig. 8). And Nordstrom and Ball [24] report that acidified natural waters, falling in the range of the subsurface soils of our study sites, tend to equilibrate in a field delimited by amorphous $Al(OH)_3$ and microcrystalline gibbsite (Supplementary Fig. 8).

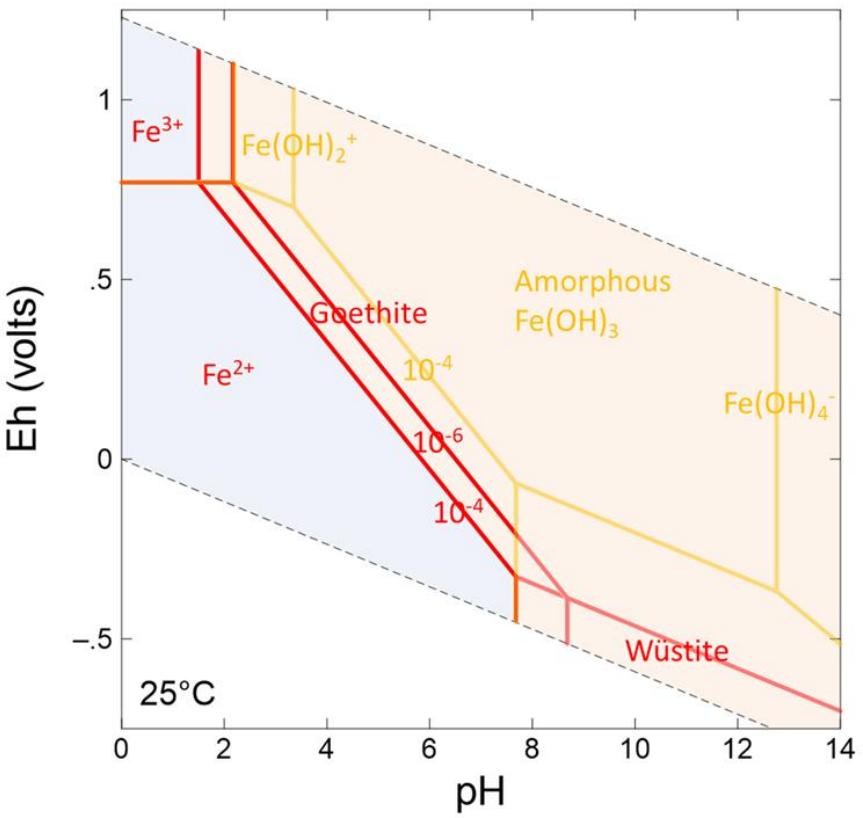

Supplementary Fig. 7. **Pourbaix (Eh-pH) diagram depicting stability fields for goethite and amorphous $Fe(OH)_3$ at selected molar activities in oxidation potential vs pH space**. Calculations for 25 °C, 1 bar and saturated with water, using thermodynamic dataset 'thermo.tdat' in Geochemist's Workbench [47] for aqueous speciation, goethite and amorphous $Fe(OH)_3$.

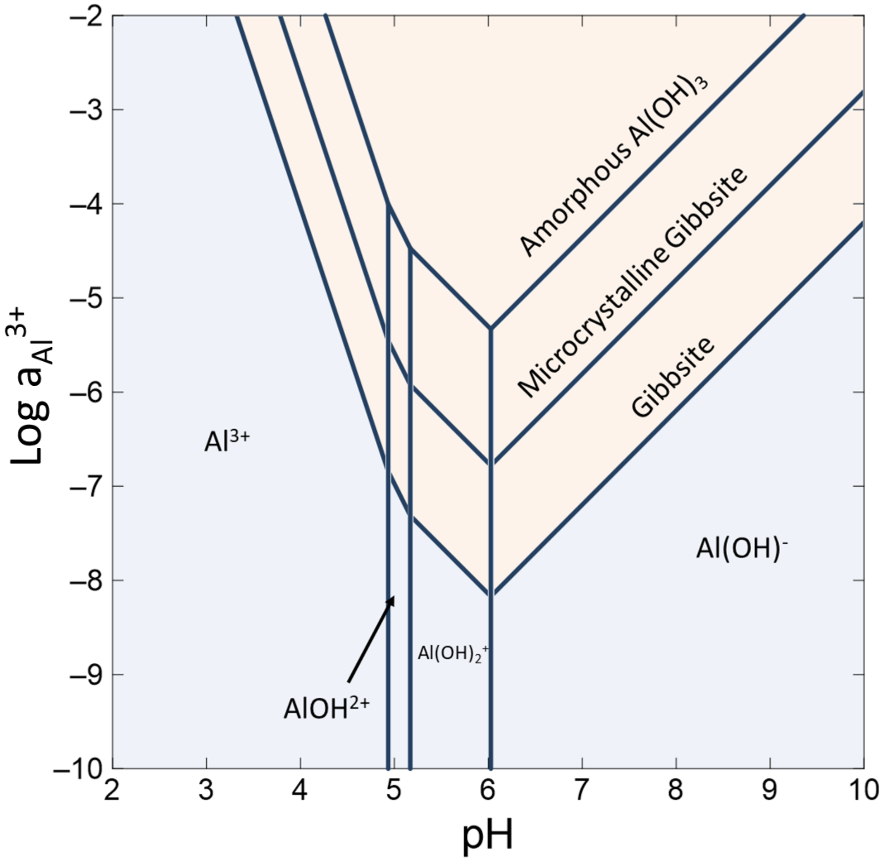

Supplementary Fig. 8. **Thermodynamic speciation diagram for aluminum. Calculations for 25 ºC, 1 bar and saturated with water, using thermodynamic dataset 'thermo.tdat' in Geochemist's Workbench [47] for aqueous speciation and gibbsite, and adding thermodynamic data for amorphous $Al(OH)_3$ and microcrystalline gibbsite as reported in Nordstrom et al. [48].**

The kinetics of precipitation of Fe and Al oxides as a function of [OH^-], likely impacts the ratio of amorphous to crystalline with higher pHs being conducive to faster reaction rates and less time for crystallization of amorphous solids to crystalline phases, as depicted in Figure 4 for colluvial Field 1 and Supplementary Fig. 9 residual Field 2.

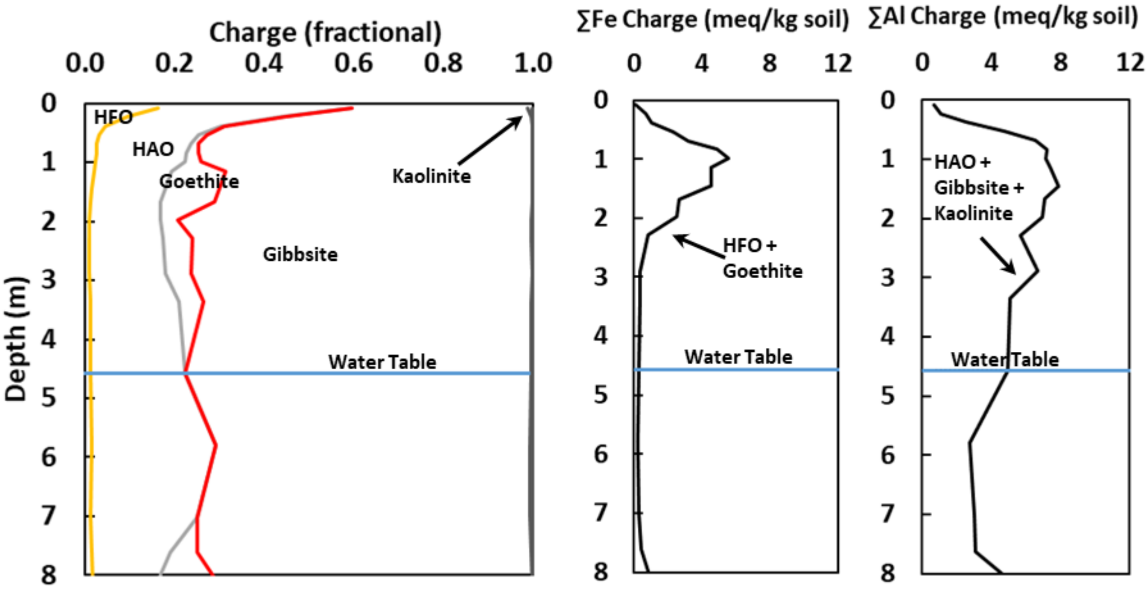

Supplementary Fig. 9. **Electrostatic charge distribution in the residuum soil column of Field 2.** Like in the colluvial soil of Field 1 (Figure 4), gibbsite constitutes the largest part of charge through much of the soil column.

Modeling the effect fostering the high amorphous/crystalline distribution of our study sites (Figure 4, Supplementary Fig. 9), Singer and Stumm [49] characterized the rate of Fe^{2+} oxidation by dissolved O_2, a necessary step in the formation of $Fe(OH)_3$ solids, according to:

$$-\frac{d[Fe^{2+}]}{dt} \simeq k[Fe^{2+}][O_2][OH^-]^2 + k'[Fe^{2+}][O_2] \tag{23}$$

Rearranging for quasi-steady state conditions, wherein O_2 and pH are held roughly constant:

$$\frac{d[Fe^{2+}]}{[Fe^{2+}]} \simeq -\{k[O_2][OH^-]^2 + k'[O_2]\}dt \tag{24}$$

And integrating:

$$\int \frac{d[Fe^{2+}]}{[Fe^{2+}]} \simeq -\{k[O_2][OH^-]^2 + k'[O_2]\} \int dt \tag{25}$$

$$ln \frac{[Fe^{2+}]_t}{[Fe^{2+}]_0} \simeq -\{k[O_2][OH^-]^2 + k'[O_2]\}\Delta t \tag{26}$$

Rearranging and solving for half-life:

$$T_{1/2} \simeq \frac{ln2}{[O_2][k[OH^-]^2+k']} \qquad (27)$$

Singer and Stumm [49] report values of $k = 8.0 \times 10^{13}$ $L^2/(m^2$ atm min) and $k' = 1.0 \times 10^{-7}$ 1/(atm min), $[OH^-]$ is specified by designating pH, and $[O_2]$ is taken as in equilibrium with the atmosphere, 0.208 atmospheres. From this, the half-life of ferrous oxidation by oxygen at $P_{O2} = 0.208$ atm, is shown in Supplementary Fig. 10.

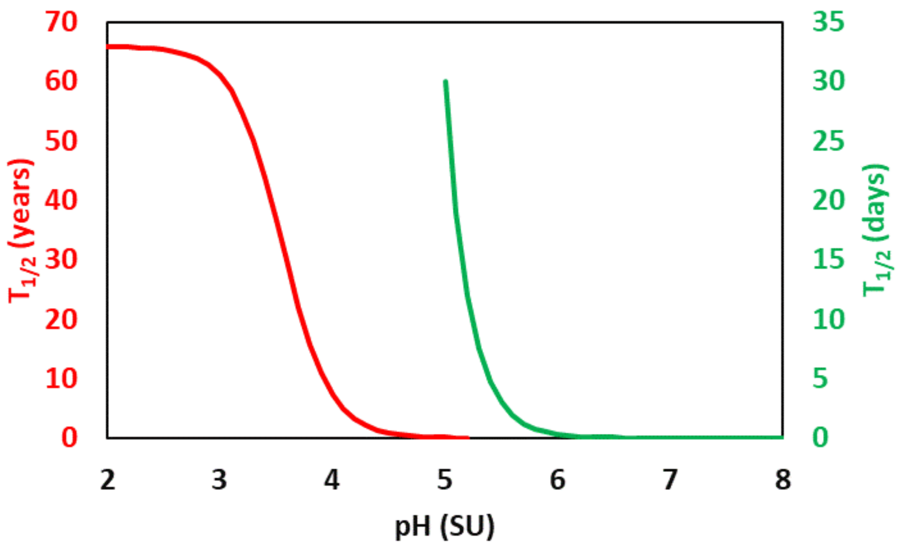

Supplementary Fig. 10. **Half-life of ferrous oxidation by O_2 at P_{O2} = 0.208 atm.** The pH of surface soils at our Field 1 and Field 2 study sites ranged from 7.62 to 5.05, equating to ferrous oxidation half-lives of 15 sec to 24 days, and plotting on the left end of the green curve above. The pH of subsurface soils at our study sites ranged from 5.03 to 4.16, equating to ferrous oxidation half-lives of 16 days to 3.7 years, and plotting on the right end of the red curve above. The fast oxidation rate of the surface soils may be conducive to precipitation of amorphous solids while the relatively slower oxidation rates of the subsurface conditions may render crystal formation relatively more favorable.

For the constants reported by Singer and Stumm, the half-life for Fe^{2+} oxidation at pH > 5 is days to a month in scale but at pH < 5, the oxidation half-life ascends quickly from years to decades with decreasing pH (Supplementary Fig. 10). For gibbsite, Nordstrom and Ball [24] report equilibrium is achieved within two hours at near neutral pH and Benezeth et al. [50] report reaction rate is first-order in $[OH^-]$ at moderate pHs, so that $Al(OH)_3$ precipitation will proceed more slowly at lower pH values.

Taken together, a higher fraction Fe and Al solids might be expected to be amorphous or poorly ordered at moderate pHs where kinetics proceed relatively more quickly than at lower pHs.

Taken as a group, these authigenic mineral(oid)s, including smectite clays, kaolinite clays, illite, vermiculite, crystalline Fe oxides (goethite, hematite, ferrihydrite), gibbsite, and the amorphous HFO and HAO, i) comprise a considerable portion of subsurface soils, sediments and weathered, high-flow zones in aquifers, ii) possess high interfacial areas with bulk waters, iii) commonly bear considerable permanent or pH-dependent surface charges, and iv) commonly have some capacity to form inner-sphere complexes with aqueous solutes. These traits all potentially contribute to regulating the mobility of ionized organic compounds in subsurface settings.

Supplementary Fig. 11. **Heat map of nominal correlations amongst geochemical properties for Field 1.**

Transformed Organic (%)		Aluminum (Oxy-)Hydroxide Mineral(oid)s				Ferric (Oxy-)Hydroxide Mineral(oid)s					Clay Fraction				Silt & Sand Fraction				Exchange Complex				Hydrostatic State				Mehlich Metals						Soluble Anions			
TOC	Total N	Extn Hydrous Al Oxide Conc	Extn Hydrous Al Oxide Charge	X-ray Gibbsite Conc.	X-ray Gibbsite Charge	Extn Hydrous Fe Oxide Conc	Extn Hydrous Fe Oxide Charge	X-ray Goethite Conc.	X-ray Goethite Charge	LogSum HF/HAO Goeth/ Gibbs Charge	X-ray Illite Conc	X-ray Kaolinite Conc	X-ray Kaolinite Charge	Textural Clay	Textural Silt	Textural Sand	X-ray Quartz Conc.	Extn Hydrous Mn Oxide Conc	Cation Exchange Capacity	Base Saturation	Lime Buffer Capacity	Sample Depth	Estimated Hydrostatic Pressure	Estimated Fraction Saturation	Measured Solids Content	Soil pH	LogMehlich Ca	LogMehlich Mg	LogMehlich Na	LogMehlich K	LogMehlich Fe	LogMehlich Pb	LogP	LogCl	LogNO3	Correl
1.00	0.92	0.40	-0.41	-0.36	-0.46	0.92	-0.09	-0.17	-0.24	-0.71	-0.74	-0.48	-0.54	-0.27	0.63	0.11	0.49	0.91	0.17	0.67	-0.25	-0.65	-0.65	-0.90	0.86	0.82	0.93	0.82	-0.06	0.64	0.50	-0.40	0.93	-0.50	0.71	TOC
	1.00	0.31	-0.40	-0.41	-0.46	0.84	-0.12	-0.22	-0.27	-0.70	-0.68	-0.47	-0.49	-0.31	0.60	0.15	0.50	0.89	0.18	0.64	-0.23	-0.55	-0.55	-0.86	0.76	0.76	0.83	0.71	-0.14	0.64	0.45	-0.44	0.89	-0.40	0.69	Total N
		1.00	0.45	0.60	0.33	0.46	0.19	0.41	0.26	0.21	0.02	0.54	0.19	0.73	0.75	-0.82	-0.53	0.14	0.55	0.07	0.59	-0.91	-0.91	-0.59	0.63	0.38	0.51	0.54	0.62	0.58	0.45	0.59	0.16	-0.17	-0.06	Extn Hydrous Al Oxide Conc
			1.00	0.68	0.90	-0.37	0.53	0.31	0.47	0.78	0.74	0.81	0.93	0.72	0.25	-0.71	-0.78	-0.43	0.72	-0.73	0.89	-0.25	-0.25	0.16	-0.21	-0.62	-0.43	-0.23	0.68	0.26	-0.04	0.76	-0.47	0.48	-0.46	Extn Hydrous Al Oxide Charge
				1.00	0.80	-0.25	0.30	0.54	0.44	0.81	0.66	0.92	0.60	0.94	0.25	-0.91	-0.95	-0.58	0.39	-0.38	0.73	-0.37	-0.37	0.21	0.00	-0.24	-0.20	-0.11	0.59	0.07	0.08	0.84	-0.57	0.18	-0.63	X-ray Gibbsite Conc.
					1.00	-0.41	0.53	0.28	0.42	0.84	0.82	0.82	0.93	0.71	0.19	-0.68	-0.81	-0.49	0.63	-0.69	0.81	-0.15	-0.15	0.33	-0.24	-0.63	-0.48	-0.33	0.56	0.18	-0.06	0.71	-0.52	0.50	-0.52	X-ray Gibbsite Charge
						1.00	0.13	-0.08	-0.18	-0.60	-0.69	-0.41	-0.53	-0.20	0.63	0.04	0.40	0.80	0.13	0.63	-0.17	-0.65	-0.65	-0.83	0.84	0.79	0.90	0.76	-0.02	0.55	0.57	-0.25	0.84	-0.54	0.61	Extn Hydrous Fe Oxide Conc
							1.00	0.25	0.43	0.42	0.28	0.29	0.48	0.26	0.23	-0.29	-0.32	-0.11	0.53	-0.43	0.60	-0.14	-0.14	0.04	-0.07	-0.42	-0.14	-0.03	0.50	0.12	0.17	0.43	-0.14	0.05	-0.05	Extn Hydrous Fe Oxide Charge
								1.00	0.91	0.53	0.09	0.48	0.15	0.64	-0.01	-0.58	-0.61	-0.37	0.26	-0.16	0.53	-0.32	-0.32	-0.04	0.17	-0.05	0.00	0.14	0.68	-0.21	0.02	0.50	-0.41	-0.36	-0.36	X-ray Goethite Conc.
									1.00	0.59	0.18	0.42	0.36	0.52	-0.05	-0.46	-0.55	-0.32	0.44	-0.38	0.62	-0.21	-0.21	0.03	0.04	-0.31	-0.19	0.01	0.72	-0.15	-0.08	0.44	-0.41	-0.20	-0.29	X-ray Goethite Charge
										1.00	0.83	0.86	0.80	0.74	-0.09	-0.66	-0.89	-0.80	0.33	-0.71	0.74	0.05	0.05	0.53	-0.39	-0.70	-0.63	-0.48	0.51	-0.23	-0.34	0.80	-0.82	0.39	-0.74	LogSum HF/HAO Goeth/ Gibbs Charge
											1.00	0.77	0.86	0.55	-0.18	-0.46	-0.74	-0.74	0.22	-0.67	0.53	0.25	0.25	0.66	-0.57	-0.76	-0.76	-0.69	0.21	-0.16	-0.28	0.60	-0.75	0.71	-0.70	X-ray Illite Conc
												1.00	0.76	0.93	0.19	-0.88	-0.98	-0.63	0.45	-0.55	0.80	-0.28	-0.28	0.25	-0.13	-0.40	-0.35	-0.19	0.61	0.08	-0.03	0.86	-0.63	0.35	-0.67	X-ray Kaolinite Conc
													1.00	0.57	0.08	-0.53	-0.71	-0.49	0.62	-0.80	0.77	0.00	0.00	0.37	-0.38	-0.77	-0.61	-0.44	0.48	0.15	-0.18	0.63	-0.53	0.64	-0.51	X-ray Kaolinite Charge
														1.00	0.32	-0.98	-0.94	-0.51	0.48	-0.37	0.79	-0.51	-0.51	0.04	0.09	-0.16	-0.10	0.05	0.71	0.16	0.13	0.87	-0.51	0.11	-0.57	Textural Clay
															1.00	-0.51	-0.14	0.49	0.54	0.12	0.39	-0.83	-0.83	-0.69	0.64	0.42	0.61	0.59	0.30	0.84	0.37	0.34	0.51	-0.06	0.36	Textural Silt
																1.00	0.89	0.35	-0.55	0.31	-0.81	0.65	0.65	0.11	-0.22	0.05	-0.04	-0.17	-0.71	-0.33	-0.19	-0.87	0.35	-0.09	0.43	Textural Sand
																	1.00	0.67	-0.42	0.52	-0.80	0.27	0.27	-0.27	0.12	0.39	0.35	0.20	-0.65	0.01	0.04	-0.86	0.68	-0.26	0.70	X-ray Quartz Conc.
																		1.00	0.17	0.56	-0.33	-0.44	-0.44	-0.82	0.71	0.66	0.77	0.68	-0.16	0.64	0.39	-0.59	0.98	-0.37	0.83	Extn Hydrous Mn Oxide Conc
																			1.00	-0.44	0.83	-0.58	-0.58	-0.38	0.31	-0.17	0.11	0.32	0.74	0.64	0.15	0.38	0.12	0.05	0.07	Cation Exchange Capacity
																				1.00	-0.64	-0.22	-0.22	-0.52	0.58	0.83	0.71	0.50	-0.35	0.04	0.41	-0.57	0.61	-0.53	0.47	Base Saturation
																					1.00	-0.45	-0.45	-0.03	0.03	-0.41	-0.19	0.03	0.82	0.30	0.00	0.78	-0.37	0.16	-0.35	Lime Buffer Capacity
																						1.00	1.00	0.80	-0.82	-0.56	-0.73	-0.77	-0.56	-0.72	-0.47	-0.35	-0.45	0.33	-0.21	Sample Depth
																							1.00	0.80	-0.82	-0.56	-0.73	-0.77	-0.56	-0.72	-0.47	-0.35	-0.45	0.33	-0.21	Estimated Hydrostatic Pressure
																								1.00	-0.89	-0.75	-0.89	-0.88	-0.26	-0.68	-0.45	0.21	-0.82	0.49	-0.61	Estimated Fraction Saturation
																									1.00	0.81	0.90	0.85	0.24	0.59	0.43	-0.14	0.73	-0.60	0.49	Measured Solids Content
																										1.00	0.92	0.77	-0.16	0.36	0.46	-0.37	0.72	-0.62	0.50	Soil pH
																											1.00	0.92	0.06	0.52	0.51	-0.27	0.82	-0.65	0.60	LogMehlich Ca
																												1.00	0.33	0.56	0.45	-0.17	0.70	-0.70	0.57	LogMehlich Mg
																													1.00	0.23	0.16	0.57	-0.21	-0.19	-0.18	LogMehlich Na
																														1.00	0.39	0.09	0.61	0.04	0.42	LogMehlich K
																															1.00	-0.02	0.41	-0.22	0.23	LogMehlich Fe
																																1.00	-0.58	0.30	-0.59	LogMehlich Pb
																																	1.00	-0.37	0.80	LogP
																																		1.00	-0.42	LogCl
																																			1.00	LogNO3

Positive correlation, Nominal P = 0.01

Positive correlation, Nominal P = 0.05

Positive correlation, Nominal P = 0.10

Negative correlation, Nominal P = 0.10

Negative correlation, Nominal P = 0.05

Negative correlation, Nominal P = 0.01

817 Supplementary Fig. 12. **Heat map of nominal correlations amongst geochemical properties for Field 2.**

Log Transformed Organic (%)		Aluminum (Oxy-)Hydroxide Mineral(oid)s				Ferric (Oxy-)Hydroxide Mineral(oid)s					Clay Fraction				Silt & Sand Fraction				Exchange Complex				Hydrostatic State				Mehlich Metals						Soluble Anions			
TOC	Total N	Extn Hydrous Al Oxide Conc	Extn Hydrous Al Oxide Charge	X-ray Gibbsite Conc.	X-ray Gibbsite Charge	Extn Hydrous Fe Oxide Conc	Extn Hydrous Fe Oxide Charge	X-ray Goethite Conc.	X-ray Goethite Charge	LogSum HF/HAO Goeth/ Gibbs Charge	X-ray Illite Conc	X-ray Kaolinite Conc	X-ray Kaolinite Charge	Textural Clay	Textural Silt	Textural Sand	X-ray Quartz Conc.	Extn Hydrous Mn Oxide Conc	Cation Exchange Capacity	Base Saturation	Lime Buffer Capacity	Sample Depth	Estimated Hydrostatic Pressure	Estimated Fraction Saturation	Measured Solids Content	Soil pH	LogMehlich Ca	LogMehlich Mg	LogMehlich Na	LogMehlich K	LogMehlich Fe	LogMehlich Pb	LogP	LogCl	LogNO3	Parameter Axis
1.00	0.84	-0.04	-0.49	-0.46	-0.59	0.81	0.44	-0.18	-0.18	-0.67	-0.83	-0.81	-0.86	-0.67	0.69	0.51	0.82	0.89	-0.71	0.91	-0.75	-0.71	-0.71	-0.76	0.75	0.86	0.87	-0.49	-0.60	0.47	0.92	0.41	0.90	-0.85	0.07	TOC
	1.00	-0.27	-0.53	-0.47	-0.53	0.54	0.20	-0.18	-0.17	-0.60	-0.63	-0.68	-0.67	-0.66	0.45	0.59	0.70	0.84	-0.43	0.66	-0.47	-0.51	-0.51	-0.61	0.56	0.64	0.58	-0.55	-0.27	0.06	0.73	0.23	0.82	-0.61	0.28	Total N
		1.00	0.82	0.79	0.67	0.41	0.68	0.29	0.28	0.61	-0.10	0.42	0.25	0.65	0.12	-0.76	-0.42	-0.30	0.11	-0.05	0.07	-0.47	-0.47	-0.27	0.33	-0.13	0.31	0.78	-0.51	0.49	0.06	0.33	-0.38	0.02	-0.22	Extn Hydrous Al Oxide Conc
			1.00	0.89	0.91	-0.07	0.40	0.44	0.43	0.87	0.34	0.80	0.72	0.93	-0.29	-0.94	-0.80	-0.69	0.60	-0.57	0.56	-0.06	-0.06	0.19	-0.11	-0.63	-0.19	0.91	-0.04	0.08	-0.32	0.16	-0.72	0.53	-0.01	Extn Hydrous Al Oxide Charge
				1.00	0.96	-0.10	0.29	0.43	0.42	0.92	0.28	0.77	0.66	0.86	-0.22	-0.88	-0.79	-0.61	0.49	-0.48	0.49	-0.07	-0.07	0.10	-0.06	-0.49	-0.14	0.92	-0.10	0.10	-0.36	0.11	-0.70	0.47	-0.10	X-ray Gibbsite Conc.
					1.00	-0.27	0.17	0.48	0.47	0.95	0.43	0.87	0.81	0.93	-0.37	-0.90	-0.88	-0.71	0.69	-0.66	0.68	0.09	0.09	0.27	-0.22	-0.66	-0.34	0.91	0.11	-0.11	-0.46	0.05	-0.78	0.64	0.03	X-ray Gibbsite Charge
						1.00	0.84	-0.06	-0.07	-0.36	-0.77	-0.55	-0.67	-0.34	0.66	0.16	0.55	0.64	-0.66	0.78	-0.72	-0.79	-0.79	-0.76	0.76	0.68	0.93	-0.09	-0.77	0.74	0.81	0.48	0.63	-0.75	-0.21	Extn Hydrous Fe Oxide Conc
							1.00	0.18	0.17	0.09	-0.48	-0.10	-0.23	0.14	0.36	-0.27	0.10	0.19	-0.28	0.36	-0.36	-0.64	-0.64	-0.50	0.54	0.20	0.70	0.31	-0.65	0.68	0.57	0.46	0.23	-0.37	-0.24	Extn Hydrous Fe Oxide Charge
								1.00	1.00	0.47	0.12	0.45	0.44	0.55	-0.33	-0.49	-0.48	-0.30	0.49	-0.32	0.48	0.00	0.00	0.18	-0.07	-0.30	-0.11	0.47	0.03	-0.16	-0.15	0.16	-0.36	0.37	0.21	X-ray Goethite Conc.
									1.00	0.46	0.12	0.45	0.44	0.54	-0.34	-0.48	-0.47	-0.30	0.50	-0.33	0.48	0.01	0.01	0.19	-0.07	-0.31	-0.12	0.46	0.05	-0.17	-0.15	0.15	-0.35	0.37	0.22	X-ray Goethite Charge
										1.00	0.53	0.93	0.86	0.94	-0.45	-0.89	-0.93	-0.76	0.72	-0.74	0.72	0.23	0.23	0.40	-0.35	-0.74	-0.44	0.89	0.18	-0.10	-0.57	-0.09	-0.87	0.71	-0.01	LogSum HF/HAO Goeth/ Gibbs Charge
											1.00	0.68	0.77	0.57	-0.70	-0.39	-0.73	-0.71	0.70	-0.81	0.72	0.82	0.82	0.89	-0.88	-0.78	-0.88	0.33	0.81	-0.38	-0.79	-0.65	-0.74	0.72	-0.03	X-ray Illite Conc
												1.00	0.83	0.83	-0.66	-0.72	-0.87	-0.86	0.65	-0.79	0.67	0.42	0.42	0.48	-0.47	-0.77	-0.61	0.77	0.24	-0.19	-0.78	-0.22	-0.94	0.69	-0.17	X-ray Kaolinite Conc
													1.00	0.87	-0.70	-0.73	-0.96	-0.85	0.88	-0.94	0.89	0.56	0.56	0.69	-0.66	-0.92	-0.78	0.70	0.57	-0.43	-0.75	-0.24	-0.90	0.90	0.13	X-ray Kaolinite Charge
														1.00	-0.45	-0.96	-0.92	-0.81	0.77	-0.74	0.75	0.21	0.21	0.45	-0.36	-0.77	-0.44	0.89	0.21	-0.10	-0.53	-0.02	-0.87	0.70	0.05	Textural Clay
															1.00	0.17	0.64	0.64	-0.64	0.76	-0.67	-0.65	-0.65	-0.57	0.62	0.73	0.80	-0.22	-0.55	0.55	0.64	0.47	0.65	-0.67	0.13	Textural Silt
																1.00	0.81	0.69	-0.63	0.57	-0.60	-0.01	-0.01	-0.31	0.19	0.60	0.22	-0.91	-0.05	-0.07	0.37	-0.13	0.75	-0.55	-0.10	Textural Sand
																	1.00	0.84	-0.80	0.88	-0.81	-0.49	-0.49	-0.63	0.60	0.86	0.67	-0.82	-0.43	0.23	0.73	0.24	0.93	-0.82	0.00	X-ray Quartz Conc.
																		1.00	-0.68	0.86	-0.71	-0.51	-0.51	-0.62	0.58	0.86	0.70	-0.63	-0.38	0.31	0.72	0.26	0.91	-0.73	0.15	Extn Hydrous Mn Oxide Conc
																			1.00	-0.89	0.99	0.47	0.47	0.64	-0.56	-0.86	-0.74	0.53	0.60	-0.63	-0.62	-0.21	-0.74	0.90	0.45	Cation Exchange Capacity
																				1.00	-0.91	-0.70	-0.70	-0.78	0.76	0.98	0.88	-0.52	-0.64	0.56	0.82	0.39	0.87	-0.92	-0.16	Base Saturation
																					1.00	0.51	0.51	0.66	-0.59	-0.86	-0.79	0.51	0.62	-0.67	-0.67	-0.25	-0.77	0.91	0.43	Lime Buffer Capacity
																						1.00	1.00	0.91	-0.97	-0.64	-0.86	-0.01	0.84	-0.43	-0.75	-0.76	-0.51	0.59	-0.06	Sample Depth
																							1.00	0.91	-0.97	-0.64	-0.86	-0.01	0.84	-0.43	-0.75	-0.76	-0.51	0.59	-0.06	Estimated Hydrostatic Pressure
																								1.00	-0.97	-0.75	-0.84	0.19	0.84	-0.38	-0.73	-0.62	-0.62	0.69	0.10	Estimated Fraction Saturation
																									1.00	0.73	0.85	-0.15	-0.85	0.37	0.75	0.70	0.58	-0.66	0.00	Measured Solids Content
																										1.00	0.80	-0.54	-0.59	0.46	0.72	0.36	0.83	-0.87	-0.12	Soil pH
																											1.00	-0.17	-0.82	0.69	0.85	0.58	0.72	-0.81	-0.12	LogMehlich Ca
																												1.00	-0.04	0.18	-0.42	0.11	-0.72	0.52	-0.09	LogMehlich Mg
																													1.00	-0.59	-0.62	-0.56	-0.38	0.63	0.29	LogMehlich Na
																														1.00	0.45	0.18	0.28	-0.59	-0.54	LogMehlich K
																															1.00	0.53	0.83	-0.77	0.06	LogMehlich Fe
																																1.00	0.32	-0.19	0.33	LogMehlich Pb
																																	1.00	-0.78	0.11	LogP
																																		1.00	0.33	LogCl
																																			1.00	LogNO3

- Positive correlation, Nominal P = 0.01
- Positive correlation, Nominal P = 0.05
- Positive correlation, Nominal P = 0.10
- Negative correlation, Nominal P = 0.10
- Negative correlation, Nominal P = 0.05
- Negative correlation, Nominal P = 0.01

818

IV. Extended PFAS Summary

PFAS are an extensive family of anthropogenic compounds found in both industrial and consumer products with high resistance to thermal, chemical and microbial degradation and have potentially deleterious effects on exposed organisms [51]. PFAS can enter groundwater from numerous sources [52] and a significant source stems from solid waste generated during waste treatment processes. It's estimated that more than 10 million tons of sludge are generated annual in the U.S. and European Union [53], and once stabilized and treated, the sludge, now biosolids, can contain PFAS in the µg/g range [54]. With more than half of biosolids in the U.S. applied to public and private land, this potentially serves as a source of these pollutants directly to the terrestrial environment and subsequently groundwater [55]. Of particular concern are industrial waste-water treatment facilities, with total target PFAS concentrations in the effluents in excess of 4,200 ng/L[56], relative to municipal wastewaters of up to 71.5 ng/L [57]. Commercial users such as textile mills are large consumers of PFAS products and the often-overlooked textile production and waste stream is believed to be among the higher sources of PFAS in the environment, accounting for close to 30% of annual PFOS discharge in China [58]. Consequently, both active and former textile mills can represent significant sources of ongoing PFAS release to the environment [59].

Challenges associated with PFAS modeling to groundwater are exacerbated due to the diversity of the chemical group. With >1400 individual chemicals and >8000 unique structures [51], there is a need to identify general behavioral trends within the class. The continuous development of next-generation and ever-expanding list of industrial and consumer uses, has resulted in an ongoing challengingly evolving need for measurement and remediation. PFAS commonly contain aliphatic chains of variable length and hydrophilic functional head groups, such as carboxylic or sulfonic acids [60] suggesting the critical role of hydrophobic and/or electrostatic interactions in PFAS retention and mobility [61]. They are described as long- or short-chain, where long-chain PFAS typically have 7 or more fluorinated carbons for PFCAs or 6 or more fluorinated carbons for PFSAs, and exhibit fundamental differences in mobility and potential bioaccumulation around this fluorinated carbon length [62]. In soils, the distribution of PFAS with depth is highly dependent on chain-length, with long-chain PFAS commonly occupying the first 1 m below ground surface (bgs) while short-chain PFAS, which typically have higher solubility, mobility and bioavailability, migrate to further depths, and have higher occurrences in the groundwater [63-65]. The more common short-chain PFAS detections in groundwater are believed to be associated with a lower octanol-water partitioning coefficient and higher water solubility relative to their long-chain counterparts [66].

In the present study, twelve of the twenty-five targeted analytes were detected in the colluvial Field 1 shallow groundwater and ten were detected in the residual Field 2 shallow groundwater, albeit at much lower concentrations (Supplementary Tables 16-17). Concentrations of PFBA-PFOA were 3-4 times the concentration in Field 1 shallow groundwater relative to the Field 2 shallow groundwater, which is consistent with the total biosolid loading difference of 3-4 times the applied tons/acre in Field 1 relative to Field 2 (Supplementary Fig. 1). Among the PFCAs in groundwater, PFOA is the most concentrated, with detection at 6,300 parts per trillion (ppt, or ng/L), followed by PFHxA at 3,900 ppt. PFBS was the highest detected PFSA, at 12,000 ppt, followed by PFOS at 3,400 ppt. The USEPA previously issued 2016 Drinking Water Health Advisories for PFOA and PFOS sum to ≤70 ng/L and updated them with 2022 Drinking Water Interim Health Advisories including PFOA ≤ 0.004 ng/L [67,68]. The sum of PFOA and PFOS in shallow groundwater of Field 1 was 9,700 ng/L, more than 130-times the 2016 health advisory value and 2.4-million-times the 2022 interim value (Figure 3G and Supplementary Table 16). The sum of PFOA and PFOS in shallow groundwater of Field 2 was 2,200 ppt, while consisting of only PFOA, is still more than 30-times the health advisory value (Figure 3G and Supplementary Table 17). Further in the deep groundwater well, eight of the twenty-five targeted analytes were detected in Field 1. The highest detected analytes included PFHxA, PFOA, and PFBS (Supplementary Table 16). No PFAS were detected in the Field 2 deep groundwater.

Four surface-soil grab samples were collected and homogenized from hand-augured borings from 0 to 0.15 m (0 to 0.5 ft) bgs and labeled SF101-104 in Field 1 and SF201-204 in Field 2 and compared to the first surface-soil boring sample from each site (SB01 and SB02, for Field 1 and Field 2) (Supplementary Tables 14-15). Of the twenty-five targeted PFAS, twenty-two analytes were detected in at least one surface-soil sample. Overall, the PFAS concentrations in Field 1 were around 2.5-4.5 times the concentrations in Field 2, consistent with the higher PFAS loading rate in Field 1 relative to Field 2 (Supplementary Fig. 1).

Biosolids application was reported to be uniformly applied to fields, however there are slight hotspots of higher and lower surface PFAS concentrations in several surface soils, such as SF103 in Field 1 relative to other Field 1 surface soils (Supplementary Fig. 13). This is more evident when comparing the Field 1 average 2021 surface samples (F1 ’21) relative to a 2018 sampling conducted in the same field (F1 ‘18) [69] (Supplementary Fig. 13). Although tempting to speculate that the higher detections of PFAS in Field 1 in 2021 relative to 2018 is solely due to PFAS precursors transformation and subsequent increase in the Field 1 PFCAs and PFSAs in the three years between sampling, the lower detections of the targeted PFAS precursors, FTS, N-MeFOSAA, and FOSA, in 2018 in addition to the lower PFCAs and PFSAs suggests that this increase in 2021 is likely a consequence of nonuniform biosolid distribution

horizontally across the fields. The surface soil grab sample in Field 1 in 2018 has comparable PFAS concentrations to the Field 1 2021 sample SF103.

In both fields, the surface soils were dominated by long-chain PFAS, particularly the PFCAs, with highest detections for PFDoA (detected at 78,000 ng/kg), followed by PFDA (73,000 ng/kg), PFUdA (69,000 ng/kg), PFTrDA (45,000 ng/kg), PFTeDA (20,000 ng/kg) and PFOA (9,500 ng/kg). The highest PFSA was PFOS (42,000 ng/kg), followed by PFBS (5,500 ng/kg). This is in sharp contrast to the groundwater detections, which were primarily short-chain PFAS, with no groundwater detections for PFCAs longer than PFDA. Plotting the concentration in the groundwater relative to the concentration in the surface soil as a function of fluorinated carbon count gives an indication of mobility of PFCAs and PFSAs through soils to groundwater relative to chain-length (Figure 3F).

Continuing with depth, vertical migration of each targeted PFAS analyte in each soil profile was evaluated by analyzing the soil concentration at each depth interval (Supplementary Tables 14-15 and Supplementary Figs. 14-15). Twenty-three of twenty-five analytes were detected in at least a single soil extract. The highest of all detections in the profiles were for long-chains PFAS PFDA > PFOS > PFDoA ≥ PFUdA, with concentrations at 82,000, 81,000, 78,000, and 69,000 ng/kg and 31,000, 27,000, 17,000, and 17,000 ng/kg in Field 1 and Field 2, respectively, with these maximum concentrations occurring at or above 0.46 m (1.5 ft). In Field 1, the maximum detections for shorter-chain PFAS (PFBA-PFOA, PFBS-PFHpS) were detected in the subsurface from 1.5 to 2.4 m (5 to 8 ft), with the highest detection for PFBS (15,000 ng/kg at a mid-depth of 2.3 m or 7.4 ft). The shorter-chains migrated further through the subsoils than the long-chains, with detections below 12 m, reflecting an overall higher mobility of the shorter-chain PFAS through soils to groundwater relative to the longer-chain PFAS (Supplementary Figs. 14-15). Radar plots of the PFCA concentrations, normalized to highest detection in soil per field per analyte, with depth bgs, in meters, reveals similarities between behavior of PFAS analyte species (Supplementary Fig. 16). In both fields, PFBA-PFOA behave similarly through depth with the bulk of detections from 0.2 to 3 or 4 m, and PFDA-PFTeDA behave similarly through depth, with the bulk of detections at the surface above 0.4 m. PFNA, while not overlaying entirely with the shorter PFCAs, exhibits a migration profile more similarly to the short-chains than the long-chains. It is interesting to note that while short-chain PFCAs are defined as having less than eight carbons [70], PFOA, and to some extent PFNA, behave more similarly to the short-chain PFAS in the subsurface than their long-chain counterparts. This is supported by nominal correlations shown in the heatmap in Supplementary Figs. 17-18, where PFBA-PFOA species are highly correlated and PFDA-PFTeDA are highly correlated. Again, PFNA does correlate with most of the shorter-chain PFCAs or with PFDA, suggesting similarities in subsurface behavior amongst these species. Similarly, while short-chain PFSAs are defined as having less than six carbons [70], PFHxS highly correlates with the other short-chain PFSAs PFBS and PFPeS, and these three analytes as well as seven

fluorocarbon species PFHpS highly correlate with the shorter-chain PFCAs. PFOS highly correlates with PFHpS and PFOA, PFNA, and PFDA. Precursors 8:2FTS, N-EtFOSAA, and FOSA, which were detected primarily at the surface, correlated highly with the long-chain PFCAs PFUdA-PFTeDA, which were also predominately retained at the surface (Supplementary Figs. 17-18).

Field 1 received close to 3.7 times the quantity of biosolids per acre compared to Field 2 (Supplementary Fig. 1), and, summing PFAS through all depths for each soil boring sample (SB01 and SB02 for Field 1 and Field 2) reveals a sum of PFAS that is consistent with the total biosolid applications for most analytes (Supplementary Fig. 19). Notable exceptions, however, include PFOA and PFBS. While surface concentrations of PFOA between fields were detected in ratios similar to the biosolids-loading rate ratio, surface-soil concentration of PFOA in Field 1 is 2.6-fold higher than in Field 2, the summed concentration of PFOA through depth is equal in both fields (Supplementary Tables 14-15). And unexpectedly, the highest PFOA detection can be found in Field 2, rather than Field 1, at a concentration of 16,000 ng/kg dry soil at a sample mid-depth of 0.99 m (3.25 ft). Regarding PFBS, it is among the highest detected short-chain PFAS in Field 1, but it is almost non-detect in Field 2 (Supplementary Tables 14-15). Whether these deviations from anticipated concentrations are from degradation of unmeasured precursors altering ratios of terminal PFAS, variation in mobility of these specific PFAS species over time, or temporally unique PFAS products in the applied biosolids is unclear from these data. Short-chain PFCAs and PFSAs are generally more environmentally mobile in an aqueous environment, and considering that PFBS was not detected at the surface of Field 2 and was only detected in low concentrations in the shallow groundwater at 4.6 m (15 ft) and beyond the water table in the deep subsurface from 5.5 to 8.5 m (18 to 28 ft), it is unclear if this observation is due solely to temporal differences in application. While, it is possible PFBS has migrated from the surface through the subsurface in the last 20 years since application in Field 2, relative to Field 1 which received a large quantity of biosolids nearly 10 years after the bulk of the Field 2 application, column studies reveal similarities between PFBS and PFBA in retention factors and partitioning coefficients [71], suggesting some similarity in the subsurface mobility of these analytes. Thus differences in biosolids PFBS precursors between the fields cannot be ruled out.

Under environmental conditions, in addition to abiotic degradation, microbe-facilitated biotransformation occur aerobically [72,73] and anaerobically [74-76]. To gauge the possibility of *in situ* transformation of polymer and precursor PFAS to intermediate or terminal and recalcitrant PFAS in the agricultural fields, relative ratios of the PFAS precursors observed in the surface soils were investigated. Of the six targeted PFAS precursors, four were detected in Field 1 and three were detected in Field 2 (Supplementary Tables 14-15). Curiously, surface concentrations of 8:2FTS, N-EtFOSAA, N-MeFOSAA,

and FOSA were all comparable within Field 1, yet only 8:2FTS, N-MeFOSA, and FOSA were detected in Field 2. In the absence of any transformation and discounting the possibly of vertical leaching of these precursors to the subsurface, one would expect that the concentration of N-EtFOSAA in Field 2 would be detected similar in magnitude to precursors N-MeFOSAA and FOSA and close to that of 8:2FTS in Field 2. Reflecting on the temporal differences in biosolid loadings previously discussed, the fact that the bulk of all biosolids were applied prior to 2002 in Field 2, relative to about half of all biosolids applied prior to 2002 in Field 1 and another substantial application loaded up until 2012 could suggest complete degradation of N-EtFOSAA in the more aged Field 2 with a reintroduction of more precursor in Field 1. Note, that N-EtFOSE is believed to degrade to N-EtFOSAA, which then is converted to EtFOSA [51]. While the transformation of precursors N-EtFOSA and N-EtFOSE are relatively fast, with reported half-lives of <1 day in activated sludge and on the order of several weeks in aerobic soils [73], the transformation of N-EtFOSAA is believed to be longer, with a half-life of > 1 week in activated sludge [77] and expected to be much longer in soil environments. These detections prompted the nontargeted analysis (NTA) of select samples, including surface soils from Fields 1 and 2, and a few compounds were tentatively identified including:

1) 2-[3-(Difluoromethyl)-5-methyl-1H-pyrazol-1-yl]-N'-[(1Z)-3-methylcyclohexylidene]acetohydrazide ($C_{14}H_2OF_2N_4O$);
2) N-(3-Amino-2,2-difluoropropyl)-2-(4-benzyl-1-piperazinyl)acetamide ($C_{16}H_{24}F_2N_4O$);
3) 1,1,1,2,2,3,3,4,4,5,5-Undecafluoro-7-iodododecane ($C_{12}H_{14}F_{11}I$);
4) Hydro-polyfluorodecanesulfonate ($C_{10}H_2F_{20}O_3S$);
5) Hydro-polyfluorododecanesulfonate ($C_{12}H_2F_{24}O_3S$);
6) Hydro-polyfluorooctanecarboxylate ($HC_9F_{18}COOH$);
7) Hydro-polyfluorodecanecarboxylate ($HC_{11}F_{22}COOH$);
8) N:2FTCA ($F(CF_2)_NCH_2COOH$; N=6,8,10)
9) N:2FTUCA ($F(CF_2)_{(N-1)}CFCHCOOH$; N=6,8,10)
10) (N-1):3FTUCA ($F(CF_2)_{(N-1)}(CH)_2COOH$; N=6,8,10)
11) (N-1):3FTCA ($F(CF_2)_{(N-1)}(CH_2)_2COOH$; N=6,8,10)
12) $C_{14}H_{16}F_{13}I$

Compounds 1-7 have been previously reported by this laboratory, detected in commercial fluorotelomer-based dispersions and surfactants [78]. Compounds 8-11 have been reported in the literature previously as intermediate products of sidechain polymer degradation [79]. An additional iodine containing PFAS is tentatively reported, as a potential congener of 1,1,1,2,2,3,3,4,4,5,5-Undecafluoro-7-iodododecane. Altogether, the occurrence of precursor PFAS including relatively short-lived FTOHs more than one

decade (Field 1) and more than two decades (Field 2) after application and other intermediate PFAS more than a decade after application suggests the possibility of degradation of sidechain polymers as posited in an earlier study of biosolids-applied soils [80].

Primary mechanisms thought to be responsible for PFAS partitioning to sorbents are hydrophobic effects and electrostatic interactions [81-83]. For hydrophobic effects, the hydrophobicity of the solid sorbents and the PFAS carbon chain length play a role, while electrostatic interactions can be influenced by factors including the sorbent surface charge at a specific pH or the PFAS functional head group [81]. Sorption onto carbon-based sorbents increases with increasing CF_2 count, likely due to increased hydrophobic interactions [84], and as expected, the long-chain PFAS are highly correlated with the total organic carbon (TOC) in all correlation analyses including data above the water table (Supplementary Fig. 20 and Supplementary Figs. 24-26). Similarly, the long-chain PFAS also displayed high positive correlations with HFO concentrations, total nitrogen, and Mn oxides. Of note are previously reported associations of organic matter (OM) with ferric oxide mineral(oid)s, particularly in sandy soils where organo-minerals associations include more aliphatic OM structures, and strong correlations between mineral-bound organic carbon and iron oxides have been shown in subsoils [85], and Mn and Fe oxides, which can result from migration of Mn(II) and Fe(II) migration and local reoxidation and co-precipitation [86]. This suggests at least in part co-correlation between these variables in the organic rich surface soil. Interestingly, the long-chain PFAS, although highly correlated with the HFO concentrations, had no correlations to the calculated surface charges, further supporting the co-association with these sorbents in the surface soil and possible hydrophobic interactions, rather than any electrostatic attractions.

In addition to the solid-phase sorption, the contribution of air-water interface adsorption has been shown to play a critical role in retention of PFAS in the soil environment [87]. As described in earlier sections, Brusseau has shown that air-water interfacial adsorption was greater than the solid-phase sorption for long-chain PFAS, but less than that for shorter-chain PFAS [87]. The high correlations with the air-water interface and the long-chain PFAS, but not the short-chain PFAS, are consistent with these reported findings in all unsaturated media using two Brusseau [35] methods to calculate the interfacial adsorption, Equations 4 and 5 of Brusseau, herein Supplementary Equation 19 and 20 (Supplementary Figs. 24-26).

Models of PFAS sorption in soils and sediments highlight the importance of soil OM and clay fractions, with electrostatic attractions playing a role in the absence of organic matter [88,89] and some previous studies have focused on clays [89-93], and metal oxides [89,92,94,95]. The short-chain PFAS, including PFOA, were highly correlated with the HAO and gibbsite, as well as the calculated surface charges, supporting the importance of electrostatics in PFAS subsurface mobility. Previous studies have found that

aluminum oxides may inhibit migration of PFOA relative to iron oxides in porous media [96], and this is consistent with the findings in the current study. Additionally, short-chain PFAS including PFOA were also highly positively correlated with clay fractions, including textural clay and kaolinite clay fractions and the surface charge associated with kaolinite. Field 1, which contained high levels of PFSAs relative to Field 2, was also analyzed individually, Supplementary Fig. 25, and the short-chain sulfonates were found to behave similarly to the carboxylates. Short-chain PFBS-PFHxS/PFHpS is highly correlated with the HAO concentrations and surface charges and the clay fractions like kaolinite in the unsaturated media, above the water table (Figure 5). Interestingly, simulations found significant variation in molecular clustering and coordination between the PFAS and the hydroxyl surfaces of kaolinite among molecules of different chain-length and functional group, suggesting short-chain sulfonate PFAS molecules experience more restrictive transport in environmental soils with a higher kaolinite compared to carboxylates [97]. These studies could provide some mechanistic insight for the significantly higher correlations for short-chain sulfonates with kaolinite in Field 1 unsaturated media relative to short-chain carboxylates (Supplementary Fig. 25). PFAS data below the water table (saturated media) had too few detections for reliable correlation calculations.

For all correlation tables and heatmaps, significant negative correlations were also observed, particularly for variables such as sample depth and hydrostatic pressure. In many of these instances, it is likely that significant inverse correlations are an artifactual consequence of the PFAS deposited at the land surface, as PFAS applied at the surface will generally decrease with increasing depth due to retardation, as well as the distribution of authigenic phases, a consequence of the pedogenic processes. For example, the long-chain PFAS are immobilized in the surface, <0.5 m depth interval, while authigenic clays increase with depth toward maximum values at ≥1.0 m. Long-chain PFAS are immobilized by other soil phases prior to encountering the highest clay concentrations; this is similar to the inverse correlations with the long-chain PFCAs and aluminum minerals and goethite. Nevertheless, correlations with PFAS and soil geochemical data show statistically significant relationships of individual species of PFAS with geochemical parameters, suggesting mechanistic control of these geochemical phases on the mobility of these PFAS species.

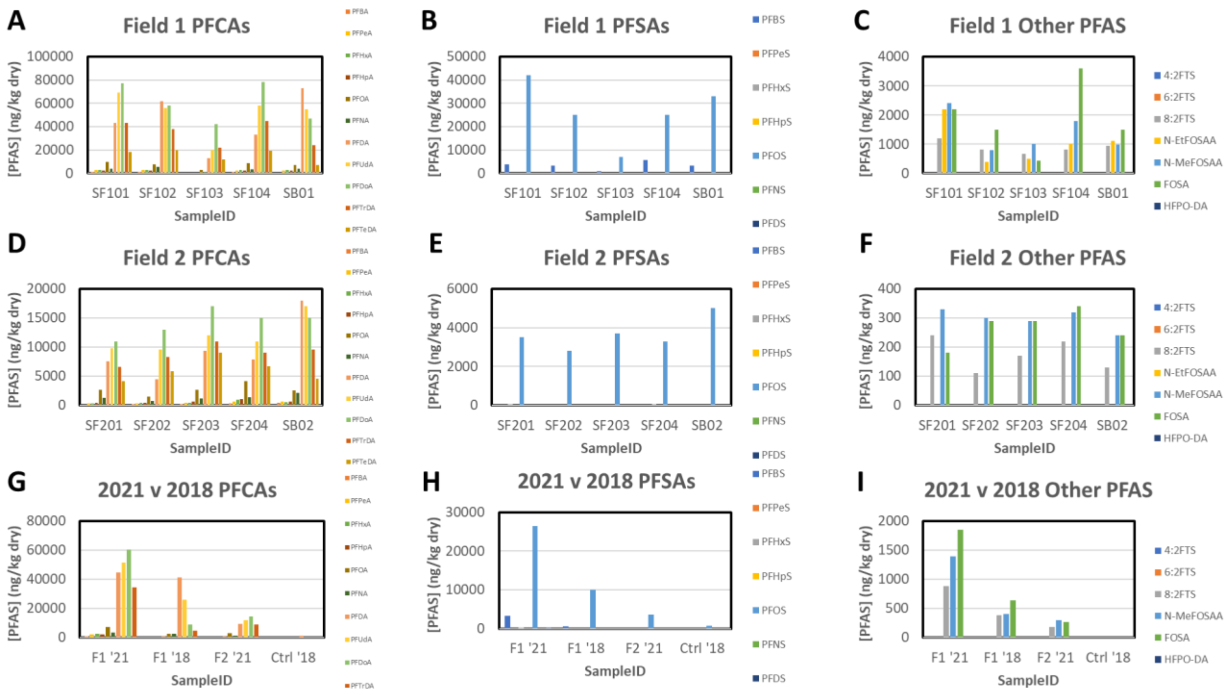

Supplementary Fig. 13. **Concentration of PFAS (ng/kg dry soil) for all hand augured (SF) and soil boring (SB) surface soils collected in 2021, from 0-0.15 m (0-0.5 ft) bgs (a-f) and relative to surface soils collected in 2018 (g-i).** 2018 data has been previously reported [69]. Field 1 PFCAs (**a**), PFSAs (**b**), and other PFAS (**c**) are on average, >2.5x the concentrations of Field 2 PFCAs (**d**), PFSAs (**e**), and other PFAS (**f**). Average Field 1 concentrations of all PFAS (**g-i**) in 2021 are elevated relative to the single sample collected in the same field in 2018. Note that a sample in Field 2 was not collected in 2018. The control 2018 (Ctrl '18) sample in (**g-i**) was collected from an adjacent field, which did not receive sludge applications from Galey & Lord and has been previously reported [69]. Additionally, analytes PFTeDA and N-EtFOSAA were not analyzed in 2018 and are not included in plots (**g-i**).

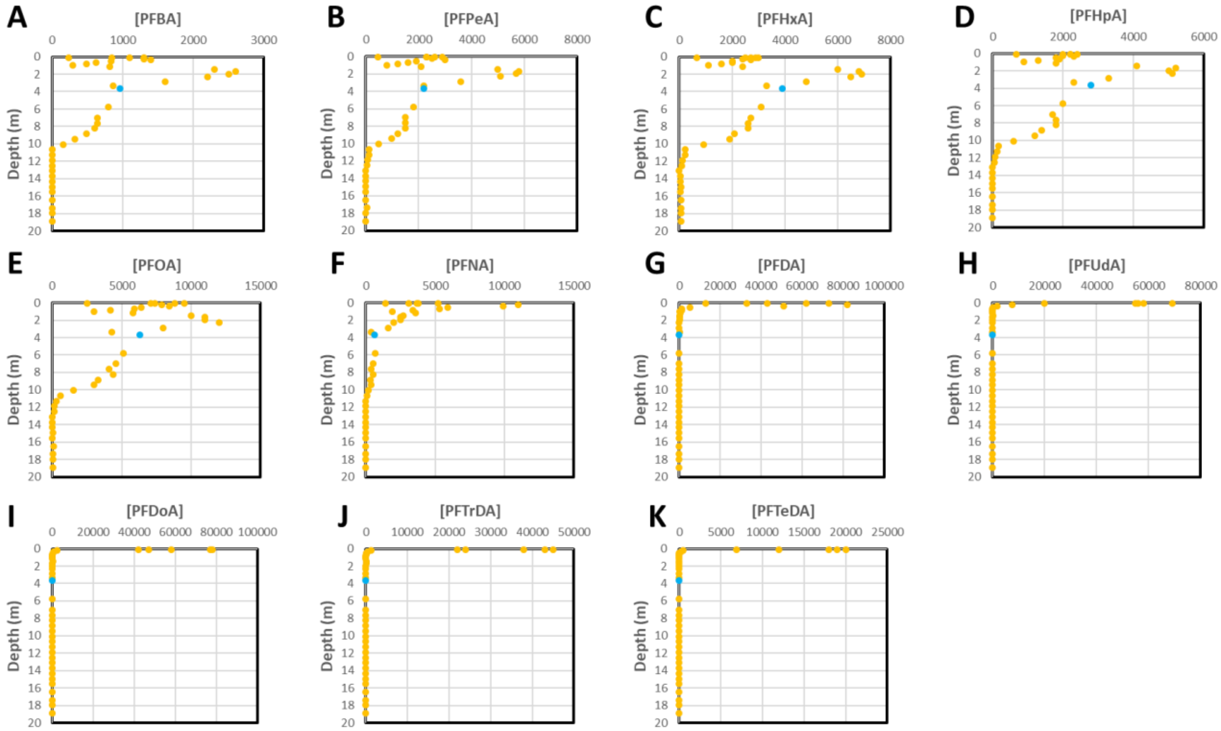

Supplementary Fig. 14. **Depth profile for PFCA concentrations in Field 1 with depth from surface to ~20 m for each analyte in ng/kg dry soil (orange) and the shallow groundwater in ng/L (blue).** PFDA and longer-chain PFCAs were non-detect in groundwater. Field 1 PFCAs include PFBA (**a**), PFPeA (**b**), PFHxA (**c**), PFHpA (**d**), PFOA (**e**), PFNA (**f**), PFDA (**g**), PFUdA (**h**), PFDoA (**i**), PFTrDA (**j**), and PFTeDA (**j**).

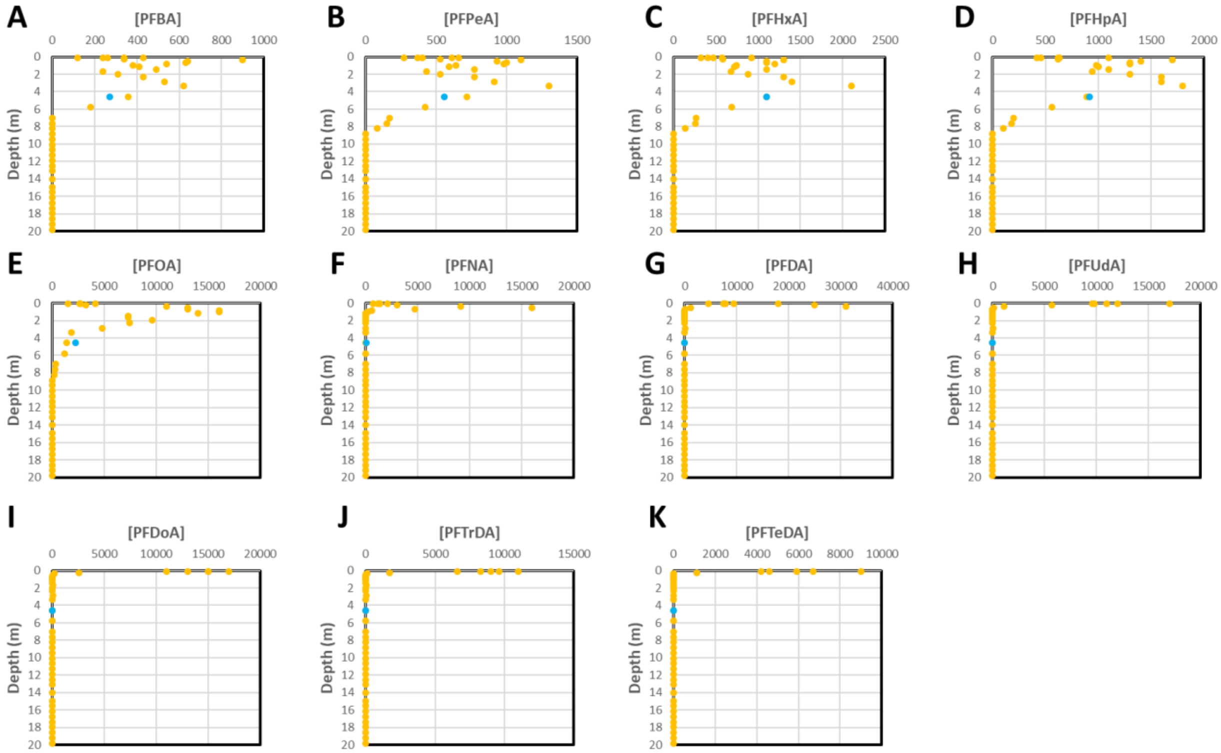

Supplementary Fig. 15. **Depth profile for PFCA concentrations in Field 2 with depth from surface to ~20 m for each analyte in ng/kg dry soil (orange) and the shallow groundwater in ng/L (blue).** PFNA and longer-chain PFCAs were non-detect in groundwater. Field 2 PFCAs include PFBA (**a**), PFPeA (**b**), PFHxA (**c**), PFHpA (**d**), PFOA (**e**), PFNA (**f**), PFDA (**g**), PFUdA (**h**), PFDoA (**i**), PFTrDA (**j**), and PFTeDA (**j**).

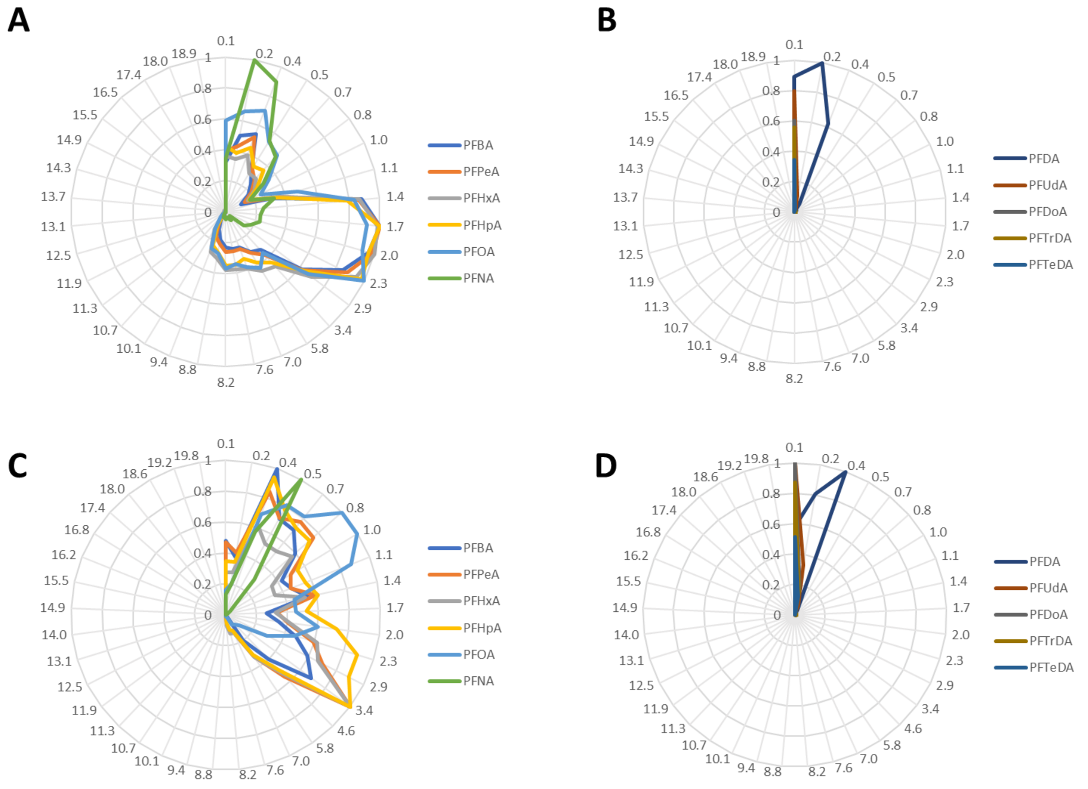

Supplementary Fig. 16. **Radar plots, normalized to maximum PFCA analyte concentrations (inside from 0 to 1) with depth (outer from 0.1 to ~ 20, in meter) of Field 1 PFBA-PFNA (a) and PFDA-PFTeDA (b) and Field 2 PFBA-PFNA (c) and PFDA-PFTrDA (d).**

		PFCAs											PFSAs							Other PFAS						
		PFBA	PFPeA	PFHxA	PFHpA	PFOA	PFNA	PFDA	PFUdA	PFDoA	PFTrDA	PFTeDA	PFBS	PFPeS	PFHxS	PFHpS	PFOS	PFNS	PFDS	4:2FTS	6:2FTS	8:2FTS	N-EtFOSAA	N-MeFOSAA	FOSA	HFPO-DA
PFCAs	PFBA	1.00	0.99	0.92	0.96	0.96	0.53	-0.09	-0.28	-0.19	-0.33	-0.30	0.75	0.68	0.72	0.87	0.41	-0.29	0.50			-0.06	-0.07	0.35	0.02	
	PFPeA	0.99	1.00	0.95	0.98	0.94	0.48	-0.17	-0.28	-0.24	-0.37	-0.26	0.80	0.74	0.75	0.83	0.35	-0.28	0.39			0.03	0.01	0.25	0.06	
	PFHxA	0.92	0.95	1.00	0.97	0.83	0.19	-0.33	-0.28	-0.24	-0.39	-0.13	0.94	0.81	0.88	0.64	0.05	0.15	0.46			0.11	0.10	0.30	0.21	
	PFHpA	0.96	0.98	0.97	1.00	0.91	0.36	-0.28	-0.32	-0.32	-0.46	-0.20	0.86	0.81	0.82	0.76	0.23	-0.03	0.42			0.08	0.07	0.26	0.16	
	PFOA	0.96	0.94	0.83	0.91	1.00	0.67	0.10	-0.13	-0.12	-0.24	-0.19	0.63	0.51	0.61	0.95	0.58	0.55	0.61			0.07	0.11	0.49	0.17	
	PFNA	0.53	0.48	0.19	0.36	0.67	1.00	0.59	-0.18	-0.36	-0.21	-0.77	-0.09	0.26	-0.14	0.72	0.97	-0.76	0.15			-0.59	-0.61	0.01	-0.52	
	PFDA	-0.09	-0.17	-0.33	-0.28	0.10	0.59	1.00	0.77	0.72	0.76	-0.39	-0.52	-0.94	-0.33	0.13	0.77	-0.54	0.01			-0.26	-0.30	-0.09	-0.05	
	PFUdA	-0.28	-0.28	-0.28	-0.32	-0.13	-0.18	0.77	1.00	0.97	0.97	0.93	-0.46	-0.92	-0.18	-0.27	0.11	0.70	0.55			0.94	0.90	0.43	0.98	
	PFDoA	-0.19	-0.24	-0.24	-0.32	-0.12	-0.36	0.72	0.97	1.00	1.00	0.99	-0.39	-0.91	-0.10	-0.26	-0.06	0.67	0.90			0.97	0.92	0.79	0.93	
	PFTrDA	-0.33	-0.37	-0.39	-0.46	-0.24	-0.21	0.76	0.97	1.00	1.00	0.99	-0.51	-0.94	-0.28	-0.38	0.13	0.64	0.77			0.96	0.90	0.62	0.93	
	PFTeDA	-0.30	-0.26	-0.13	-0.20	-0.19	-0.77	-0.39	0.93	0.99	0.99	1.00	-0.17	1.00	0.04	-0.45	-0.70	0.57	0.50			0.92	0.84	0.37	0.89	
PFSAs	PFBS	0.75	0.80	0.94	0.86	0.63	-0.09	-0.52	-0.46	-0.39	-0.51	-0.17	1.00	0.83	0.92	0.44	-0.23	0.24	0.62			0.09	0.14	0.46	0.19	
	PFPeS	0.68	0.74	0.81	0.81	0.51	0.26	-0.94	-0.92	-0.91	-0.94	1.00	0.83	1.00	0.96	0.48	0.04	-1.00	-1.00			-1.00	-1.00	-1.00	1.00	
	PFHxS	0.72	0.75	0.88	0.82	0.61	-0.14	-0.33	-0.18	-0.10	-0.28	0.04	0.92	0.96	1.00	0.47	-0.21	0.72	0.84			0.28	0.41	0.75	0.38	
	PFHpS	0.87	0.83	0.64	0.76	0.95	0.72	0.13	-0.27	-0.26	-0.35	-0.45	0.44	0.48	0.47	1.00	0.64	0.26	0.84			-0.16	-0.04	0.77	-0.10	
	PFOS	0.41	0.35	0.05	0.23	0.58	0.97	0.77	0.11	-0.06	0.13	-0.70	-0.23	0.04	-0.21	0.64	1.00	-0.27	0.51			-0.40	-0.33	0.44	-0.38	
	PFNS	-0.29	-0.28	0.15	-0.03	0.55	-0.76	-0.54	0.70	0.67	0.64	0.57	0.24		0.72	0.26	-0.27	1.00	0.65			0.78	0.90	0.79	0.67	
	PFDS	0.50	0.39	0.46	0.42	0.61	0.15	0.01	0.55	0.90	0.77	0.50	0.62		0.84	0.84	0.51	0.65	1.00			0.58	0.77	0.97	0.73	
Other PFAS	4:2FTS																									
	6:2FTS																									
	8:2FTS	-0.06	0.03	0.11	0.08	0.07	-0.59	-0.26	0.94	0.97	0.96	0.92	0.09		0.28	-0.16	-0.40	0.78	0.58			1.00	0.96	0.62	0.81	
	N-EtFOSAA	-0.07	0.01	0.10	0.07	0.11	-0.61	-0.30	0.90	0.92	0.90	0.84	0.14		0.41	-0.04	-0.33	0.90	0.77			0.96	1.00	0.85	0.82	
	N-MeFOSAA	0.35	0.25	0.30	0.26	0.49	0.01	-0.09	0.43	0.79	0.62	0.37	0.46		0.75	0.77	0.44	0.79	0.97			0.62	0.85	1.00	0.58	
	FOSA	0.02	0.06	0.21	0.16	0.17	-0.52	-0.05	0.98	0.93	0.93	0.89	0.19		0.38	-0.10	-0.38	0.67	0.73			0.81	0.82	0.58	1.00	
	HFPO-DA																									

Supplementary Fig. 17. **Heat map of Field 1 PFAS.** Correlations were determined for all PFAS from surface soil through subsoils, to 10.36 m or 34 ft. All red shaded or blue shaded cells are significant for positive or negative correlations, and white shaded cells are nonsignificant. Gray shaded cells with no values indicate no correlation due to too few detections.

		PFCAs											PFSAs							Other PFAS						
		PFBA	PFPeA	PFHxA	PFHpA	PFOA	PFNA	PFDA	PFUdA	PFDoA	PFTrDA	PFTeDA	PFBS	PFPeS	PFHxS	PFHpS	PFOS	PFNS	PFDS	4:2FTS	6:2FTS	8:2FTS	N-EtFOSAA	N-MeFOSAA	FOSA	HFPO-DA
PFCAs	PFBA	1.00	0.94	0.80	0.80	0.51	0.51	-0.29	-0.62	-0.56	-0.47	-0.14			0.53		0.14					-0.47		-0.71	0.66	
	PFPeA	0.94	1.00	0.96	0.96	0.79	0.44	-0.42	-0.70	-0.61	-0.53	-0.14			0.66		0.01					-0.16		-0.49	0.65	
	PFHxA	0.80	0.96	1.00	0.96	0.71	0.40	-0.49	-0.77	-0.73	-0.69	-0.03			0.82		-0.02					0.12		-0.15	0.73	
	PFHpA	0.80	0.96	0.96	1.00	0.85	0.42	-0.47	-0.77	-0.77	-0.74	0.12			0.74		0.00					0.42		0.03	0.61	
	PFOA	0.51	0.79	0.71	0.85	1.00	0.15	-0.41	-0.72	-0.84	-0.81	-0.19			-0.18		-0.34					0.77		0.18	0.15	
	PFNA	0.51	0.44	0.40	0.42	0.15	1.00	-0.25	-0.90	-0.96	-0.91	-0.74			0.09		0.84					0.29		-0.53	-0.24	
	PFDA	-0.29	-0.42	-0.49	-0.47	-0.41	-0.25	1.00	0.76	0.55	0.46	-0.70			-0.50		0.48					0.02		-0.75	-0.19	
	PFUdA	-0.62	-0.70	-0.77	-0.77	-0.72	-0.90	0.76	1.00	0.96	0.93	0.73			-0.70		-0.73					-0.30		-0.92	0.00	
	PFDoA	-0.56	-0.61	-0.73	-0.77	-0.84	-0.96	0.55	0.96	1.00	1.00	0.97			-0.39		-0.89					-0.23		-0.48	0.71	
	PFTrDA	-0.47	-0.53	-0.69	-0.74	-0.81	-0.91	0.46	0.93	1.00	1.00	0.97			-0.39		-0.96					-0.36		-0.56	0.67	
	PFTeDA	-0.14	-0.14	-0.03	0.12	-0.19	-0.74	-0.70	0.73	0.97	0.97	1.00			1.00		-0.88					-0.05		0.11	0.76	
PFSAs	PFBS																									
	PFPeS																									
	PFHxS	0.53	0.66	0.82	0.74	-0.18	0.09	-0.50	-0.70	-0.39	-0.39	1.00			1.00		-0.03					-1.00		-1.00	1.00	
	PFHpS																									
	PFOS	0.14	0.01	-0.02	0.00	-0.34	0.84	0.48	-0.73	-0.89	-0.96	-0.88			-0.03		1.00					-0.04		-0.78	-0.31	
	PFNS																									
	PFDS																									
Other PFAS	4:2FTS																									
	6:2FTS																									
	8:2FTS	-0.47	-0.16	0.12	0.42	0.77	0.29	0.02	-0.30	-0.23	-0.36	-0.05			-1.00		-0.04					1.00		0.65	-0.26	
	N-EtFOSAA																									
	N-MeFOSAA	-0.71	-0.49	-0.15	0.03	0.18	-0.53	-0.75	-0.92	-0.48	-0.56	0.11			-1.00		-0.78					0.65		1.00	-0.02	
	FOSA	0.66	0.65	0.73	0.61	0.15	-0.24	-0.19	0.00	0.71	0.67	0.76			1.00		-0.31					-0.26		-0.02	1.00	
	HFPO-DA																									

Supplementary Fig. 18. **Heat map of Field 2 PFAS.** Correlations were determined for all PFAS from surface soil through subsoils, to 8.53 m or 28 ft. All red shaded or blue shaded cells are significant for positive or negative correlations, and white shaded cells are nonsignificant. Gray shaded cells with no values indicate no correlation due to too few detections.

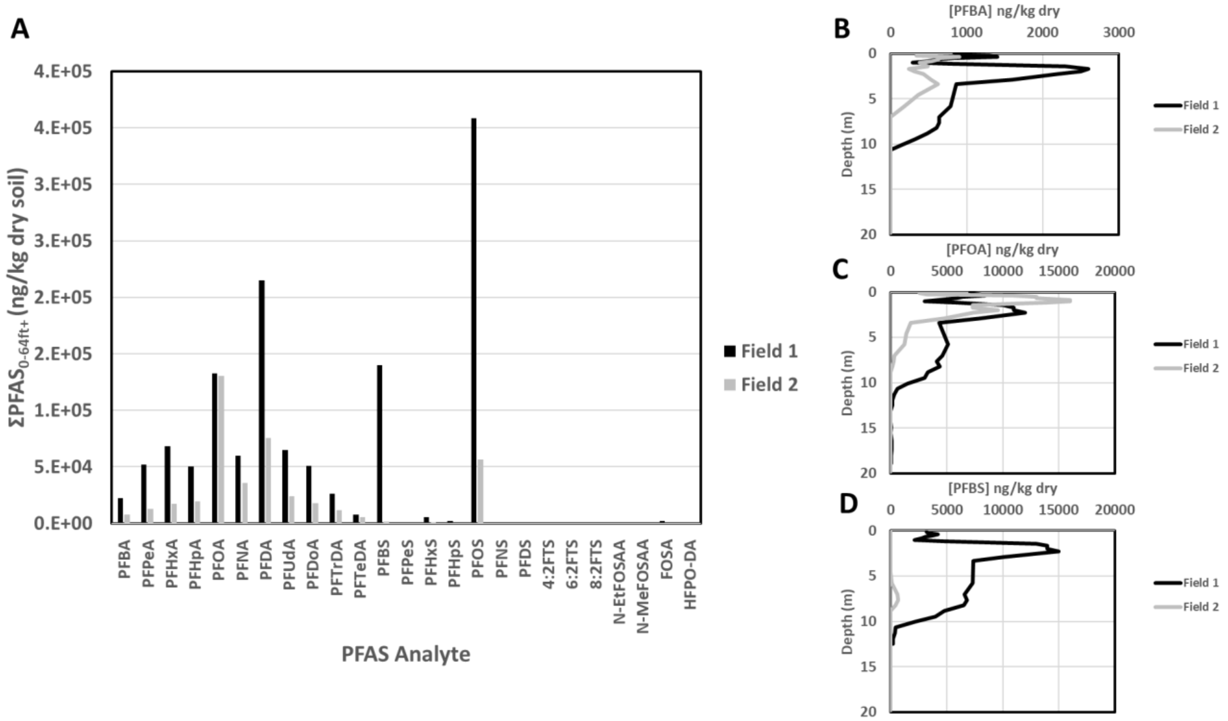

Supplementary Fig. 19. **Sum of all PFAS with depth for soil borings.** The Field 1 sum is roughly 3-fold higher than Field 2, as expected based on total biosolids loading per acre, with notable exceptions: PFOA and PFBS. Comparison of PFBA which can be found in expected ratios relative to biosolids loadings between fields (**b**), the concentration of PFOA is roughly equivalent between fields (**c**) and the concentration of PFBS is significantly lower than expected in Field 2 based on Field 1 concentrations (**d**).

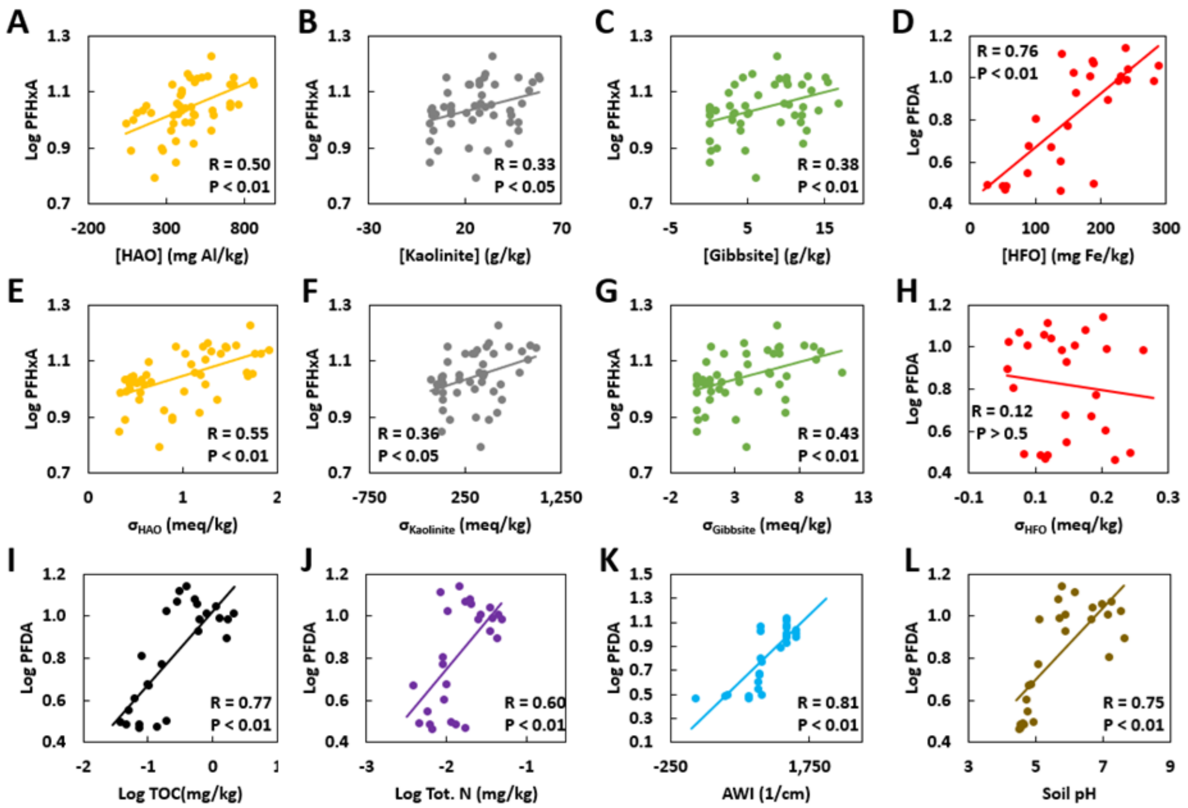

Supplementary Fig. 20. **Short-chain PFAS mobility in subsurface is controlled by electrostatic sorption, dominantly on pH-dependent charged surfaces of Al minerals**. Long-chain PFAS mobility appears to be controlled by a combination of organic matter and air-water interface. The specific minerals controlling PFAS mobility are few in number, their occurrence is known by weathering regime which is fully mapped in the United States by soil taxa. PFDA and long-chain PFCA correlation with pH potentially reflects liming of surface soil, where long-chains are strongly sorbed. Shown are PFHxA with [HAO] (**a**), [Kaolinite] (**b**), [Gibbsite] (**c**), HAO surface charge (**e**), Kaolinite surface charge (**f**), Gibbsite surface charge (**g**) and PFDA with [HFO] (**d**), HFO surface charge (**h**), TOC (**i**), total N (**j**), AWI (**k**), and soil pH (**l**).

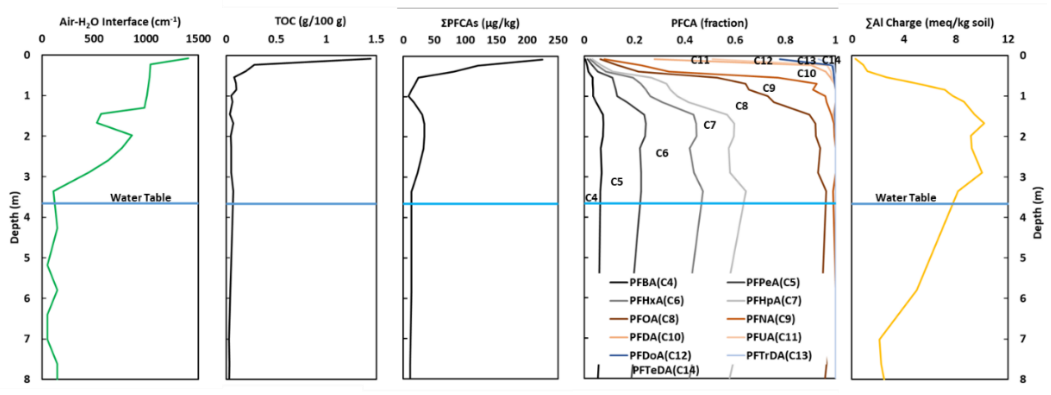

Supplementary Fig. 21. **PFCA vertical distribution in colluvial Field 1, with air-water interface and TOC (clustering with long-chain PFCAs in surface soil) and aluminum-oxide charge (clustering with short-chain PFAS in subsurface soil).**

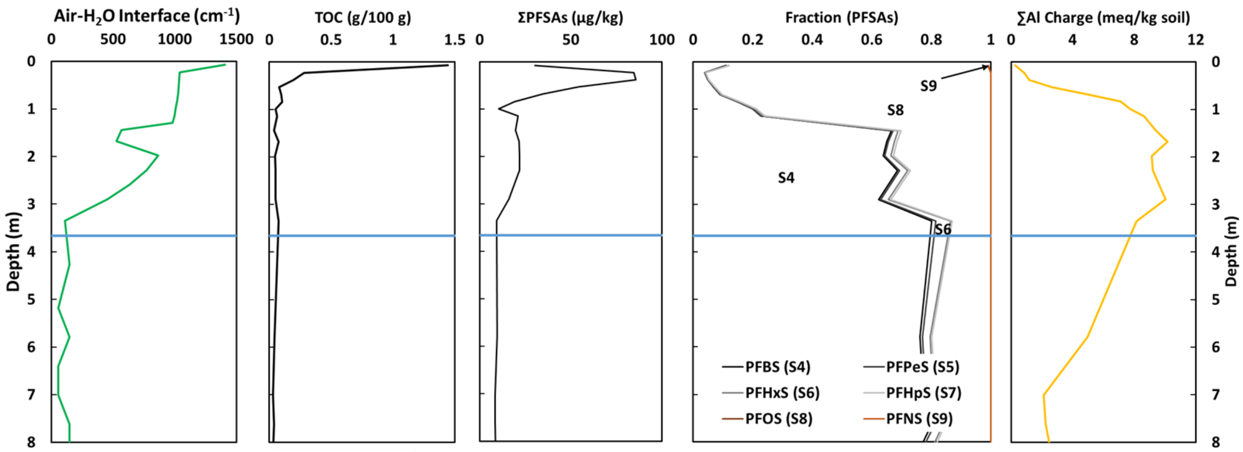

Supplementary Fig. 22. **PFSA vertical distribution in colluvial Field 1, with air-water interface and TOC (clustering with long-chain PFSAs in near-surface soil) and aluminum-oxide charge (clustering with short-chain PFSAs in subsurface soil).**

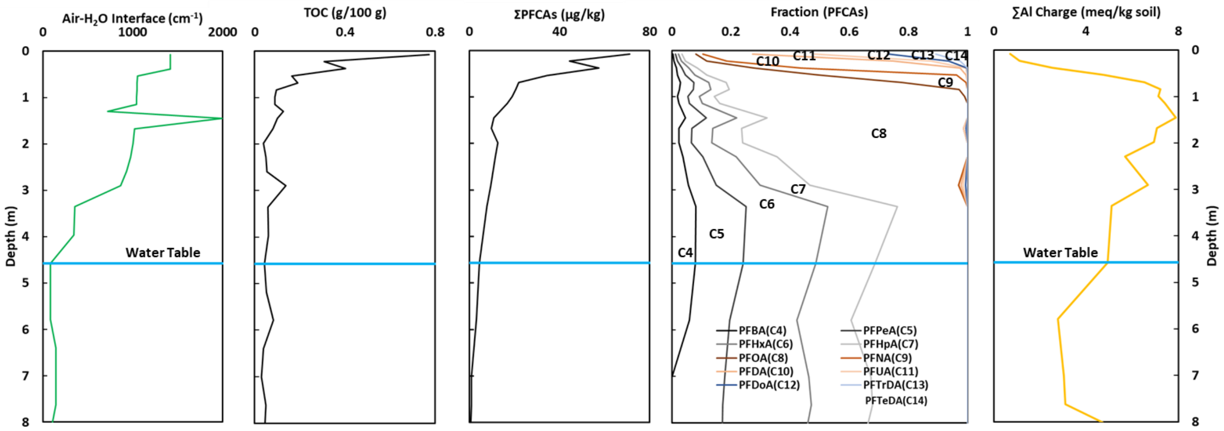

Supplementary Fig. 23. **PFCA vertical distribution in residual Field 2, with air-water interface and TOC (clustering with long-chain PFCAs in surface soil) and aluminum-oxide charge (clustering with short-chain PFAS in subsurface soil).**

		Organic (%)		Hydrostatic State					Aluminum (Oxy-) Hydroxide Mineral(oid)s				Ferric (Oxy-)Hydroxide Mineral(oid)s				Clay Fraction				Silt & Sand Fraction				Exchange Complex					Mehlich Metals						Soluble Anions		
		TOC	Total N	Estimated Hydrostatic Pressure	Estimated Fraction Saturation	Air-Water Interface[1]	Air-Water Interface[2]	Measured Solids Content	Extn Hydrous Al Oxide Conc	Extn Hydrous Al Oxide Charge	X-ray Gibbsite Conc.	X-ray Gibbsite Charge	Extn Hydrous Fe Oxide Conc	Extn Hydrous Fe Oxide Charge	X-ray Goethite Conc.	X-ray Goethite Charge	X-ray Illite Conc	X-ray Kaolinite Conc	X-ray Kaolinite Charge	Textural Clay	Textural Silt	Textural Sand	X-ray Quartz Conc.	Extn Hydrous Mn Oxide Conc	Cation Exchange Capacity	Base Saturation	Lime Buffer Capacity	Sample Depth	Soil pH	LogMehlich Ca	LogMehlich Mg	LogMehlich Na	LogMehlich K	LogMehlich Fe	LogMehlich Pb	LogP	LogCl	LogNO3
All Depths	PFBA	0.05	0.06	-0.46	-0.13	0.15	0.26	0.18	0.63	0.49	0.38	0.34	0.12	0.22	-0.12	-0.13	0.20	0.32	0.27	0.47	0.45	-0.60	-0.27	-0.12	0.20	-0.21	0.27	-0.43	-0.06	0.07	0.20	-0.15	0.50	0.08	0.52	-0.11	0.19	0.13
	PFPeA	0.09	0.05	-0.50	-0.20	0.22	0.31	0.34	0.61	0.48	0.41	0.37	0.17	0.26	-0.01	-0.02	0.10	0.31	0.25	0.44	0.33	-0.52	-0.27	-0.09	0.19	-0.10	0.25	-0.50	-0.03	0.11	0.14	-0.08	0.31	0.16	0.45	-0.10	0.10	0.01
	PFHxA	-0.09	-0.08	-0.30	0.00	0.02	0.12	0.14	0.50	0.55	0.38	0.43	-0.02	0.22	-0.10	-0.06	0.23	0.33	0.36	0.44	0.26	-0.50	-0.28	-0.20	0.25	-0.27	0.33	-0.29	-0.23	-0.11	0.07	-0.15	0.21	0.02	0.42	-0.25	0.30	0.05
	PFHpA	0.04	0.03	-0.46	-0.20	0.21	0.29	0.35	0.59	0.52	0.40	0.40	0.12	0.27	-0.04	-0.03	0.07	0.31	0.28	0.43	0.27	-0.49	-0.25	-0.11	0.23	-0.16	0.28	-0.46	-0.10	0.06	0.15	-0.12	0.23	0.12	0.43	-0.15	0.16	0.03
	PFOA	0.20	0.10	-0.62	-0.44	0.46	0.54	0.58	0.73	0.51	0.34	0.26	0.36	0.48	-0.04	-0.05	-0.27	0.12	0.04	0.34	0.39	-0.47	-0.08	-0.02	0.06	0.02	0.06	-0.61	0.05	0.33	0.35	-0.23	0.43	0.27	0.44	-0.01	-0.04	0.13
	PFNA	0.49	0.35	-0.75	-0.60	0.64	0.69	0.66	0.67	0.13	0.15	-0.11	0.50	0.18	0.15	0.03	-0.34	0.01	-0.24	0.28	0.54	-0.49	-0.02	0.17	0.02	0.28	0.03	-0.75	0.44	0.58	0.41	0.05	0.51	0.41	0.42	0.25	-0.55	0.23
	PFDA	0.77	0.60	-0.78	-0.72	0.77	0.81	0.87	-0.19	-0.82	-0.70	-0.90	0.76	-0.12	-0.39	-0.52	-0.83	-0.76	-0.90	-0.73	0.47	0.67	0.74	0.53	-0.73	0.85	-0.84	-0.81	0.75	0.83	0.08	-0.55	0.29	0.53	-0.16	0.72	-0.61	0.44
	PFUdA	0.88	0.76	-0.68	-0.76	0.82	0.89	0.78	-0.51	-0.66	-0.77	-0.74	0.80	0.28	-0.43	-0.66	-0.83	-0.84	-0.62	-0.88	0.41	0.83	0.81	0.65	-0.19	0.52	-0.69	-0.74	0.34	0.71	-0.09	-0.41	0.26	0.49	-0.17	0.90	-0.45	0.47
	PFDoA	0.90	0.82	-0.64	-0.77	0.80	0.84	0.77	-0.63	-0.70	-0.81	-0.75	0.77	0.15	-0.53	-0.55	-0.80	-0.83	-0.65	-0.91	0.38	0.89	0.83	0.73	-0.25	0.60	-0.70	-0.68	0.45	0.71	-0.14	-0.36	0.31	0.57	-0.26	0.96	-0.41	0.52
	PFOS	0.61	0.50	-0.70	-0.67	0.70	0.74	0.71	0.42	-0.19	-0.02	-0.31	0.55	-0.01	0.16	0.02	-0.46	-0.12	-0.39	0.06	0.46	-0.26	0.11	0.33	-0.05	0.44	-0.06	-0.72	0.62	0.65	0.33	0.09	0.32	0.44	0.29	0.42	-0.65	0.25
Unsaturated Media Above Water Table	PFBA	-0.25	-0.21	0.19	0.36	-0.33	-0.21	-0.34	0.46	0.39	0.23	0.25	-0.19	0.06	-0.24	-0.23	0.35	0.22	0.25	0.32	0.23	-0.45	-0.19	-0.31	-0.10	-0.22	0.06	0.32	-0.27	-0.32	-0.13	-0.37	0.32	-0.11	0.32	-0.34	0.29	0.00
	PFPeA	-0.34	-0.27	0.31	0.46	-0.43	-0.31	-0.47	0.44	0.45	0.31	0.34	-0.25	0.09	-0.18	-0.16	0.46	0.32	0.35	0.40	0.14	-0.50	-0.28	-0.37	-0.01	-0.32	0.18	0.42	-0.36	-0.43	-0.17	-0.29	0.23	-0.16	0.33	-0.41	0.38	-0.06
	PFHxA	-0.44	-0.31	0.43	0.54	-0.52	-0.38	-0.56	0.43	0.58	0.33	0.45	-0.36	0.12	-0.23	-0.16	0.52	0.36	0.47	0.45	0.13	-0.55	-0.31	-0.40	0.17	-0.48	0.33	0.60	-0.51	-0.55	-0.13	-0.29	0.17	-0.23	0.37	-0.48	0.55	0.03
	PFHpA	-0.44	-0.30	0.38	0.49	-0.46	-0.36	-0.50	0.47	0.58	0.31	0.41	-0.36	0.11	-0.25	-0.19	0.46	0.34	0.43	0.44	0.14	-0.55	-0.28	-0.41	0.13	-0.47	0.29	0.55	-0.50	-0.52	-0.09	-0.34	0.18	-0.24	0.37	-0.49	0.53	0.05
	PFOA	-0.24	-0.25	-0.15	0.11	-0.05	0.07	-0.04	0.74	0.56	0.15	0.16	-0.01	0.41	-0.30	-0.27	-0.04	0.03	0.08	0.29	0.43	-0.51	0.00	-0.32	-0.14	-0.25	-0.05	0.08	-0.33	-0.10	0.26	-0.56	0.53	-0.07	0.41	-0.32	0.29	0.27
	PFNA	0.20	0.09	-0.45	-0.24	0.30	0.36	0.33	0.33	-0.20	-0.14	-0.40	0.20	-0.09	0.06	-0.06	-0.30	-0.20	-0.41	-0.05	0.18	-0.02	0.17	-0.03	-0.49	0.34	-0.36	-0.47	0.29	0.28	0.06	-0.24	0.17	0.17	0.07	0.01	-0.54	0.09
	PFDA	0.77	0.60	-0.78	-0.72	0.77	0.81	0.87	-0.19	-0.82	-0.70	-0.90	0.76	-0.12	-0.39	-0.52	-0.83	-0.76	-0.90	-0.73	0.47	0.67	0.74	0.53	-0.73	0.85	-0.84	-0.81	0.75	0.83	0.08	-0.55	0.29	0.53	-0.16	0.72	-0.61	0.44
	PFUdA	0.88	0.76	-0.68	-0.76	0.82	0.89	0.78	-0.51	-0.66	-0.77	-0.74	0.80	0.28	-0.43	-0.66	-0.83	-0.84	-0.62	-0.88	0.41	0.83	0.81	0.65	-0.19	0.52	-0.69	-0.74	0.34	0.71	-0.09	-0.41	0.26	0.49	-0.17	0.90	-0.45	0.47
	PFDoA	0.90	0.82	-0.64	-0.77	0.80	0.84	0.77	-0.63	-0.70	-0.81	-0.75	0.77	0.15	-0.53	-0.55	-0.80	-0.83	-0.65	-0.91	0.38	0.89	0.83	0.73	-0.25	0.60	-0.70	-0.68	0.45	0.71	-0.14	-0.36	0.31	0.57	-0.26	0.96	-0.41	0.52
	PFOS	0.42	0.33	-0.47	-0.41	0.46	0.52	0.50	-0.11	-0.60	-0.35	-0.62	0.30	-0.32	0.09	-0.06	-0.46	-0.35	-0.57	-0.33	0.11	0.33	0.32	0.20	-0.52	0.57	-0.43	-0.57	0.54	0.43	-0.03	-0.15	-0.09	0.23	-0.08	0.27	-0.66	0.12
Saturated Media Below Water Table	PFBA	0.05	0.06	-0.81	-0.18	0.41	0.47	0.20	0.59	0.56	0.40	0.42	-0.16	-0.07	-0.20	-0.19	0.42	0.57	0.57	0.55	0.58	-0.56	-0.47	-0.39	0.71	-0.70	0.74	-0.85	-0.59	0.39	0.48	0.50	0.61	-0.54	0.73	0.22	0.54	0.36
	PFPeA	0.04	-0.28	-0.44	-0.18	0.12	0.03	0.62	0.14	0.13	-0.10	-0.10	-0.25	-0.24	-0.03	-0.03	-0.21	-0.10	-0.10	0.00	0.12	-0.05	0.21	-0.61	0.12	-0.18	0.13	-0.59	-0.03	0.07	-0.15	-0.20	-0.18	-0.31	0.14	-0.29	0.01	-0.04
	PFHxA	0.06	-0.29	-0.42	-0.17	0.10	0.01	0.63	0.13	0.12	-0.10	-0.09	-0.26	-0.25	-0.03	-0.03	-0.22	-0.11	-0.11	-0.01	0.11	-0.04	0.22	-0.62	0.10	-0.14	0.11	-0.57	-0.01	0.06	-0.17	-0.22	-0.19	-0.29	0.12	-0.30	0.00	-0.05
	PFHpA	-0.05	-0.37	-0.26	-0.25	0.11	-0.01	0.71	-0.04	-0.05	-0.14	-0.15	-0.25	-0.25	0.06	0.06	-0.32	-0.20	-0.21	-0.17	-0.06	0.13	0.31	-0.55	-0.06	-0.08	-0.04	-0.44	0.12	-0.08	-0.32	-0.36	-0.34	-0.33	-0.03	-0.44	-0.18	-0.22
	PFOA	-0.17	-0.49	-0.01	-0.32	0.07	-0.10	0.76	-0.29	-0.30	-0.21	-0.24	-0.23	-0.26	0.17	0.16	-0.50	-0.38	-0.40	-0.41	-0.33	0.38	0.47	-0.40	-0.32	0.06	-0.30	-0.21	0.35	-0.27	-0.54	-0.56	-0.56	-0.31	-0.27	-0.65	-0.45	-0.47
	PFNA	-0.21	-0.30	-0.86	-0.54	0.54	0.54	0.24	0.71	0.72	0.31	0.30	-0.43	-0.38	-0.20	-0.20	0.10	0.26	0.25	0.77	0.74	-0.76	-0.15	-0.24	0.69	-0.49	0.70	-0.86	-0.38	0.09	0.20	0.39	0.59	-0.76	0.78	-0.57	0.76	-0.38
	PFDA																																					
	PFUdA																																					
	PFDoA																																					
	PFOS	-0.30	-0.34	-0.88	-0.66	0.66	0.66	0.10	0.84	0.85	0.28	0.29	-0.29	-0.24	-0.31	-0.30	-0.02	0.31	0.31	0.86	0.84	-0.86	-0.14	-0.11	0.81	-0.62	0.82	-0.88	-0.51	0.21	0.41	0.52	0.76	-0.71	0.89	-0.39	0.88	-0.18

Supplementary Fig. 24. **Correlation table/heat map for Field 1+ Field 2 data combined, revealing robust trends of PFAS with geochemical parameters for surface through truncated depth at 10.36 m (34 ft) for Field 1 and 8.53 m (28 ft) for Field 2 (All Depths), for surface to the water table above 12 ft for Field 1 and 14 ft for Field 2 (Unsaturated Media), and below the water table, from 5.49 – 10.36 m (18 – 34 ft) for Field 1 and 4.27 – 8.53 m (14 - 28 ft) for Field 2 (Saturated Media).** Single-test significance levels are found in the legend of Figure 5, where significant positive correlations are shaded red and significant negative correlations are shaded blue, and white cells are nonsignificant. Gray shaded cells with no values indicate no correlation due to too few detections. Air-Water Interface 1 was calculated using Brusseau Equation 4 (Supplementary Equation 19); Air-Water Interface 2 was calculated using Brusseau Equation 5 (Supplementary Equation 20).

		Organic (%)		Hydrostatic State					Aluminum (Oxy-) Hydroxide Mineral(oid)s				Ferric (Oxy-)Hydroxide Mineral(oid)s				Clay Fraction				Silt & Sand Fraction				Exchange Complex					Mehlich Metals						Soluble Anions		
		TOC	Total N	Estimated Hydrostatic Pressure	Estimated Fraction Saturation	Air-Water Interface[1]	Air-Water Interface[2]	Measured Solids Content	Extn Hydrous Al Oxide Conc	Extn Hydrous Al Oxide Charge	X-ray Gibbsite Conc.	X-ray Gibbsite Charge	Extn Hydrous Fe Oxide Conc	Extn Hydrous Fe Oxide Charge	X-ray Goethite Conc.	X-ray Goethite Charge	X-ray Illite Conc	X-ray Kaolinite Conc	X-ray Kaolinite Charge	Textural Clay	Textural Silt	Textural Sand	X-ray Quartz Conc.	Extn Hydrous Mn Oxide Conc	Cation Exchange Capacity	Base Saturation	Lime Buffer Capacity	Sample Depth	Soil pH	LogMehlich Ca	LogMehlich Mg	LogMehlich Na	LogMehlich K	LogMehlich Fe	LogMehlich Pb	LogP	LogCl	LogNO3
All Depths	PFBA	0.09	0.12	-0.47	-0.14	0.16	0.24	0.16	0.57	0.43	0.46	0.40	0.05	-0.05	0.01	-0.05	0.36	0.50	0.36	0.51	0.65	-0.60	-0.44	-0.04	0.29	-0.20	0.40	-0.47	0.03	0.05	-0.01	0.07	0.53	-0.01	0.58	-0.03	0.44	-0.14
	PFPeA	0.34	0.27	-0.74	-0.38	0.39	0.46	0.37	0.71	0.54	0.60	0.51	0.29	0.23	0.26	0.21	0.54	0.61	0.45	0.62	0.76	-0.71	-0.60	0.10	0.57	-0.65	0.62	-0.74	0.13	0.27	0.27	0.39	0.64	0.49	0.76	0.09	0.28	-0.10
	PFHxA	0.26	0.19	-0.65	-0.28	0.28	0.34	0.26	0.61	0.54	0.56	0.53	0.19	0.24	0.20	0.19	0.60	0.59	0.49	0.57	0.68	-0.65	-0.58	0.04	0.54	-0.72	0.60	-0.65	0.02	0.15	0.16	0.34	0.62	0.44	0.74	0.03	0.35	-0.13
	PFHpA	0.32	0.25	-0.72	-0.36	0.36	0.42	0.34	0.67	0.54	0.57	0.52	0.26	0.24	0.25	0.21	0.56	0.60	0.47	0.60	0.72	-0.68	-0.59	0.09	0.58	-0.70	0.62	-0.72	0.09	0.24	0.26	0.39	0.66	0.48	0.75	0.08	0.28	-0.11
	PFOA	0.44	0.35	-0.80	-0.49	0.49	0.55	0.47	0.73	0.46	0.53	0.42	0.38	0.21	0.28	0.22	0.43	0.55	0.36	0.58	0.77	-0.68	-0.55	0.20	0.58	-0.62	0.59	-0.80	0.24	0.38	0.39	0.42	0.70	0.54	0.73	0.19	0.14	-0.03
	PFNA	0.55	0.50	-0.91	-0.70	0.74	0.81	0.69	0.88	0.20	0.43	0.09	0.52	-0.04	0.46	0.27	-0.07	0.31	-0.09	0.57	0.74	-0.68	-0.36	0.27	0.41	0.08	0.42	-0.91	0.56	0.66	0.66	0.49	0.54	0.42	0.46	0.29	-0.29	0.06
	PFDA	0.82	0.73	-0.84	-0.75	0.79	0.80	0.85	0.09	-0.91	-0.68	-0.95	0.84	-0.48	-0.34	-0.55	-0.84	-0.76	-0.95	-0.65	0.31	0.66	0.74	0.62	-0.68	0.94	-0.87	-0.84	0.92	0.93	0.66	-0.65	0.14	0.55	-0.61	0.71	-0.53	0.46
	PFUdA	0.94	0.91	-0.85	-0.84	0.88	0.90	0.78	-0.31	-0.57	-0.85	-0.69	0.95	0.57	-0.54	-0.71	-0.83	-0.93	-0.51	-0.86	0.54	0.85	0.89	0.80	0.18	0.56	-0.72	-0.85	0.43	0.80	0.43	-0.81	0.59	0.66	-0.62	0.85	-0.21	0.63
	PFDoA	0.95	0.92	-0.81	-0.88	0.88	0.89	0.85	-0.32	-0.66	-0.87	-0.72	0.93	0.15	-0.76	-0.69	-0.82	-0.96	-0.59	-0.93	0.61	0.92	0.95	0.90	0.01	0.67	-0.80	-0.81	0.57	0.81	0.41	-0.79	0.77	0.75	-0.76	0.94	-0.11	0.79
	PFTrDA	0.96	0.94	-0.81	-0.90	0.90	0.91	0.87	-0.29	-0.72	-0.90	-0.77	0.94	0.10	-0.81	-0.72	-0.85	-0.96	-0.66	-0.95	0.63	0.94	0.97	0.91	-0.03	0.72	-0.82	-0.81	0.65	0.83	0.54	-0.78	0.67	0.74	-0.79	0.95	-0.28	0.82
	PFBS	0.11	0.06	-0.53	-0.13	0.13	0.19	0.11	0.51	0.57	0.56	0.57	0.05	0.25	0.18	0.18	0.68	0.62	0.56	0.55	0.58	-0.61	-0.60	-0.07	0.50	-0.78	0.59	-0.53	-0.12	0.00	0.03	0.31	0.57	0.37	0.74	-0.10	0.42	-0.20
	PFPeS	-0.25	-0.17	-0.42	0.10	-0.09	0.01	-0.07	0.68	0.93	0.83	0.85	-0.20	0.47	0.15	0.16	0.81	0.91	0.91	0.89	0.37	-0.89	-0.87	-0.38	0.74	-0.42	0.88	-0.42	-0.47	-0.28	-0.24	0.82	0.45	0.41	0.90	-0.39	0.74	-0.49
	PFHxS	-0.16	-0.05	-0.05	0.23	-0.22	-0.19	-0.26	0.15	0.44	0.34	0.49	-0.22	0.05	-0.24	-0.18	0.63	0.40	0.51	0.28	0.35	-0.33	-0.34	-0.19	0.19	-0.48	0.31	-0.05	-0.32	-0.31	-0.34	-0.15	0.39	-0.04	0.45	-0.19	0.64	-0.23
	PFHpS	0.25	0.28	-0.62	-0.35	0.39	0.48	0.31	0.74	0.34	0.39	0.20	0.22	-0.07	0.14	0.01	0.11	0.43	0.18	0.55	0.66	-0.65	-0.38	0.05	0.26	0.05	0.35	-0.62	0.24	0.26	0.25	0.21	0.54	0.43	0.48	0.08	0.14	-0.08
	PFOS	0.66	0.58	-0.91	-0.77	0.81	0.86	0.79	0.85	0.12	0.38	0.02	0.62	-0.06	0.43	0.23	-0.20	0.27	-0.15	0.50	0.76	-0.62	-0.30	0.36	0.41	0.05	0.38	-0.91	0.66	0.76	0.75	0.47	0.63	0.55	0.43	0.40	-0.38	0.13
Unsaturated Media Above Water Table	PFBA	-0.25	-0.12	0.36	0.37	-0.34	-0.31	-0.45	0.26	0.27	0.27	0.26	-0.31	-0.21	-0.21	-0.23	0.46	0.34	0.30	0.27	0.39	-0.36	-0.28	-0.26	-0.20	-0.18	0.07	0.36	-0.23	-0.38	-0.58	-0.41	0.18	-0.22	0.40	-0.25	0.64	-0.22
	PFPeA	-0.34	-0.21	0.42	0.45	-0.41	-0.36	-0.52	0.26	0.35	0.33	0.34	-0.36	-0.10	-0.16	-0.15	0.53	0.41	0.38	0.33	0.36	-0.42	-0.35	-0.34	-0.16	-0.27	0.16	0.42	-0.33	-0.46	-0.64	-0.35	0.10	-0.28	0.49	-0.33	0.67	-0.28
	PFHxA	-0.41	-0.28	0.56	0.52	-0.50	-0.47	-0.62	0.17	0.49	0.37	0.49	-0.44	0.00	-0.21	-0.14	0.64	0.48	0.53	0.35	0.33	-0.44	-0.40	-0.36	-0.01	-0.42	0.27	0.56	-0.47	-0.58	-0.72	-0.28	0.19	-0.31	0.52	-0.36	0.76	-0.31
	PFHpA	-0.39	-0.25	0.51	0.49	-0.48	-0.44	-0.58	0.19	0.44	0.34	0.43	-0.43	-0.07	-0.20	-0.14	0.60	0.47	0.49	0.36	0.29	-0.44	-0.39	-0.35	-0.06	-0.37	0.22	0.51	-0.42	-0.54	-0.67	-0.31	0.18	-0.30	0.49	-0.36	0.73	-0.31
	PFOA	-0.10	0.02	0.14	0.17	-0.13	-0.07	-0.26	0.29	0.13	0.08	0.05	-0.18	-0.29	-0.20	-0.23	0.27	0.21	0.14	0.16	0.42	-0.24	-0.14	-0.14	-0.29	-0.03	-0.04	0.14	-0.09	-0.22	-0.40	-0.44	0.18	-0.24	0.30	-0.13	0.48	-0.13
	PFNA	0.22	0.20	-0.70	-0.36	0.44	0.54	0.37	0.58	-0.44	-0.15	-0.60	0.23	-0.46	0.35	0.08	-0.43	-0.16	-0.58	0.04	-0.02	-0.04	0.10	0.01	-0.59	0.52	-0.33	-0.70	0.52	0.43	0.34	-0.11	-0.45	-0.12	-0.01	0.05	-0.41	0.01
	PFDA	0.82	0.73	-0.84	-0.75	0.79	0.80	0.85	0.09	-0.91	-0.68	-0.95	0.84	-0.48	-0.34	-0.55	-0.84	-0.76	-0.95	-0.65	0.31	0.66	0.74	0.62	-0.68	0.94	-0.87	-0.84	0.92	0.93	0.66	-0.65	0.14	0.55	-0.61	0.71	-0.53	0.46
	PFUdA	0.94	0.91	-0.85	-0.84	0.88	0.90	0.78	-0.31	-0.57	-0.85	-0.69	0.95	0.57	-0.54	-0.71	-0.83	-0.93	-0.51	-0.86	0.54	0.85	0.89	0.80	0.18	0.56	-0.72	-0.85	0.43	0.80	0.43	-0.81	0.59	0.66	-0.62	0.85	-0.21	0.63
	PFDoA	0.95	0.92	-0.81	-0.88	0.88	0.89	0.85	-0.32	-0.66	-0.87	-0.72	0.93	0.15	-0.76	-0.69	-0.82	-0.96	-0.59	-0.93	0.61	0.92	0.95	0.90	0.01	0.67	-0.80	-0.81	0.57	0.81	0.41	-0.79	0.77	0.75	-0.76	0.94	-0.11	0.79
	PFTrDA	0.96	0.94	-0.81	-0.90	0.90	0.91	0.87	-0.29	-0.72	-0.90	-0.77	0.94	0.10	-0.81	-0.72	-0.85	-0.96	-0.66	-0.95	0.63	0.94	0.97	0.91	-0.03	0.72	-0.82	-0.81	0.65	0.83	0.54	-0.78	0.67	0.74	-0.79	0.95	-0.28	0.82
	PFBS	-0.56	-0.43	0.69	0.65	-0.63	-0.61	-0.73	0.15	0.62	0.50	0.63	-0.58	0.10	-0.13	-0.04	0.76	0.62	0.67	0.48	0.21	-0.56	-0.54	-0.48	0.10	-0.57	0.42	0.69	-0.61	-0.72	-0.79	-0.11	0.14	-0.37	0.62	-0.50	0.77	-0.40
	PFPeS	-0.86	-0.77	0.86	0.78	-0.77	-0.78	-0.88	0.28	0.89	0.75	0.78	-0.74	0.33	0.16	0.16	0.92	0.90	0.91	0.82	-0.75	-0.81	-0.87	-0.84	0.44	-0.89	0.77	0.86	-0.91	-0.87	-0.89	0.68	-0.66	-0.22	0.91	-0.86	0.86	-0.71
	PFHxS	-0.32	-0.19	0.67	0.52	-0.52	-0.53	-0.56	-0.04	0.42	0.31	0.48	-0.37	0.05	-0.36	-0.27	0.61	0.42	0.52	0.25	0.35	-0.33	-0.32	-0.27	0.04	-0.38	0.21	0.67	-0.43	-0.52	-0.64	-0.36	0.37	-0.13	0.39	-0.27	0.70	-0.26
	PFHpS	-0.11	0.00	0.03	0.14	-0.10	-0.03	-0.19	0.43	0.10	0.12	-0.01	-0.22	-0.40	-0.05	-0.15	0.23	0.24	0.08	0.25	0.24	-0.32	-0.20	-0.20	-0.29	0.01	-0.02	0.03	-0.02	-0.18	-0.32	-0.33	0.08	-0.32	0.29	-0.19	0.32	-0.19
	PFOS	0.44	0.40	-0.81	-0.53	0.62	0.69	0.59	0.43	-0.66	-0.33	-0.78	0.48	-0.44	0.25	-0.04	-0.65	-0.38	-0.77	-0.18	0.09	0.18	0.32	0.20	-0.66	0.71	-0.51	-0.81	0.70	0.65	0.55	-0.22	-0.34	0.10	-0.21	0.26	-0.60	0.17
Saturated Media Below Water Table	PFBA	-0.31	-0.24	-0.87	-0.61	0.61	0.61	0.33	0.71	0.73	0.44	0.47	-0.29	-0.22	-0.05	-0.04	0.21	0.57	0.58	0.82	0.81	-0.82	-0.45	-0.11	0.87	-0.73	0.88	-0.87	-0.69	0.08	0.31	0.34	0.71	-0.80	0.82	-0.29	0.78	-0.28
	PFPeA	0.83	0.09	-0.92	-0.57	0.57	0.57	0.02	0.73	0.76	0.76	0.75	0.20	0.26	0.41	0.42	0.83	0.69	0.71	0.81	0.73	-0.79	-0.80	-0.50	0.79	-0.88	0.79	-0.92	-0.87	0.09	0.12	0.55	0.50	0.24	0.71	-0.54	0.88	-0.49
	PFHxA	0.84	0.08	-0.92	-0.56	0.56	0.56	0.03	0.72	0.74	0.75	0.75	0.21	0.26	0.42	0.42	0.83	0.69	0.70	0.80	0.71	-0.77	-0.80	-0.51	0.77	-0.88	0.78	-0.92	-0.86	0.08	0.10	0.54	0.51	0.26	0.71	-0.55	0.88	-0.50
	PFHpA	0.83	0.07	-0.92	-0.57	0.57	0.57	0.05	0.72	0.75	0.76	0.75	0.21	0.27	0.42	0.42	0.82	0.71	0.72	0.81	0.72	-0.78	-0.81	-0.50	0.78	-0.88	0.78	-0.92	-0.87	0.10	0.12	0.55	0.53	0.25	0.72	-0.54	0.88	-0.50
	PFOA	0.81	0.01	-0.92	-0.55	0.55	0.55	0.07	0.72	0.74	0.73	0.72	0.24	0.29	0.39	0.39	0.78	0.73	0.73	0.80	0.70	-0.77	-0.81	-0.50	0.76	-0.87	0.76	-0.92	-0.85	0.11	0.12	0.57	0.54	0.27	0.73	-0.56	0.87	-0.53
	PFNA	0.60	0.32	-0.85	-0.52	0.52	0.52	-0.23	0.66	0.68	0.58	0.56	-0.45	-0.36	0.15	0.15	0.73	0.04	0.13	0.71	0.69	-0.71	-0.33	-0.50	0.71	-0.71	0.72	-0.85	-0.69	-0.07	0.08	0.30	0.63	-0.22	0.70	-0.48	0.81	-0.13
	PFDA																																					
	PFUdA																																					
	PFDoA																																					
	PFTrDA																																					
	PFBS	0.83	0.08	-0.92	-0.57	0.57	0.57	0.02	0.73	0.75	0.75	0.75	0.22	0.27	0.40	0.40	0.82	0.70	0.71	0.81	0.72	-0.78	-0.80	-0.50	0.78	-0.89	0.79	-0.92	-0.88	0.10	0.13	0.57	0.53	0.28	0.73	-0.52	0.88	-0.48
	PFPeS	0.37	0.31	-0.19	-0.98	0.98	0.98	-0.44	0.57	0.63	0.61	0.60	0.38	0.39	-0.52	-0.52	-0.04	0.21	0.23	0.82	0.89	-0.85	0.01	0.50	0.98	-0.62	0.99	-0.19	-0.43	0.83	0.96	0.68	0.88	-0.80	0.36	0.09	0.58	0.90
	PFHxS	0.54	0.33	-0.81	-0.64	0.64	0.64	-0.24	0.72	0.75	0.65	0.66	-0.32	-0.22	0.18	0.19	0.71	0.25	0.35	0.77	0.78	-0.78	-0.49	-0.38	0.85	-0.84	0.85	-0.81	-0.85	0.02	0.27	0.34	0.76	-0.23	0.74	-0.21	0.85	0.09
	PFHpS	-0.29	-0.16	-0.65	-0.78	0.78	0.78	-0.83	0.94	0.96	-0.15	-0.05	0.41	0.42	-0.64	-0.61	-0.61	0.23	0.27	0.89	0.90	-0.90	0.16	0.36	0.93	-0.81	0.94	-0.65	-0.67	0.74	0.97	0.86	0.97	-0.22	0.91	0.41	0.93	0.74
	PFOS	0.62	0.14	-0.85	-0.53	0.53	0.53	0.16	0.60	0.63	0.62	0.61	-0.56	-0.47	0.17	0.17	0.44	0.44	0.48	0.71	0.69	-0.70	-0.53	-0.71	0.72	-0.78	0.73	-0.85	-0.72	0.11	0.15	0.57	0.90	0.10	0.78	-0.57	0.86	-0.28

Supplementary Fig. 25. **Correlation table/heat map for Field 1 PFAS with geochemical parameters for surface through truncated depth at 10.36 m (34 ft) (All Depths), for surface to the water table above 12 ft (Unsaturated Media), and below the water table, from 5.49 – 10.36 m (18 – 34 ft) (Saturated Media).** Single-test significance levels are found in the legend of Figure 5, where significant positive correlations are shaded red and significant negative correlations are shaded blue, and white cells are nonsignificant. Gray shaded cells with no values indicate no correlation due to too few detections. Air-Water Interface 1 was calculated using Brusseau Equation 4 (Supplementary Equation 19); Air-Water Interface 2 was calculated using Brusseau Equation 5 (Supplementary Equation 20).

Depth group	PFAS	Organic (%)		Hydrostatic State					Aluminum (Oxy-) Hydroxide Mineral(oid)s				Ferric (Oxy-)Hydroxide Mineral(oid)s				Clay Fraction			
		TOC	Total N	Estimated Hydrostatic Pressure	Estimated Fraction Saturation	Air-Water Interface[1]	Air-Water Interface[2]	Measured Solids Content	Extn Hydrous Al Oxide Conc	Extn Hydrous Al Oxide Charge	X-ray Gibbsite Conc.	X-ray Gibbsite Charge	Extn Hydrous Fe Oxide Conc	Extn Hydrous Fe Oxide Charge	X-ray Goethite Conc.	X-ray Goethite Charge	X-ray Illite Conc	X-ray Kaolinite Conc	X-ray Kaolinite Charge	Textural Clay
All Depths	PFBA	-0.20	-0.25	-0.09	0.04	-0.06	0.04	0.00	0.60	0.40	0.43	0.35	0.11	0.18	0.19	0.19	0.28	0.37	0.27	0.39
	PFPeA	0.19	0.05	-0.63	-0.37	0.36	0.41	0.50	0.68	0.50	0.39	0.32	0.35	0.41	0.10	0.09	-0.19	0.13	0.05	0.35
	PFHxA	-0.01	-0.05	-0.45	-0.18	0.16	0.25	0.32	0.63	0.57	0.46	0.44	0.15	0.26	0.15	0.14	0.01	0.32	0.25	0.48
	PFHpA	0.13	0.06	-0.63	-0.41	0.40	0.43	0.53	0.68	0.54	0.45	0.40	0.29	0.38	0.12	0.12	-0.22	0.20	0.11	0.39
	PFOA	0.22	0.00	-0.78	-0.62	0.61	0.63	0.70	0.82	0.56	0.54	0.41	0.51	0.62	0.18	0.16	-0.47	0.13	-0.03	0.34
	PFNA	0.10	-0.10	-0.17	-0.04	0.03	0.04	0.22	0.21	-0.08	0.02	-0.12	0.05	-0.18	-0.23	-0.28	-0.01	-0.05	-0.20	0.01
	PFDA	0.68	0.21	-0.74	-0.93	0.92	0.93	0.95	-0.43	-0.82	-0.73	-0.88	0.58	-0.01	-0.80	-0.78	-0.86	-0.88	-0.93	-0.84
	PFUdA	0.88	0.55	-0.78	-0.92	0.92	0.92	0.80	-0.67	-0.94	-0.85	-0.96	0.62	0.25	-0.70	-0.69	-0.91	-0.94	-0.89	-1.00
	PFDoA	0.88	0.72	-0.71	-0.82	0.82	0.82	0.69	-0.84	-0.88	-0.83	-0.82	0.58	0.25	-0.64	-0.63	-0.86	-0.87	-0.81	-0.95
	PFOS	0.54	0.31	-0.63	-0.57	0.56	0.57	0.69	-0.31	-0.60	-0.48	-0.62	0.11	-0.50	-0.63	-0.67	-0.49	-0.56	-0.69	-0.51
Unsaturated Media Above Water Table	PFBA	-0.31	-0.42	0.30	0.39	-0.39	-0.26	-0.38	0.59	0.42	0.42	0.36	-0.04	0.06	0.15	0.15	0.49	0.44	0.36	0.47
	PFPeA	-0.39	-0.43	0.46	0.54	-0.54	-0.41	-0.56	0.59	0.53	0.48	0.46	-0.11	0.09	0.15	0.15	0.65	0.54	0.49	0.56
	PFHxA	-0.56	-0.45	0.68	0.68	-0.68	-0.51	-0.72	0.52	0.60	0.56	0.60	-0.35	-0.07	0.23	0.23	0.77	0.70	0.68	0.66
	PFHpA	-0.60	-0.49	0.66	0.59	-0.59	-0.53	-0.66	0.57	0.62	0.59	0.61	-0.35	-0.06	0.18	0.18	0.68	0.73	0.68	0.67
	PFOA	-0.51	-0.70	0.08	0.14	-0.12	-0.11	-0.18	0.80	0.66	0.66	0.57	0.01	0.38	0.20	0.19	0.17	0.58	0.43	0.59
	PFNA	0.10	-0.10	-0.17	-0.04	0.03	0.04	0.22	0.21	-0.08	0.02	-0.12	0.05	-0.18	-0.23	-0.28	-0.01	-0.05	-0.20	0.01
	PFDA	0.68	0.21	-0.74	-0.93	0.92	0.93	0.95	-0.43	-0.82	-0.73	-0.88	0.58	-0.01	-0.80	-0.78	-0.86	-0.88	-0.93	-0.84
	PFUdA	0.88	0.55	-0.78	-0.92	0.92	0.92	0.80	-0.67	-0.94	-0.85	-0.96	0.62	0.25	-0.70	-0.69	-0.91	-0.94	-0.89	-1.00
	PFDoA	0.88	0.72	-0.71	-0.82	0.82	0.82	0.69	-0.84	-0.88	-0.83	-0.82	0.58	0.25	-0.64	-0.63	-0.86	-0.87	-0.81	-0.95
	PFOS	0.54	0.31	-0.63	-0.57	0.56	0.57	0.69	-0.31	-0.60	-0.48	-0.62	0.11	-0.50	-0.63	-0.67	-0.49	-0.56	-0.69	-0.51
Saturated Media Below Water Table	PFBA	-1.00	1.00	-1.00	0.00			1.00	-1.00	-1.00	-1.00	-1.00	1.00	1.00	1.00	1.00	1.00	-1.00	-1.00	-1.00
	PFPeA	0.38	-0.19	-0.99	0.39	-0.39	-0.39	0.96	0.87	0.83	-0.28	-0.23	-0.26	-0.24	-0.65	-0.65	-0.25	-0.45	-0.35	0.59
	PFHxA	0.41	-0.19	-0.99	0.38	-0.38	-0.38	0.95	0.89	0.84	-0.26	-0.21	-0.27	-0.25	-0.66	-0.66	-0.29	-0.41	-0.31	0.61
	PFHpA	0.42	-0.21	-0.99	0.41	-0.41	-0.41	0.95	0.89	0.85	-0.26	-0.21	-0.27	-0.24	-0.64	-0.64	-0.28	-0.43	-0.33	0.62
	PFOA	0.52	-0.29	-0.96	0.47	-0.47	-0.47	0.90	0.94	0.90	-0.19	-0.13	-0.27	-0.24	-0.61	-0.61	-0.33	-0.41	-0.29	0.72
	PFNA																			
	PFDA																			
	PFUdA																			
	PFDoA																			
	PFOS																			

Depth group	PFAS	Silt & Sand Fraction				Exchange Complex					Mehlich Metals						Soluble Anions		
		Textural Silt	Textural Sand	X-ray Quartz Conc.	Extn Hydrous Mn Oxide Conc	Cation Exchange Capacity	Base Saturation	Lime Buffer Capacity	Sample Depth	Soil pH	LogMehlich Ca	LogMehlich Mg	LogMehlich Na	LogMehlich K	LogMehlich Fe	LogMehlich Pb	LogP	LogCl	LogNO3
All Depths	PFBA	-0.19	-0.40	-0.38	-0.26	0.06	-0.09	0.07	-0.09	-0.07	-0.09	0.47	-0.11	0.31	-0.23	-0.16	-0.43	-0.01	-0.47
	PFPeA	0.30	-0.49	-0.09	-0.01	0.12	0.13	0.08	-0.63	0.10	0.30	0.42	-0.42	0.26	0.28	0.47	-0.12	-0.08	0.17
	PFHxA	0.12	-0.58	-0.27	-0.15	0.34	-0.09	0.30	-0.45	-0.10	0.07	0.49	-0.23	0.07	0.08	0.34	-0.30	0.14	0.26
	PFHpA	0.22	-0.50	-0.14	-0.04	0.20	0.05	0.17	-0.63	0.02	0.25	0.45	-0.44	0.12	0.22	0.49	-0.18	0.01	0.24
	PFOA	0.33	-0.49	-0.07	-0.03	-0.04	0.19	-0.08	-0.78	0.14	0.51	0.53	-0.74	0.36	0.30	0.64	-0.10	-0.14	-0.02
	PFNA	-0.07	0.00	0.03	-0.06	-0.51	0.20	-0.46	-0.17	0.21	-0.04	-0.16	-0.71	0.29	0.08	-0.39	-0.19	-0.61	-0.33
	PFDA	0.68	0.73	0.89	0.69	-0.83	0.90	-0.82	-0.74	0.86	0.71	-0.77	-0.38	0.18	0.57	0.51	0.68	-0.66	0.12
	PFUdA	0.73	0.92	0.95	0.79	-0.65	0.81	-0.69	-0.78	0.72	0.76	-0.85	-0.03	0.11	0.76	0.68	0.97	-0.63	0.42
	PFDoA	0.66	0.92	0.88	0.81	-0.58	0.73	-0.63	-0.71	0.65	0.72	-0.84	0.14	0.15	0.82	0.72	0.99	-0.59	0.37
	PFOS	0.29	0.52	0.55	0.41	-0.85	0.68	-0.84	-0.63	0.65	0.43	-0.63	-0.37	-0.18	0.41	-0.11	0.30	-0.83	0.11
Unsaturated Media Above Water Table	PFBA	-0.17	-0.50	-0.46	-0.36	0.16	-0.22	0.18	0.30	-0.19	-0.26	0.50	0.05	0.31	-0.36	-0.33	-0.53	0.09	-0.38
	PFPeA	-0.32	-0.56	-0.56	-0.42	0.33	-0.37	0.33	0.46	-0.36	-0.40	0.54	0.19	0.22	-0.38	-0.41	-0.59	0.23	-0.31
	PFHxA	-0.53	-0.62	-0.70	-0.49	0.58	-0.58	0.58	0.68	-0.54	-0.63	0.58	0.37	-0.06	-0.56	-0.43	-0.72	0.48	-0.09
	PFHpA	-0.56	-0.61	-0.71	-0.53	0.56	-0.63	0.58	0.66	-0.59	-0.63	0.61	0.24	-0.07	-0.63	-0.44	-0.77	0.48	-0.13
	PFOA	-0.20	-0.63	-0.57	-0.57	0.17	-0.41	0.18	0.08	-0.44	-0.11	0.74	-0.42	0.35	-0.54	-0.08	-0.66	0.27	-0.52
	PFNA	-0.07	0.00	0.03	-0.06	-0.51	0.20	-0.46	-0.17	0.21	-0.04	-0.16	-0.71	0.29	0.08	-0.39	-0.19	-0.61	-0.33
	PFDA	0.68	0.73	0.89	0.69	-0.83	0.90	-0.82	-0.74	0.86	0.71	-0.77	-0.38	0.18	0.57	0.51	0.68	-0.66	0.12
	PFUdA	0.73	0.92	0.95	0.79	-0.65	0.81	-0.69	-0.78	0.72	0.76	-0.85	-0.03	0.11	0.76	0.68	0.97	-0.63	0.42
	PFDoA	0.66	0.92	0.88	0.81	-0.58	0.73	-0.63	-0.71	0.65	0.72	-0.84	0.14	0.15	0.82	0.72	0.99	-0.59	0.37
	PFOS	0.29	0.52	0.55	0.41	-0.85	0.68	-0.84	-0.63	0.65	0.43	-0.63	-0.37	-0.18	0.41	-0.11	0.30	-0.83	0.11
Saturated Media Below Water Table	PFBA	-1.00	1.00	1.00	1.00	1.00	-1.00	1.00	-1.00	1.00	-1.00	-1.00	1.00	-1.00	-1.00	-1.00	-1.00	1.00	-1.00
	PFPeA	0.87	-0.75	0.75	-0.78	0.85	-0.53	0.84	-0.99	-0.44	0.51	0.33	-0.48	-0.37	0.39	0.92	0.13	1.00	0.89
	PFHxA	0.88	-0.76	0.72	-0.79	0.84	-0.51	0.84	-0.99	-0.44	0.52	0.35	-0.47	-0.34	0.41	0.92	0.17	1.00	0.90
	PFHpA	0.89	-0.77	0.73	-0.78	0.86	-0.51	0.85	-0.99	-0.46	0.54	0.36	-0.50	-0.33	0.42	0.93	0.17	1.00	0.91
	PFOA	0.92	-0.84	0.68	-0.75	0.90	-0.44	0.88	-0.96	-0.53	0.61	0.43	-0.57	-0.21	0.51	0.97	0.30	0.98	0.96
	PFNA																		
	PFDA																		
	PFUdA																		
	PFDoA																		
	PFOS																		

Supplementary Fig. 26. **Correlation table/heat map for Field 2 PFAS with geochemical parameters for surface through truncated depth at 8.53 m (28 ft) (All Depths), for surface to the water table above 14 ft (Unsaturated Media), and below the water table, from 4.27 – 8.53 m (14 – 28 ft) (Saturated Media).** Single-test significance levels are found in the legend of Figure 5, where significant positive correlations are shaded red and significant negative correlations are shaded blue, and white cells are nonsignificant. Gray shaded cells with no values indicate no correlation due to too few detections. Air-Water Interface 1 was calculated using Brusseau Equation 4 (Supplementary Equation 19); Air-Water Interface 2 was calculated using Brusseau Equation 5 (Supplementary Equation 20).

V. Supplementary References:

1 Owens, J. P. in *Miscellaneous Investigations Series. Map 1-1948-A (Sheet 1 of 2)* (Department of the Interior, US Geological Survey, Denver, CO, 1989).

2 USDA. *Soil Surveys by State*, < https://www.nrcs.usda.gov/conservation-basics/natural-resource-concerns/soil/soil-surveys-by-state> (2023).

3 Kissel, D. E. & Sonon, L. Soil Test Handbook for Georgia. 90 (University of Georgia, College of Agricultural & Environmental Sciences, Athens, GA, 2011).

4 Adeyemi, O. *Mineralogy of per- and polyfluoroalkyl substances (PFAS) contaminated soils from agricultural fields in Society Hill, South Carolina* Master of Science thesis, University of Georgia, (2023).

5 Reitveld, H. M. Line profiles of neutron powder-diffraction peaks for structure refinement. *Acta Crystallographica* **22**, 151-152 (1966).

6 Schroeder, P. A. *Clays in the Critical Zone*. (Cambridge University Press, 2018).

7 Austin, J. C., Richter, D. D. & Schroeder, P. A. Quantification of mixed-layer clays in multiple saturation states using NEWMOD2: Implications for the potassium uplift hypothesis in the SE United States. *Clays and Clay Minerals* **68**, 67-80, doi:10.1007/s42860-019-00060 (2020).

8 Chao, T. T. & Zhou, L. Extraction Techniques for Selective Dissolution of Amorphous Iron Oxides from Soils and Sediments. *Soil Science Society of America Journal* **47**, 225-232 (1983).

9 Poulton, S. W. & Canfield, D. E. Development of a sequential extraction procedure for iron: implications for iron partitioning in continentally derived particulates. *Chemical Geology including Isotope Geoscience* **214**, 209-221 (2005).

10 USEPA. Method 200.8: Determination of Trace Elements in Waters and Wastes by Inductively Coupled Plasma - Mass Spectrometry. (USEPA, Cincinnati, OH 45268, 1994).

11 ASTM. Standard Test Method for Determination of Polyfluorinated Compounds in Soil by Liquid Chromatography Tandem Mass Spectrometry (LC/MS/MS). 24 (ASTM International, West Conshohocken, PA, 2017).

12 McCord, J. & Strynar, M. Identifying Per- and Polyfluorinated Chemical Species with a Combined Targeted and Non-Targeted-Screening High-Resolution Mass Spectrometry Workflow. *J Vis Exp*, doi:10.3791/59142 (2019).

13 ASTM. Standard Test Method for Determination of Per- and Polyfluoroalkyl Substances in Water, Sludge, Influent, Effluent, and Wastewater by Liquid Chromatography Tandem Mass Spectrometry (LC/MS/MS). 28 (ASTM International, West Conshohocken, Pennsylvania, 2019).

14 Evich, M. G. *et al.* Environmental Fate of Cl-PFPECAs: Predicting the Formation of PFAS Transformation Products in New Jersey Soils. *Environ Sci Technol* **56**, 7779–7788, doi:10.1021/acs.est.1c06126 (2022).

15 NCSS. *National Cooperative Soil Survey Soil Characterization Database.*, <ncsslabdatamart.sc.egov.usda.gov> (2023).

16 RStudio: Integrated Development Environment for R (Posit Software, PBC, Boston, MA, 2022).

17 Wickham H, A. M., Bryan J, Chang W, McGowan LD, François R, Grolemund G, Hayes A, Henry L, Hester J, Kuhn M, Pedersen TL, Miller E, Bache SM, Müller K, Ooms J, Robinson D, Seidel DP, Spinu V, Takahashi K, Vaughan D, Wilke C, Woo K, Yutani H. Welcome to the tidyverse. *Journal of Open Source Software* **4**, 1686, doi:doi:10.21105/joss.01686 (2019).

18 readxl v. R package version 1.4.3 (2023).

19 Soil Survey Staff. *Soil Survey Investigations* (U.S. Department of Agriculture, Natural Resources Conservation Service, Soil Survey Investigations Report No. 42, Version 6.0, 2022).

20 Dzombak, D. A. & Morel, F. M. M. *Surface Complexation Modeling: Hydrous Ferric Oxide*. (John Wiley & Sons, 1990).

21 Karamalidis, A. K. & Dzombak, D. A. *Surface Complexation Modeling: Gibbsite*. (John Wiley & Sons, 2010).

22 Chang, H., Lozier, E. H., Ma, E. & Geiger, F. M. Quantification of Stern Layer Water Molecules, Total Potentials, and Energy Densities at Fused Silica:Water Interfaces for Adsorbed Alkali Chlorides, CTAB, PFOA, and PFAS. *Journal of Physical Chemistry A*, doi:10.1021/acs.jpca.3c04434 (2023).

23 Washington, J. W., Endale, D. M., Samarkina, L. P. & Chappell, K. E. Kinetic control of oxidation state at thermodynamically buffered potentials in subsurface waters. *Geochimica et Cosmochimica Acta* **68**, 4831-4842 (2004).

24 Nordstrom, D. K. & Ball, J. W. The geochemical behavior of aluminum in acidified surface waters. *Science* **232**, 54-56 (1986).

25 Hem, J. D. *Study and Interpretation of the Chemical Characteristics of Natural Water*. Third edn, Vol. 2254 263 (US Geological Survey, 1985).

26 Polemio, M., Bufo, S. & Paoletti, S. Evaluation of ionic strength and salinity of groundwaters: effect of the ionic composition. *Geochimica et Cosmochimica Acta* **44**, 809-814 (1980).

27 Mathur, S. S. & Dzombak, D. A. in *Surface Complexation Modeling* (ed J. Lutzenkirchen) Ch. 16, 443-468 (Elsevier Ltd., 2006).

28 Gu, C. & Karthikeyan, K. G. Interaction of Tetracycline with Aluminum and Iron Hydrous Oxides. *Environmental Science & Technology* **39**, 2660-2667 (2005).

29 Tombacz, E. & Szekeres, M. Surface charge heterogeneity of kaolinite in aqueous suspension in comparison with montmorillonite. *Applied Clay Science* **34**, 105-124 (2006).

30 Kumar, N., Andersson, M. P., van den Ende, D., Mugele, F. & Siretanu, I. Probing the surface charge on the basal planes of kaolinite particles with high-resolution atomic force microscopy. *Langmuir* **33**, 14226-14237 (2017).

31 Dixon, J. B. in *Minerals in Soil Environments* (eds J.B. Dixon & S.B. Weed) 467-526 (Soil Science Society of America, 1989).

32 Gupta, V. & Miller, J. D. Surface force measurements at the basal planes of ordered kaolinite. *Journal of Colloid and Interface Science* **344**, 362-371 (2010).

33 Ahmad, A., Tian, K., Tanyu, B. & Foster, G. D. Effect of Clay Mineralogy on the Partition Coefficients of Perfluoroalkyl Substances. *ES&T Water*, doi:10.1021/acsestwater.3c00105 (2023).

34 Brusseau, M. L. The influence of molecular structure on the adsorption of PFAS to fluid-fluid interfaces: Using QSPR to predict interfacial adsorption coefficients. *Water research* **152**, 148-158, doi:https://doi.org/10.1016/j.watres.2018.12.057 (2019).

35 Brusseau, M. L. Determining air-water interfacial areas for the retention and transport of PFAS and other interfacially active solutes in unsaturated porous media. *Science of The Total Environment* **884**, 163730, doi:10.1016/j.scitotenv.2023.163730 (2023).

36 Van Glubt, S. & Brusseau, M. L. Contribution of Nonaqueous-Phase Liquids to the Retention and Transport of Per and Polyfluoroalkyl Substances (PFAS) in Porous Media. *Environmental Science & Technology* **55**, 3706-3715, doi:10.1021/acs.est.0c07355 (2021).

37 Arthur, E. *et al.* Soil Specific Surface Area and Non-Singularity of Soil-Water Retention at Low Saturations. *Soil Science Society of America Journal* **77**, 43-53, doi:10.2136/sssaj2012.0262 (2012).

38 Dosskey, M. G., Helmers, M. J. & Eisenhauer, D. E. An approach for using soil surveys to guide the placement of water quality buffers. *Journal of Soil and Water Conservation* **61**, 344-354 (2006).

39 Bumb, A. C., Murphy, C. L. & Everett, L. G. A comparison of three functional forms for representing soil moisture characteristics. *Groundwater* **30**, 177-185 (1992).

40 Nesbitt, H. W. & Young, G. M. Formation and diagenesis of weathering profiles. *The Journal of Geology* **97**, 129-147 (1989).

41 Nesbitt, H. W. & Markovics, G. Weathering of granodioritic crust, long-term storage of elements in weathering profiles, and petrogenesis of siliciclastic sediments. *Geochimica et Cosmochimica Acta* **61**, 1653-1670 (1997).

42 Nesbitt, H. W. & Young, G. M. Prediction of some weathering trends of plutonic and volcanic rocks based on thermodynamic and kinetic considerations. *Geochimica et Cosmochimica Acta* **48**, 1523-1534 (1984).

43 Nesbitt, H. W. & Wilson, R. E. Recent Chemical Weathering of Basalts. *American Journal of Science* **292**, 740-777 (1992).

44 Ito, A. & Wagai, R. Global distribution of clay-size minerals on land surface for biogeochemical and climatological studies. *Scientific Data* **4**, doi:10.1038/sdata.2017.103 (2017).

45 Steefel, C. I. & Van Cappellen, P. A new kinetic approach to modeling water-rock interaction: The role of nucleation, precursors, and Ostwald ripening. *Geochimica et Cosmochimica Acta* **54**, 2657-2677 (1990).

46 Berner, R. A. in *Kinetics of Geochemical Processes* Vol. 8 *Reviews in Mineralogy* (eds A.C. Lasaga & R.J. Kirkpatrick) Ch. 3, 111-134 (Mineralogical Society of America, 1983).

47 The Geochemist's Workbench (Research Park at the University of Illinois Urbana-Champaign, 2023).

48 Nordstrom, D. K., Valentine, S. D., Ball, J. W., Plummer, L. N. & Jones, B. F. Partial Compilation and Revision of Basic Data in the WATEQ Programs. Report No. Water Resources Investigation Report 84-4186, (U.S. Geological Survey, Menlo Park, CA, 1984).

49 Singer, P. C. & Stumm, W. Acidic mine drainage: the rate-determining step. *Science* **167**, 1121-1123 (1970).

50 Benezeth, P., Palmer, D. A. & Wesolowski, D. J. Dissolution/precipitation kinetics of boehmite and gibbsite: Application of a pH-relaxation technique to study near-equilibrium rates. *Geochimica et Cosmochimica Acta* **72**, 2429-2453 (2008).

51 Evich, M. G. *et al.* Per- and polyfluoroalkyl substances in the environment. *Science* **375**, eabg9065, doi:10.1126/science.abg9065 (2022).

52 Hepburn, E. *et al.* Contamination of groundwater with per- and polyfluoroalkyl substances (PFAS) from legacy landfills in an urban re-development precinct. *Environmental Pollution* **248**, 101-113, doi:https://doi.org/10.1016/j.envpol.2019.02.018 (2019).

53 Kelessidis, A. & Stasinakis, A. S. Comparative study of the methods used for treatment and final disposal of sewage sludge in European countries. *Waste Manag* **32**, 1186-1195, doi:10.1016/j.wasman.2012.01.012 (2012).

54 Arvaniti, O. S. & Stasinakis, A. S. Review on the occurrence, fate and removal of perfluorinated compounds during wastewater treatment. *Science of The Total Environment* **524-525**, 81-92, doi:https://doi.org/10.1016/j.scitotenv.2015.04.023 (2015).

55 Borthakur, A., Leonard, J., Koutnik, V. S., Ravi, S. & Mohanty, S. K. Inhalation risks of wind-blown dust from biosolid-applied agricultural lands: Are they enriched with microplastics and PFAS? *Current Opinion in Environmental Science & Health* **25**, 100309, doi:https://doi.org/10.1016/j.coesh.2021.100309 (2022).

56 Liu, S. *et al.* The Fate and Transport of Chlorinated Polyfluorinated Ether Sulfonates and Other PFAS through Industrial Wastewater Treatment Facilities in China. *Environmental Science & Technology* **56**, 3002-3010, doi:10.1021/acs.est.1c04276 (2022).

57 Chen, S., Zhou, Y., Meng, J. & Wang, T. Seasonal and annual variations in removal efficiency of perfluoroalkyl substances by different wastewater treatment processes. *Environmental Pollution* **242**, 2059-2067, doi:https://doi.org/10.1016/j.envpol.2018.06.078 (2018).

58 Jia, Y., Shan, C., Fu, W., Wei, S. & Pan, B. Occurrences and fates of per- and polyfluoralkyl substances in textile dyeing wastewater along full-scale treatment processes. *Water research* **242**, 120289, doi:https://doi.org/10.1016/j.watres.2023.120289 (2023).

59 Dunn, M. *et al.* Unregulated Active and Closed Textile Mills Represent a Significant Vector of PFAS Contamination into Coastal Rivers. *ES&T Water*, doi:10.1021/acsestwater.3c00439 (2023).

60 Costello, M. C. S. & Lee, L. S. Sources, Fate, and Plant Uptake in Agricultural Systems of Per- and Polyfluoroalkyl Substances. *Current Pollution Reports*, doi:https://doi.org/10.1007/s40726-020-00168-y (2020).

61 Li, H. *et al.* Transport behavior difference and transport model of long- and short-chain per- and polyfluoralkyl substances in underground environmental media: A review. *Environmental Pollution* **327**, 121579, doi:https://doi.org/10.1016/j.envpol.2023.121579 (2023).

62 Davis, M. J. B., Evich, M. G., Goodrow, S. M. & Washington, J. W. Environmental Fate of Cl-PFPECAs: Accumulation of Novel and Legacy Perfluoroalkyl Compounds in Real-World Vegetation and Subsoils. *Environmental Science & Technology*, doi:10.1021/acs.est.3c00665 (2023).

63 Washington, J. W., Yoo, H., Ellington, J. J., Jenkins, T. M. & Libelo, E. L. Concentrations, Distribution, and Persistence of Perfluoroalkylates in Sludge-Applied Soils near Decatur, Alabama, USA. *Environmental Science & Technology* **44**, 8390-8396 (2010).

64 Johnson, G. R. PFAS in soil and groundwater following historical land application of biosolids. *Water research* **211**, 118035, doi:https://doi.org/10.1016/j.watres.2021.118035 (2022).

65 Xing, Y. *et al.* PFASs in Soil: How They Threaten Human Health through Multiple Pathways and Whether They Are Receiving Adequate Concern. *Journal of Agricultural and Food Chemistry* **71**, 1259-1275, doi:10.1021/acs.jafc.2c06283 (2023).

66 Yong, Z. Y., Kim, K. Y. & Oh, J.-E. The occurrence and distributions of per- and polyfluoroalkyl substances (PFAS) in groundwater after a PFAS leakage incident in 2018. *Environmental Pollution* **268**, 115395, doi:https://doi.org/10.1016/j.envpol.2020.115395 (2021).

67 USEPA. Lifetime Health Advisories and Health Effects Support Documents for Perfluorooctanoic Acid and Perfluorooctane Sulfonate, EPA–HQ–OW–2014–0138; FRL–9946–91–OW. *Federal Register* **81** (2016).

68 USEPA. Lifetime Drinking Water Health Advisories for Four Perfluoroalkyl Substances, FRL 9855–01–OW. *Federal Register* **81** (2022).

69 SCDHEC. *Expanded Site Inspection (ESI), Galey and Lord*. (South Carolina Department of Health and Environmental Control, 2018).

70 USEPA. Multi-Industry Per- and Polyfluoroalkyl Substances (PFAS) Study - 2021 Preliminary Report. Report No. EPA-821-R-21-004, 81 (USEPA, 2021).

71 Vierke, L., Moeller, A. & Klitzke, S. Transport of perfluoroalkyl acids in a water-saturated sediment column investigated under near-natural conditions. *Environmental Pollution* **186**, 7-13, doi:10.1016/j.envpol.2013.11.011 (2014).

72 Liu, C. & Liu, J. Aerobic biotransformation of polyfluoroalkyl phosphate esters (PAPs) in soil. *Environmental Pollution* **212**, 230-237, doi:https://doi.org/10.1016/j.envpol.2016.01.069 (2016).

73 Mejia Avendaño, S. & Liu, J. Production of PFOS from aerobic soil biotransformation of two perfluoroalkyl sulfonamide derivatives. *Chemosphere* **119**, 1084-1090, doi:https://doi.org/10.1016/j.chemosphere.2014.09.059 (2015).

74 Allred, B. M., Lang, J. R., Barlaz, M. A. & Field, J. A. Physical and Biological Release of Poly- and Perfluoroalkyl Substances (PFASs) from Municipal Solid Waste in Anaerobic Model Landfill Reactors. *Environmental Science & Technology* **49**, 7648-7656, doi:10.1021/acs.est.5b01040 (2015).

75 Huang, S. & Jaffé, P. R. Defluorination of Perfluorooctanoic Acid (PFOA) and Perfluorooctane Sulfonate (PFOS) by Acidimicrobium sp. Strain A6. *Environmental Science & Technology* **53**, 11410-11419, doi:10.1021/acs.est.9b04047 (2019).

76 Hamid, H., Li, L. Y. & Grace, J. R. Review of the fate and transformation of per- and polyfluoroalkyl substances (PFASs) in landfills. *Environmental Pollution* **235**, 74-84, doi:https://doi.org/10.1016/j.envpol.2017.12.030 (2018).

77 Rhoads, K. R., Janssen, E. M. L., Luthy, R. G. & Criddle, C. S. Aerobic biotransformation and fate of N-ethyl perfluorooctane sulfonamidoethanol (N-EtFOSE) in activated sludge. *Environmental Science & Technology* **42**, 2873-2878, doi:10.1021/es702866c (2008).

78 USEPA. PFAS Environmental Contamination Associated with Manufacturing Sites in New Hampshire: Laboratory Data Report #5: Non-Targeted PFAS Measurements in Commercial Dispersions and Surfactants. 8 (USEPA, 2019).

79 Washington, J. W., Jenkins, T. M., Rankin, K. & Naile, J. E. Decades-scale degradation of commercial, side-chain, fluorotelomer-based polymers in soils & water. *Environmental Science & Technology* **49**, 915-923 (2015).

80 Yoo, H., Washington, J. W., Ellington, J. J., Jenkins, T. M. & Neill, M. P. Concentrations, Distribution, and Persistence of Fluorotelomer Alcohols in Sludge-Applied Soils near Decatur, Alabama, USA. *Environmental Science & Technology* **44**, 8397-8402, doi:10.1021/es100390r (2010).

81 Du, Z. *et al.* Adsorption behavior and mechanism of perfluorinated compounds on various adsorbents—A review. *Journal of Hazardous Materials* **274**, 443-454, doi:https://doi.org/10.1016/j.jhazmat.2014.04.038 (2014).

82 Saeidi, N., Kopinke, F.-D. & Georgi, A. Understanding the effect of carbon surface chemistry on adsorption of perfluorinated alkyl substances. *Chemical Engineering Journal* **381**, 122689, doi:https://doi.org/10.1016/j.cej.2019.122689 (2020).

83 Lyu, X. *et al.* Per- and Polyfluoroalkyl Substances (PFAS) in Subsurface Environments: Occurrence, Fate, Transport, and Research Prospect. *Reviews of Geophysics* **60**, e2021RG000765. 000710.001029/002021RG000765, doi:https://doi.org/10.1029/2021RG000765 (2022).

84 Kabiri, S. *et al.* Physical and chemical properties of carbon-based sorbents that affect the removal of per- and polyfluoroalkyl substances from solution and soil. *Science of The Total Environment* **875**, 162653, doi:https://doi.org/10.1016/j.scitotenv.2023.162653 (2023).

85 Kogel-Knabner, I. *et al.* Organo-mineral associations in temperate soils: Integrating biology, mineralogy, and organic matter chemistry. *J. Plant Nutr. Soil Sci.* **171**, 61-82 (2008).

86 Suda, A. & Makino, T. Functional effects of manganese and iron oxides on the dynamics of trace elements in soils with a special focus on arsenic and cadmium: A review. *Geoderma* **270**, 68-75, doi:https://doi.org/10.1016/j.geoderma.2015.12.017 (2016).

87 Brusseau, M. L. Influence of chain length on field-measured distributions of PFAS in soil and soil porewater. *Journal of Hazardous Materials Letters* **4**, 100080, doi:10.1016/j.hazl.2023.100080 (2023).

88 Higgins, C. P. & Luthy, R. G. Sorption of perfluorinated surfactants on sediments. *Environmental Science & Technology* **40**, 7251-7256, doi:10.1021/es061000n (2006).

89 Johnson, R. L., Anschutz, A. J., Smolen, J. M., Simcik, M. F. & Penn, R. L. The Adsorption of Perfluorooctane Sulfonate onto Sand, Clay, and Iron Oxide Surfaces. *Journal of Chemical & Engineering Data* **52**, 1165-1170, doi:10.1021/je060285g (2007).

90 Jeon, J., Kannan, K., Lim, B. J., An, K. G. & Kim, S. D. Effects of salinity and organic matter on the partitioning of perfluoroalkyl acid (PFAs) to clay particles. *Journal of Environmental Monitoring* **13**, 1803-1810, doi:10.1039/c0em00791a (2011).

91 Zhang, R., Yan, W. & Jing, C. Mechanistic study of PFOS adsorption on kaolinite and montmorillonite. *Colloids and Surfaces A: Physicochemical and Engineering Aspects* **462**, 252-258, doi:https://doi.org/10.1016/j.colsurfa.2014.09.019 (2014).

92 Zhao, L., Bian, J., Zhang, Y., Zhu, L. & Liu, Z. Comparison of the sorption behaviors and mechanisms of perfluorosulfonates and, perfluorocarboxylic acids on three kinds of clay minerals. *Chemosphere* **114**, 51-58, doi:10.1016/j.chemosphere.2014.03.098 (2014).

93 Xiao, F., Zhang, X., Penn, L., Gulliver, J. S. & Simcik, M. F. Effects of Monovalent Cations on the Competitive Adsorption of Perfluoroalkyl Acids by Kaolinite: Experimental Studies and Modeling. *Environmental Science & Technology* **45**, 10028-10035, doi:10.1021/es202524y (2011).

94 Tang, C. Y., Fu, Q. S., Gao, D. W., Criddle, C. S. & Leckie, J. O. Effect of solution chemistry on the adsorption of perfluorooctane sulfonate onto mineral surfaces. *Water research* **44**, 2654-2662, doi:10.1016/j.watres.2010.01.038 (2010).

95 Wang, F. & Shih, K. Adsorption of perfluorooctanesulfonate (PFOS) and perfluorooctanoate (PFOA) on alumina: Influence of solution pH and cations. *Water research* **45**, 2925-2930, doi:10.1016/j.watres.2011.03.007 (2011).

96 Lyu, X. *et al.* Importance of Al/Fe oxyhydroxide coating and ionic strength in perfluorooctanoic acid (PFOA) transport in saturated porous media. *Water research* **175**, 115685, doi:https://doi.org/10.1016/j.watres.2020.115685 (2020).

97 Loganathan, N. & Wilson, A. K. Adsorption, Structure, and Dynamics of Short- and Long-Chain PFAS Molecules in Kaolinite: Molecular-Level Insights. *Environmental Science & Technology* **56**, 8043-8052, doi:10.1021/acs.est.2c01054 (2022).
